# A chromatography-free one-pot, two-step synthesis of 1,2,4-thiadiazoles from primary amides *via* thiolation and oxidative dimerization under solvent-free conditions: a greener approach†

**DOI:** 10.1039/d4ra03993a

**Published:** 2024-07-16

**Authors:** Khushbu Rajput, Vishal Singh, Sundaram Singh, Vandana Srivastava

**Affiliations:** a Department of Chemistry, Indian Institute of Technology (BHU) Varanasi-221005 India vsrivastava.apc@iitbhu.ac.in

## Abstract

An efficient and practical one-pot, two-step synthesis of 1,2,4-thiadiazoles from primary amides with Lawesson reagent (LR) and *tert*-butyl hydrogen peroxide (TBHP) without solvent is demonstrated for the first time. This groundbreaking and environmentally friendly approach utilises readily available starting materials and eliminates the use of traditional solvents in the reaction process. The broad substrate scope, excellent functional group tolerance in mild and metal-free conditions, quick conversion, and excellent yields are essential features of this methodology. All the compounds were purified without column chromatography.

## Abstract 2

Synthesis of 1,2,4-thiadiazoles using Lawesson's reagent and TBHP *via* thiolation and oxidative dimerization of primary amides. This versatile and efficient method demonstrates excellent tolerance for various functional groups in amides.

## Graphical abstract

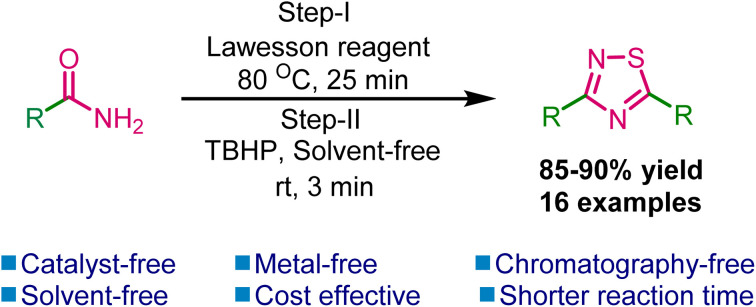

Over the past few decades, the formation of S–N bonds has captured the enthusiastic attention of organic chemists due to their crucial role as fundamental building blocks.^1^ Among these, 1,2,4-thiadiazole is a highly significant heterocyclic compound in the field of medicinal chemistry and agrochemical research.^2^ Its unique molecular structure, consisting of a five-membered ring containing sulfur and nitrogen atoms, offers a robust foundation for designing compounds with specific biological activities.^3^ For example, the 1,2,4-thiadiazole unit containing compounds I and II has much higher inhibitory activity against aromatase than resveratrol, and compound III, shows neuroprotective properties,^4^ and compound IV may be a potential drug for Alzheimer's disease due to its good β-secretion inhibitory activity.^5^ 3,5-Bis(indolyl)-1,2,4-thiadiazoles derivatives (*e.g.*, compound V) display *in vitro* anticancer activity,^6^ (Fig. 1).

**Fig. 1:**
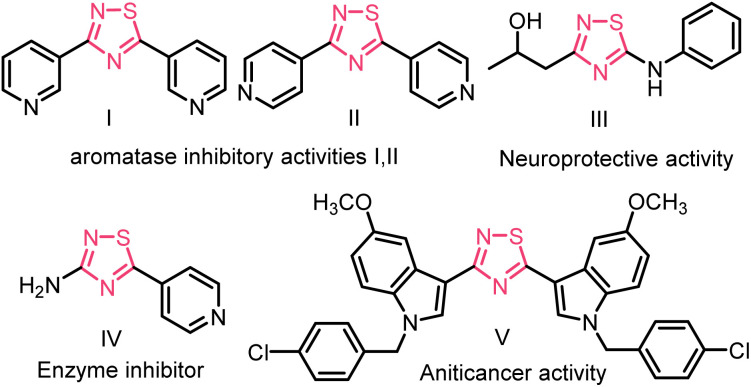
Some biologically active 1,2,4-thiadiazoles.

Given its importance, extensive efforts have been dedicated to developing synthetic methodologies for the production of diverse thiadiazoles. Among the various approaches explored, the oxidative dimerization of thioamides stands out as the most promising method. This technique presents a straightforward and expeditious route, exhibiting favorable atom economy in the synthesis of thiadiazoles.

Various metal and metal-free oxidizing reagents, such as ceric ammonium nitrate,^7^ 2-iodoxybenzoic acid,^8^ nitrous acid,^9^ copper-catalyzed,^10^*N*-bromosuccinimide,^11^ oxone,^12^*tert*-butyl nitrite,^13^ TCT-DMSO,^14^ polymer-supported diarylselenoxide,^15^ chloranil,^16^ copper-oxide,^17^ TCCA,^18^ p-toluenesulfinic acid,^19^ tetra(*n*-butyl)ammonium peroxydisulfate,^20^ organotellurium,^21^ phenyliodine(iii) diacetate,^22^ NH_4_I,^23^*N*-benzyl-DABCO tribromide/DMSO,^24^ eosin Y/light,^25^ H5IO6,^26^ O_2_/I_2_/H_2_SO_4,_^27^ H_6_PV_3_Mo_9_O_40,_^28^ and IBA/Tf_2_O^29^ have been used for dimerization of thioamides. Other substrates, such as aryl nitriles, aryl amidines have also been reported for the synthesis of thiadiazoles.^30^ In 2018, Pan and co-workers developed an electrochemical method for the construction of intermolecular S–N bond from thioamides in the absence of oxidants. However, many of these processes are hampered by the use of excessive reagents, which result in massive amounts of by-products, harsh reaction conditions, prolonged reaction durations, time-consuming workup procedures.^31^ All the above methods required chromatography purification that required large amount of solvents. Developing such a methodology would indeed be highly desirable as it could potentially streamline the synthesis process, reduce the consumption of solvents, lower costs, and make the synthesis more sustainable overall. This pursuit aligns with the principles of green chemistry, aiming to minimize waste and environmental impact while maximizing efficiency and resource utilization.

Amides, characterized by their ready availability, affordability, versatility, stability, biochemical relevance, and natural occurrence,^32^ serve as pivotal starting materials in various organic transformations. They can be diversified into different functional groups through processes such as transamidation,^33^ esterifications,^34^ and cross-coupling reactions.^35^ These attributes make amides particularly advantageous when compared to their thio-analog, thioamides. Lawesson reagent (LR) a potent, mild, and versatile thionating reagent,^36^ is commercially accessible and cost-effective. Its primary use involves replacing oxygen with sulfur in amides, resulting in the formation of thioamides, and also in ester functions. TBHP is a well-known oxidant^37^ and has received much attention in many oxidation processes to form new C–C, C–N, C–O, C–S, and N–N bonds,^38^ it is easily available, low cost, and easy to handle.^39^ The solvent-free approach aligns with the principles of green chemistry, as they are eco-friendly and reduce pollution to quite an extent. This method not only enhances laboratory safety by minimizing the potential for solvent-related accidents and exposure to toxic substances but also promotes efficiency. Solvent-free reactions are more efficient as they give more selective and are also high-yielding and cost-effective.^40^

The one-pot, two-step synthesis emerges as a potent and efficient strategy in contemporary organic chemistry, facilitating the streamlined assembly of complex molecules.^41^ This innovative approach involves executing two separate chemical transformations consecutively within a single reaction vessel, eliminating the necessity for intermediate purification and isolation steps.^42^ The advantage of this method lies in its ability to enhance reaction efficiency, reduce waste production, and enhance overall yields. Crucially, it significantly reduces the overall reaction time, underscoring its pivotal role in accelerating synthetic processes.^43^

In continuation of our work in developing green methodology to synthesize biologically important small motifs. A one-pot synthetic route would be a very useful improvement. Herein, we disclose a highly efficient, simple, and environmentally benign, one-pot, tandem method to synthesize 1,2,4-thiadiazoles starting from the corresponding primary amides using Lawesson reagent and *tert*-butyl hydrogen peroxide as an oxidant under metal and solvent-free conditions (Scheme 1).

**Scheme 1:**
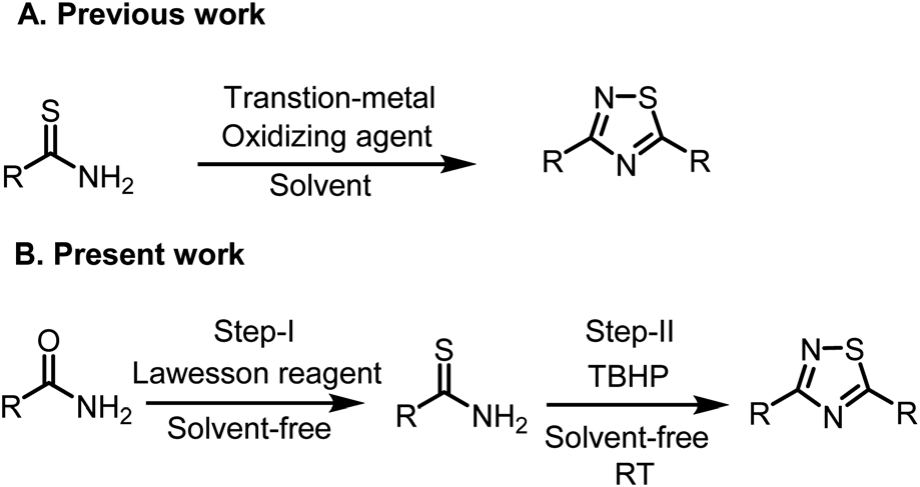
Synthesis of 3,5-disubstituted 1,2,4-thiadiazoles (A) previous work: From thioamides. (B) Present work: from amides.

As far as our knowledge this is the first report of a one-pot, two-step synthesis of 1,2,4-thiadiazoles *via* C–O bond cleavage and new C–S bond formation through a thionation of benzamide and in the second step thioamide, which is generated *in situ* underwent oxidative dimerization to form 1, 2, 4-thiadiazoles.

In order to obtain maximum yield of the symmetrical 3,5-diphenyl-1,2,4-thiadiazole 3a the main efforts were directed towards the best reaction conditions to synthesis 3a. Initially, we selected benzamide 1a, Lawesson reagent and TBHP as model substrates to optimize the reaction conditions for the synthesis of 3a. The effects of different parameters, including reaction medium, molar ratio of oxidant, and temperature, were examined on the model reaction. In search of optimal conditions, first, the reaction was performed with benzamide 1a (1.0 mmol), Lawesson reagent (0.6 mmol), and 1.5 equiv. TBHP was in a one-pot single-step manner in toluene for 1 h at its refluxed temperature but it was unsuccessful (Table 1, entry 1). Then, we moved from a one-step multi-component protocol to a one-pot two-step strategy.

**Table 1:** Optimization of reaction conditions for the synthesis of 3,5-diphenyl-1, 2, 4-thiadiazole 3a from benzamide^a^

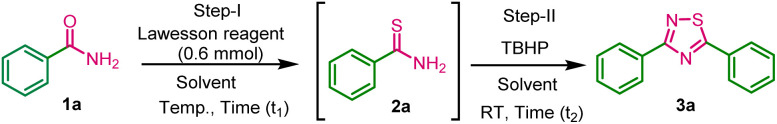						
S·N	Solvent	TBHP (equvi.)	Temp. (^o^C)	Time(*t*_1_) min	Time (*t*_2_) min	Yield 3a^b^ (%)
1^c^	Toluene	1.5	Reflux	60	—	n.r
2	Toluene	1.5	Reflux	60	30	50
3	Benzene	1.5	Reflux	60	30	40
4	THF	1.5	Reflux	60	30	50
5	DCM	1.5	Reflux	60	30	25
6	CH_3_CN	1.5	Reflux	60	30	30
7	EtOH	1.5	Reflux	60	—	n.r
8	MeOH	1.5	Reflux	60	—	n.r
9	Water	1.5	Reflux	60	—	n.r
10	Solvent-free	1.5	60	60	3	65
11	Solvent-free	1.5	80	25	3	92
12	Solvent-free	1.5	100	25	3	93
13	Solvent-free	1.0	80	25	3	70
14	Solvent-free	2.0	80	25	3	92
15	Solvent-free	4.0	80	25	3	91

a Reaction conditions: step 1: 1a (1.0 mmol), Lawesson reagent (0.6 mmol), and solvent (2 mL) at its reflux temp. Step 2: TBHP (1.5 equiv.) at RT (25–30 °C).

b Isolated yield.

c Single-step reaction at reflux temp.

In the first step of this protocol, benzamide 1a (1.0 mmol), and Lawesson reagent (0.6 mmol) were used as model substrates for the preparation of the intermediate thiobenzamide 2a. This reaction mixture was refluxed in toluene for 1 h, and the conversion to the thiobenzamide 2a was monitored by TLC; in the second step, the reaction mixture was allowed to cool down to room temperature, then TBHP was added to it and stirred for 30 min at rt. Gratifyingly, the desired product, 3,5-diphenyl-1,2,4-thiadiazole 3a, was obtained in 50% yield (Table 1, entry 2). Encouraged by this result, we have tested polar aprotic solvents THF, dichloromethane, and acetonitrile under the same reaction conditions giving the product 3a in 30–50% yield (Table 1, entries 4–6). Then we examined polar protic solvents like ethanol, methanol, and water, but very unfortunately, amide 1a did not convert into the thioamide 2a, so we failed to proceed for the second step to give product 3a (Table 1, entries 7–9).

In order to improve the yield of the product, we moved to solvent-free conditions and also to maintain the green chemistry principles in organic synthesis reactions. In the first step of the model reaction, benzamide 1a (1.0 mmol), and Lawesson reagent (0.6 mmol) were heated at 60 °C under solvent-free conditions for 60 min gave intermediate 2a and in the second step, the reaction mixture was allowed to cool at room temperature then TBHP was added to it and stirred for 3 min at rt, gave the desired product 3,5-diphenyl-1,2,4-thiadiazoles 3a in 65% yield (Table 1, entry 10).

Thereafter, the first step of the reaction was carried out at higher temperatures 80°, and 100 °C. Interestingly, the reaction at 80 °C temperature with LR (25 min) and 1.5 equiv. TBHP, under solvent-free conditions, was driven to completion with the desired product 3a to a maximum yield of 92% in total time 3 min (Table 1, entry 11). The high reaction rate may be due to the increased concentration of the reactants in solvent-free conditions. Further, the increase in reaction temperature does not show any considerable change in yield of the product (Table 1, entries 10–12). Next, the different molar ratios of TBHP (1.0, 2.0, 4.0) were also tested, the best result was obtained with 1.5 equiv. of TBHP (Table 1, entries 13–15). Thus, the optimized reaction conditions are primary amide 1a (1.0 mmol), Lawesson reagent (0.6 mmol) at 80 °C, and TBHP (1.5 mmol) at room temperature under solvent-free conditions.

To broaden the scope of this one-pot two-step protocol,a series of different primary aromatic/heteroaromatic and aliphatic amides with distinct functionalities were utilized to synthesize a variety of 1,2,4-thiadiazole. Primary aromatic amide, with electron-donating groups like (methyl, *tert*-butyl, amine, and methoxy) and electron-withdrawing groups as (4-F, Cl, Br), (3-Cl, Br), and (2-Cl, Br) were effectively resulting the desired products in good yields (Scheme 2, 3a–l). Furthermore, the strongly electron-withdrawing groups, such as trifluoromethyl and nitrogroups these substrates also underwent a reaction smoothly and yielded the desired products 3m and 3n in good yields.

**Scheme 2:**
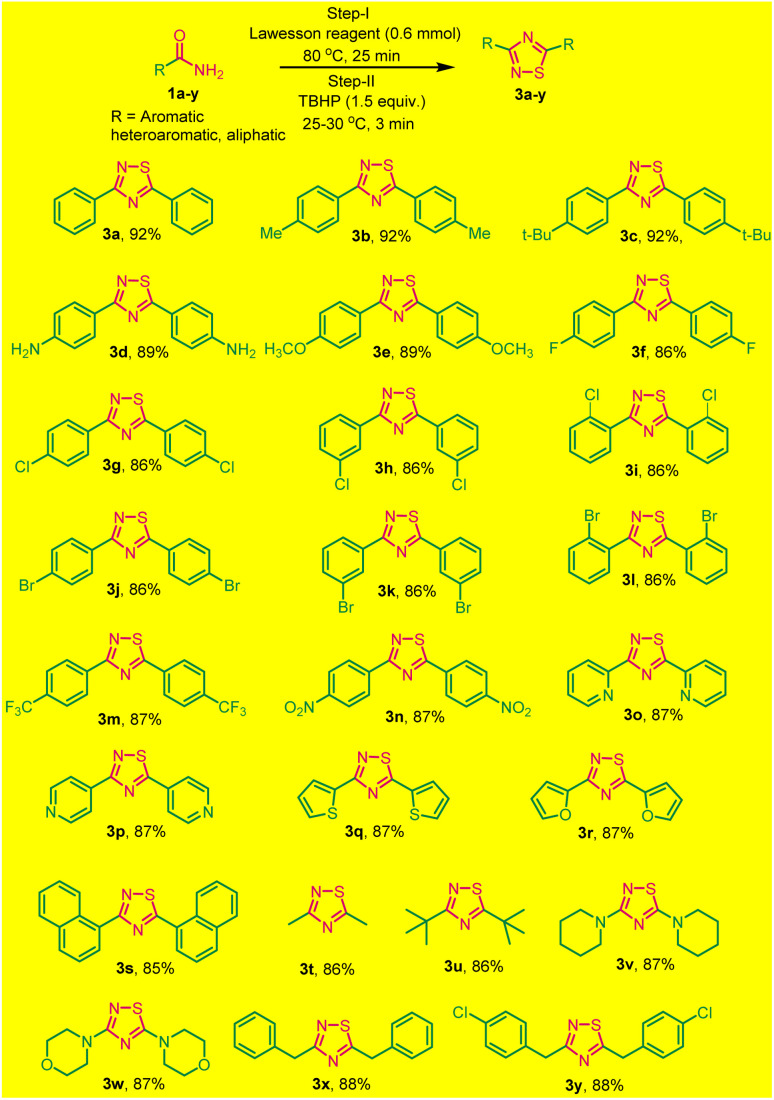
Substrate scope for one-pot two-step synthesis of 1,2,4-thiadiazoles under solvent and catalyst-free (3a–y). ^*a*^Reaction conditions: primary amides (1.0 mmol), Lawesson reagent (0.6 mmol), and TBHP (1.5 equiv.).

Surprisingly, heteroaromatic amides, such as picolinamide, isonicotinamide, thiophene-2-carboxamide and furan-2-carboxamide also participated successfully in this reaction, furnishing 3o–3r in good yields (Scheme 2). Additionally, 1-naphthyl benzamide was subjected to the same conditions and successfully yielded 3,5-(1,1-dinaphthyl)-1,2,4-thiadiazoles 3s with an 85% yield.

To explore the versatility of the reaction, we investigated its compatibility with aliphatic amides, which also underwent smoothly, resulted in the formation of products listed as 3s–3y in good yields (Scheme 2). All the synthesized products were purified without column chromatography by simply recrystallization, that save lots of solvents and our environment to get polluted. The products (3a–3x) were characterized by (^1^H, ^13^C NMR and HRMS spectral data) and confirmed by comparing with those reported. The reaction is reasonably clean, rapid, and efficient. Moreover, the simple experimental and isolation procedure makes it a new efficient route for the synthesis of diverse 3,5-diaryl-1,2,4-thiadiazoles.

After exploring the scope of various 1, 2, 4-thiadiazoles, we attempted to synthesize 3,5-bis(3-pyridyl)-1,2,4-thiadiazole, a promising aromatase inhibitory drug designed to impede the enzymatic conversion of androgen to estrogens within the body, using this developed method. The reaction of nicotinamide 1z (1.0 mmol) with LR (0.6 mmol) and TBHP (1.5 mmol) gave the desired product 3z in 87% yield (Scheme 3).

**Scheme 3:**
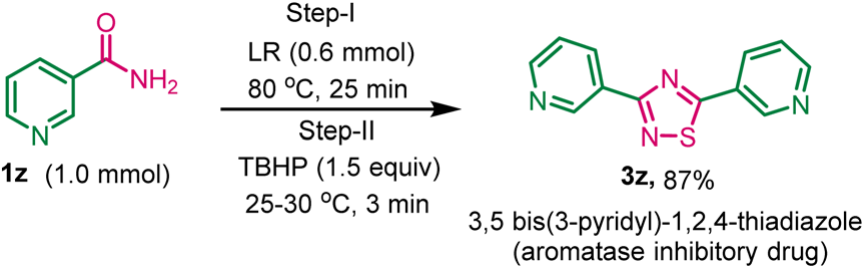
Synthesis of 3,5-bis(3-pyridinyl)-1,2,4-thiadiazole 3z from nicotinamide in solvent-free condition. ^*a*^Reaction conditions: nicotinamide 1z (1.0 mmol), Lawesson reagent (0.6 mmol), and TBHP (1.5 equiv.) in solvent-free condition.

## Gram-scale synthesis of 1,2,4-thiadiazole (3a)

**Scheme 4:**
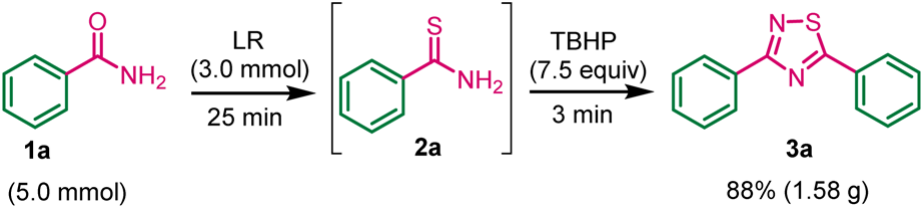
Synthesis of 3a product on gram-scale.

To assess the potential applicability of this methodology in multigram-scale synthesis of 1,2,4-thiadiazole 3a. The experiment was conducted using benzamide 1a (5.0 mmol), LR (3.0 mmol), and TBHP (7.5 mmol) under standard conditions. The product 3a was obtained in 88% yield (Scheme 4). The experimental work showed a robust and acceptable gram-scale synthesis of 1,2,4-thiadiazole.

**Scheme 5:**
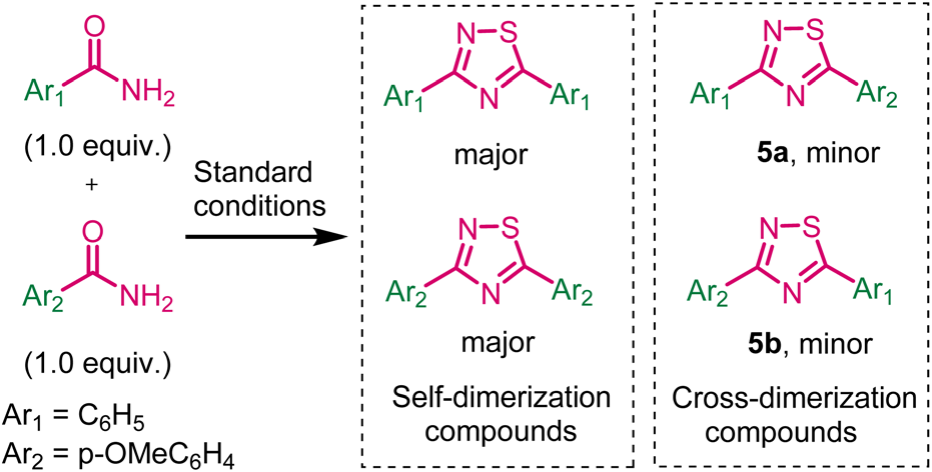
Cross-dimerization reaction.

Next, we have investigated the cross-dimerization reaction by using benzamide and *p*-methoxybenzamide under standard conditions (Scheme 5). Unfortunately, corresponding self-dimerization compounds were obtained as major product (35% yields) and cross-dimerization compounds were obtained as minor products 5a and 5b.

## Control experiment

**Scheme 6:**
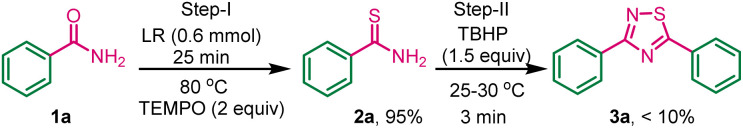
Control experiment with TEMPO.

To investigate the plausible reaction mechanism, a control experiment was carried out between benzamide, Lawesson reagent, and radical scavenger TEMPO ((2,2,6,6-tetramethylpiperidin-1-yl) oxy) (2 equiv.) (Scheme 6), gave 95% yield of 2a, this indicates the thiolation of amide did not quench by TEMPO, so the first step is not a radical path. In the second-step TBHP was added to the above reaction mixture at room temperature. The dimerization process was inhibited by TEMPO which is already present in the reaction mixture and less than 10% of the desired product 3a was obtained, and thiobenzamide-TBHP adduct was isolated (HRMS data, page no. S3, fig. S1†) indicates that the formation of 1,2,4-thiadiazole involved a radical mechanistic pathway in the second step.

## Reaction mechanism

**Scheme 7:**
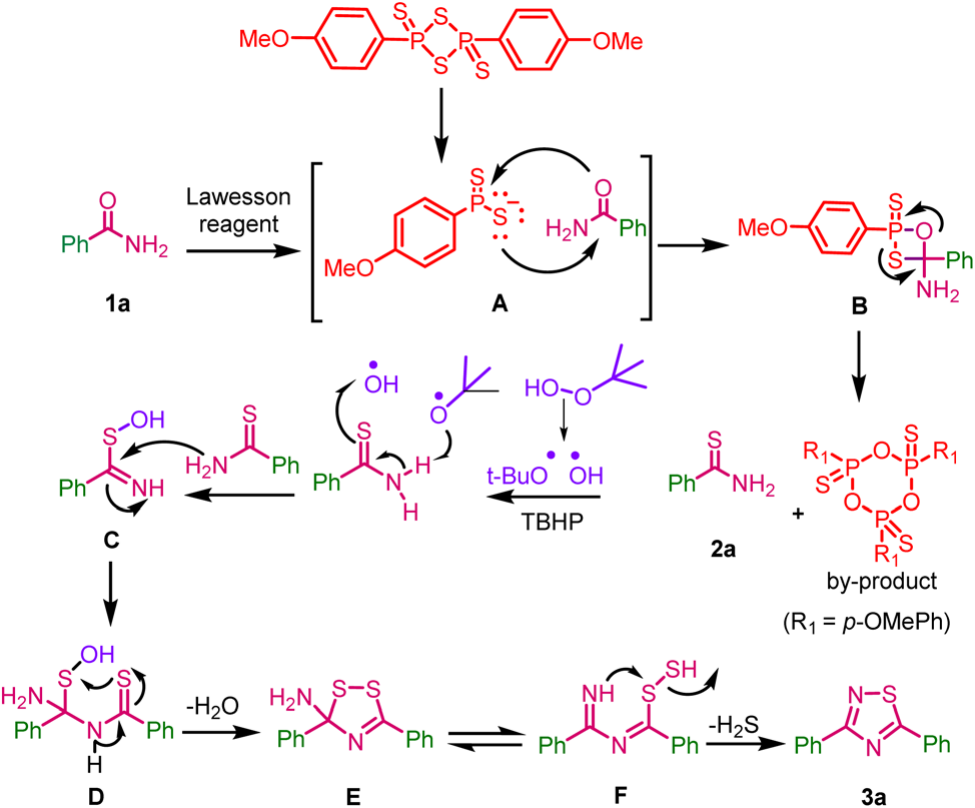
Plausible reaction mechanism for one-pot two-step synthesis of 1,2,4-thiadiazoles.

A plausible reaction mechanism for one-pot, two-step synthesis of 1,2,4-thiadiazoles from primary amides with Lawesson reagent and *tert*-butyl hydrogen peroxide is depicted in (Scheme 7).

In the first step the reactive species dithiophosphine ylide A of Lawesson reagent reacts with benzamide 1a and gives thioxaphosphenate intermediate B; it undergoes cycloreversion to give thiobenzamide 2a along with the by-product 2,4,6-tris(4-methoxyphenyl)-1,3,5,2,4,6-trioxatriphosphinane 2,4,6-trisulfide, which was confirmed by M.P. and HRMS data (ESI, fig. S2†). In the second-step, TBHP undergoes radical dissociation to form *tert*-butoxy and hydroxyl radicals, which may react with thiobenzamide to form intermediate C. This intermediate may undergoes dimerization *via* the elimination of water (H_2_O) through intermediate D to form intermediate E. This may be in equilibrium with intermediate F and it might eliminate hydrogen sulfide (H_2_S) to give the desired product 3a.

## Conclusion

In conclusion, we have demonstrated an efficient and greener protocol for the synthesis of 1,2,4-thiadiazoles using primary amides, Lawesson reagent, and TBHP under solvent-free conditions. This is the first report of a one-pot, two-step synthesis of 1,2,4-thiadiazoles *via* C–O bond cleavage and new C–S bond formation through a thionating reagent (Lawesson reagent) and TBHP. The current protocol found broad substrate scope, excellent functional group tolerance, metal-free conditions, quick conversion, and excellent yields are essential features of this methodology. Since there is no workup and purification of the thioamide intermediate, it saves lots of solvent and time.In addition to its efficiency and simplicity, this study provides a valuable alternative to the prevailing methods available for the synthesis of biologically active 3,5-diaryl/alkyl-1,2,4-thiadiazoles.

## Experimental section

### General information

All solvents and reagents were purchased from commercial sources and used without purification. Pre-coated plates (silica gel 60 F254) from E. Merck were utilized for thin-layer chromatography (TLC). The NMR spectra were recorded on Bruker Avance 500 MHz spectrometers in CDCl_3_/DMSO-*d*_6_ and TMS as an internal standard. All the known products were characterized by proton and carbon NMR and HRMS data were obtained on a Bruker microTOF-QII or Agilent 5975C high resolution mass spectrometers.

### General procedure for the synthesis of 3,5-disubstituted-1,2,4-thiadiazoles

An oven-dried round bottom flask (25 mL) equipped with a stir bar was charged with primary amide (1.0 mmol) and Lawesson reagent (0.6 mmol); the reaction mixture was heated at 80 °C, and the progress of the reaction was monitored by TLC. After completion of the reaction (25 min) it was cooled to room temperature, and TBHP (1.5 equiv.) was added to the mixture and stirred at room temperature further; the progress of the reaction was monitored by TLC. After completion of the reaction, the solid mixture was extracted with ethyl acetate and washed with water. The organic layer was dried over Na_2_SO_3,_ and the solvent was removed under reduced pressure. The product was purified by recrystallization with ethanol to obtain the desired 1,2,4-thiadiazoles. All the products were characterized by ^1^H, ^13^C-NMR, and HRMS.

## Data availability

All data are available in ESI.†

## Conflicts of interest

The authors declare no conflict of interest.

## Supplementary Material

RA-014-D4RA03993A-s001
