# Supplementary material for: A chromatography-free one-pot, two-step synthesis of 1,2,4-thiadiazoles from primary amides via thiolation and oxidative dimerization under solvent-free conditions: a greener approach
Source: RSC Adv. 2024 Jul 16;14(31):22480–5. doi: 10.1039/d4ra03993a (PMC11250125; doi:10.1039/d4ra03993a)

Electronic Supplementary Material (ESI) for RSC Advances.
This journal is © The Royal Society of Chemistry 2024

Supporting Information

A Chromatography-Free One-Pot, Two-Step Synthesis of 1,2,4-Thiadiazoles from Primary Amides via Thiolation and Oxidative Dimerization under Solvent-Free Conditions: A Greener Approach

List of contents page no

1	General information	S2
2	General experimental procedure for the synthesis of compounds**3a-3z**	S2
3	Mechanistic study:Radical trapping experiment by TEMPO	S3
4	Analytical data of products	S5
4.1	^{1}H, ^{13}C, ^{19}F & HRMS spectral data	S5
4.2	^{1}H, ^{13}C,^{19}F & HRMS spectra	S12

1. General Information

All solvents and reagents were purchased from commercial sources and used without purification. Pre-coated plates (silica gel 60 F254) from E. Merck were utilized for thin-layer chromatography (TLC). The NMR spectra were recorded on Bruker Avance 500 MHz spectrometers in $CDCl_3$/DMSO-d_6 and TMS as an internal standard. All the known products were characterized by proton and carbon NMR andHRMS data was obtained on a Bruker microTOF-QII or Agilent 5975C high resolution mass spectrometers.

2.Experimental procedure

2.1 General procedure for the synthesis of 1,2,4-thiadiazoles

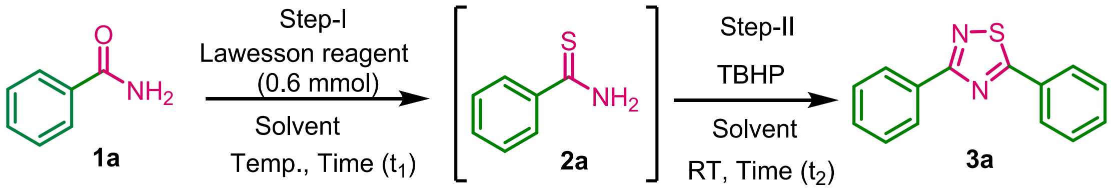

An oven-dried round bottom flask (25mL) equipped with a stir bar was charged with primary amide (1.0 mmol) and Lawesson reagent (0.6 mmol) the reaction mixture was heated at 80 °C and the progress of the reaction was monitored by TLC. After completion of the reaction (25 min) it was cooled to room temperature and TBHP (1.5 equivalent) was added to the mixture and stirred at room temperature further the progress of the reaction was monitored by TLC. After completion of the reaction the solid mixture was dissolved in ethyl acetate and the undissolve solid was discarded and the ethyl acetate portion was washed with water. Organic layer was dried over Na_2SO_3 and the solvent was removed under reduced pressure. The product was purified by recrystallization with ethanol to obtained the desired thiadiazoles 3a. All the products were characterized based on ^{1}H-NMR, ^{13}C-NMR and HR-MS.

3. Mechanistic Studie;Radical trapping experiment by TEMPO

Several mechanistic investigations were performed to investigate the mechanism. At first, 2, 2,6,6-tetramethylpiperidinooxy (TEMPO) radical scavengers(2 equiv.) was added to the reaction system, and the trace amount of the **3a** was formed, and TEMPO adducts **4a** were detected in HRMS data from the crude reaction mixture (Figure S1). These results suggested that the reaction passes through the radical pathway.

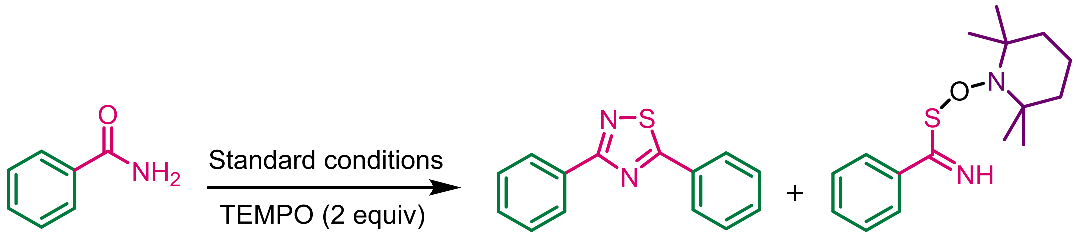

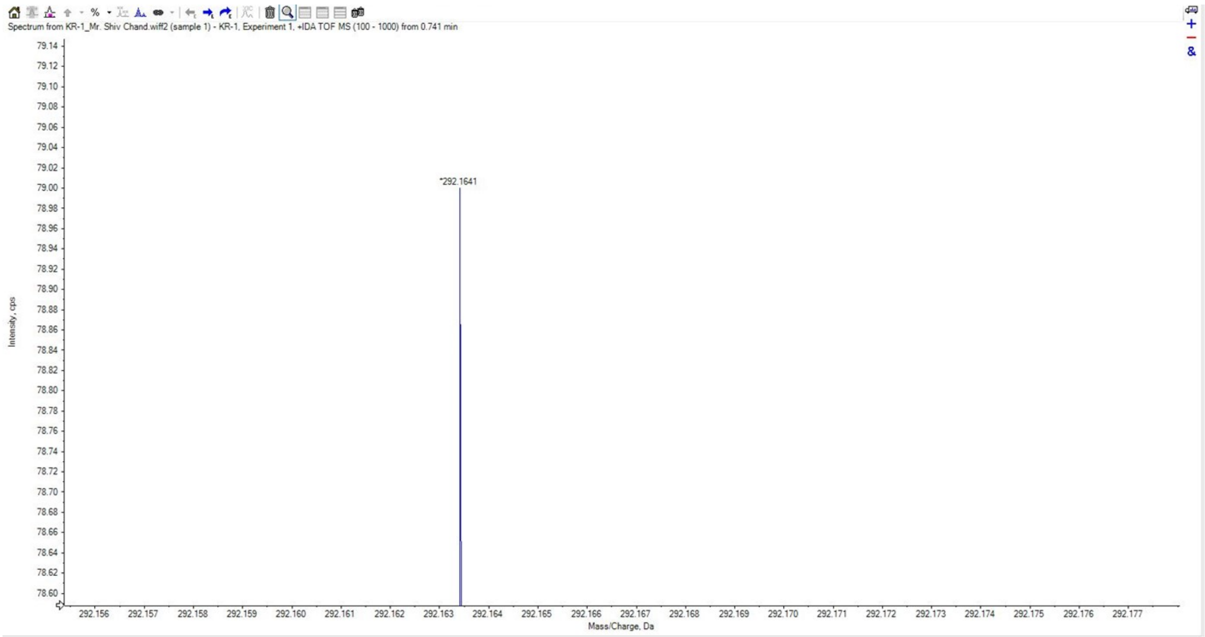

Figure S1. HRMS of adduct **4a**

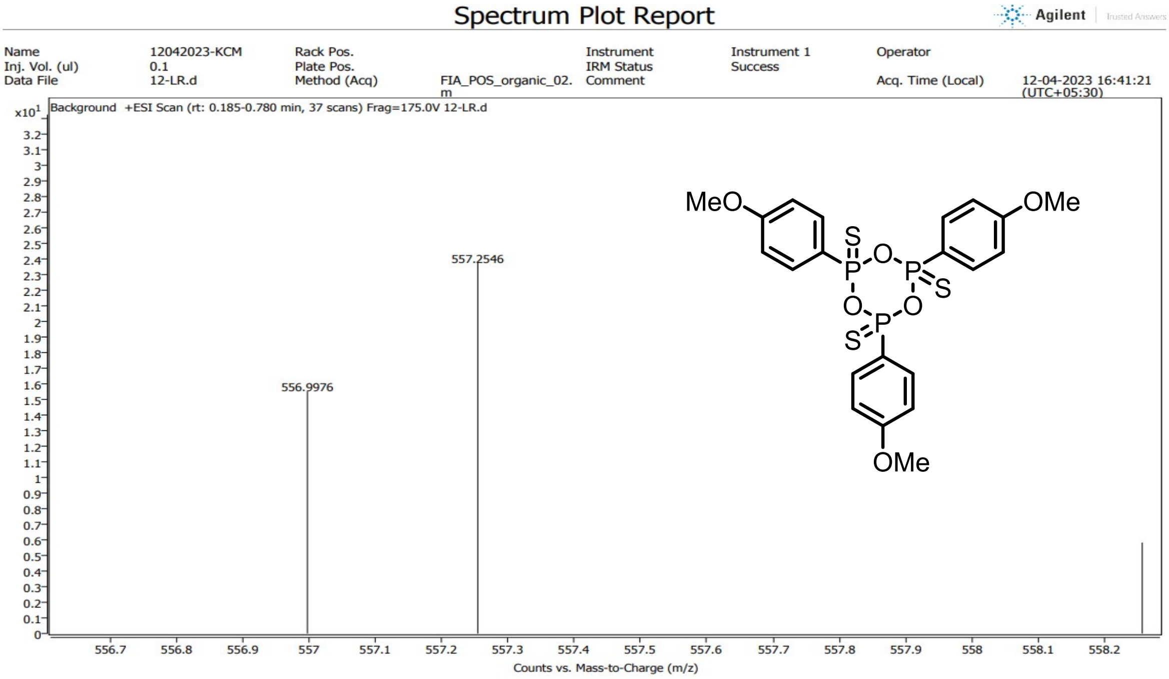

Figure S2. HRMS data of by-product

4. Analytical data of products

[4.1] ^{1}H,^{13}C, ^{19}F & HRMS spectral data

[4.1.1] 3,5-diaryl-1,2,4-thiadiazoles (3a)

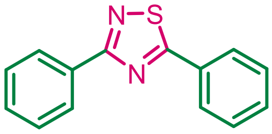

Yield: 92%; white solid; m.p. 91 °C; **^{1}H NMR** (500 MHz, $CDCl_3$) δ 8.43 (dd, *J*= 7.9, 1.7 Hz, 2H), 8.09 (dd, *J* = 7.8, 1.7 Hz, 2H), 7.59-7.49 (m, 6H) ppm. **^{13}C NMR** (126 MHz, $CDCl_3$) δ 188.2, 173.8, 132.9, 131.9, 130.7, 130.4, 129.3, 128.7, 4 128.4, 127.5 ppm. HRMS (ESI) calculated for $C_{14}H_{10}N_2S$ [M+H$^+$]: 239.0643, found 239.0668.

[4.1.2] 3,5-dip-tolyl-1,2,4-thiadiazole (3b)

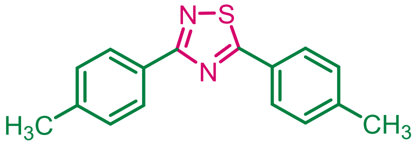

Yield: 92%; white solid; m.p. 134-136 °C; **^{1}H NMR** (500 MHz, $CDCl_3$) δ 8.28 (d, *J* = 8.1 Hz, 2H), 7.93 (d, *J* = 8.1 Hz, 2H), 7.31 (t, *J* = 7.6 Hz, 4H), 2.44 (d, *J* = 5.0 Hz, 6H) ppm.**^{13}C NMR** (126 MHz, $CDCl_3$) δ 188.1, 173.9, 142.6, 140.6, 130.5, 130.0, 129.5, 128.4, 128.3, 127.6, 21.7, 21.6 ppm. HRMS: (ESI) calculated forC$_{16}$H$_{14}$N$_2$S[M+H$^+$]: 267.0956, found 267.0953.

[4.1.3]3,5-bis(4-tert-butylphenyl)-1,2,4-thiadiazole (3c)

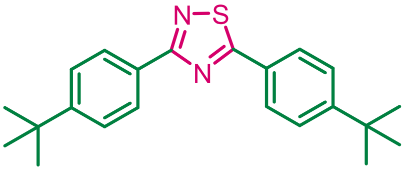

Yield 92%; white solid; m.p. 91 °C; **^{1}H NMR** (500 MHz, $CDCl_3$) δ 8.32 (d, *J* = 8.4 Hz, 2H), 7.99 (d, *J* = 8.3 Hz, 2H), 7.57-7.54 (d, *J* = 8.4 Hz, 4H), 1.40 (s, 17H) ppm. **^{13}C NMR** (126 MHz, $CDCl_3$) δ 188.0, 173.9, 155.7, 153.7, 130.5, 128.3, 128.3, 127.4, 126.3, 125.8, 35.2, 35.0, 31.4, 31.2 ppm. HRMS (ESI) calculated $C_{22}H_{26}N_2S$ [M+H$^+$]: 351.1895, found 351.1904.

[4.1.4] 3,5-bis(4-aminophenyl)-1,2,4-thiadiazole (3d)

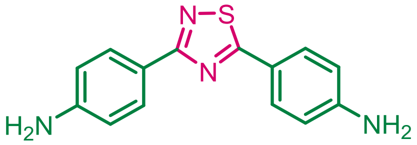

Yield 89%; orange-yellow solid; m.p. 208-209 °C; **^{1}H NMR** (500 MHz, $CDCl_3$) δ 8.20 (d, *J* = 8.7 Hz, 1H), 7.87-7.82 (d, *J* = 8.7 Hz, 1H), 7.45-7.42 (d, *J* = 8.7 Hz, 4H), 4.17 (br s, *J* = 8.7 Hz, 4H) ppm. **^{13}C NMR** (126 MHz, $CDCl_3$) δ 150.4, 133.8, 129.8, 129.3,

129.2, 120.0, 114.7, 114.7, 114.4, 113.7, 100.3 ppm. HRMS (ESI) calculated $C_{14}H_{12}N_4S$ [M+H$^+$]: 269.0781, found 269.0790.

[4.1.5]3,5-bis(4-methoxyphenyl)-1,2,4-thiadiazole (3e)

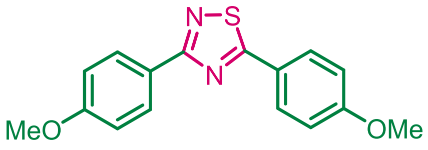

Yield: 89%; white solid; m.p. 139-141 °C; **^{1}H NMR** (500 MHz, $CDCl_3$) δ 8.33 (d, *J* = 8.4 Hz, 2H), 7.99 (d, *J* = 8.4 Hz, 2H), 7.01 (d, *J* = 8.4 Hz, 4H), 3.89 (s, 6H) ppm. **^{13}C NMR** (126 MHz, $CDCl_3$) δ 187.3, 173.4, 162.5, 161.3, 129.9, 129.2, 126.0, 123.7, 114.6, 113.9, 55.5, 55.3 ppm. HRMS (ESI) calculated for $C_{16}H_{14}N_2O_2S$ [M+H$^+$]: 299.0854, found 299.0823.

[4.1.6]3,5-bis(4-fluorophenyl)-1,2,4-thiadiazole (3f)

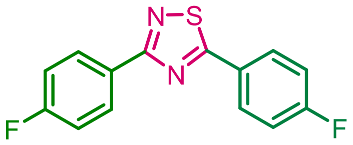

Yield 85%; white solid; m.p. 184-187 °C; **^{1}H NMR** (500 MHz, $CDCl_3$) δ 8.39 (dd, *J* = 8.9, 5.5 Hz, 2H), 8.04 (dd, *J* = 8.9, 5.2 Hz, 2H), 7.23-7.16 (m, 4H) ppm. **^{13}C NMR** (126 MHz, $CDCl_3$) δ 187.1, 172.9, 166.0 (d, *J* = 253.2 Hz), 165.3 (d, *J* = 250.1 Hz), 130.6 (d, *J* = 8.2 Hz), 129.8 (d, *J* = 8.8 Hz), 129.2 (d, *J* = 3.7 Hz), 127.1 (d, *J* = 2.5 Hz), 116.7 (d, *J* = 22.6 Hz), 115.9 (d, *J* = 21.4 Hz) ppm. HRMS: (ESI) calculated for $C_{14}H_8F_2N_2S$ [M+H$^+$]: 275.0455, found 275.0455.

[4.1.7]3,5-bis(4-chlorophenyl)-1,2,4-thiadiazole (3g)

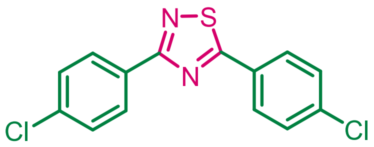

Yield 86%; white solid; m.p. 161-163 °C; **^{1}H NMR** (500 MHz, $CDCl_3$) δ 8.35 (d, *J* = 8.6 Hz, 2H), 8.00 (d, *J* = 8.6 Hz, 2H), 7.59-7.49 (dd, *J* = 16.4, 8.6 Hz, 4H) ppm. **^{13}C NMR** (126 MHz, $CDCl_3$) δ 187.6, 172.7, 138.3, 136.8, 131.3, 129.8, 129.9, 128.2, 129.1, 128.8 ppm. HRMS: (ESI) calculated for $C_{14}H_8Cl_2N_2S$ [M+H$^+$]: 306.9863, found 306.9865.

[4.1.8]3,5-bis(3-chlorophenyl)-1,2,4-thiadiazole (3h)

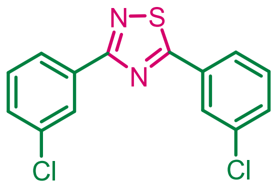

Yield 86%; white solid, m.p. 125-127 °C; **^{1}H NMR** (500MHz, $CDCl_3$): δ 8.30 (d, *J* = 1.8 Hz, 1H), 8.16 (dd, *J* = 7.0 Hz, 1H), 8.05 (d, *J* = 1.9 Hz, 1H), 7.69 (d, *J* = 7.5 Hz, 1H), 7.51-7.50 (m, 4H) ppm. **^{13}C NMR** (126 MHz, $CDCl_3$) δ 186.9, 172.9, 134.2, 134.1, 132.2, 131.1, 131.0, 130.2, 130.0, 129.9, 128.1,127.2, 126.4 ppm. HRMS (ESI) calculated for [M+H$^+$]: 306.9795 $C_{14}H_8Cl_2N_2S$ found 306.9790.

[4.1.9]3,5-bis(2-chlorophenyl)-1,2,4-thiadiazole (3i)

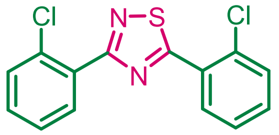

Yield 86%; white solid, m.p. 91-93 °C; **^{1}H NMR** (500MHz, $CDCl_3$): δ 8.67 (d, *J* = 1.8 Hz, 1H), 8.08 (dd, *J* = 7.0 Hz, 1H), 7.99-7.55 (m, 2H), 7.48-7.41 (m, 4H) ppm. **^{13}C NMR** (126 MHz, $CDCl_3$) δ 184.5, 170.7, 134.1, 134.0, 132.2, 132.2, 131.7, 131.5, 130.9, 127.9, 127.6 127.4, 123.5, 122.2 ppm. HRMS (ESI) calculated for [M+H$^+$]: 306.9795 $C_{14}H_8Cl_2N_2S$ found 306.9791.

[4.1.10] 3,5-bis(4-bromophenyl)-1,2,4-thiadiazole (3j)

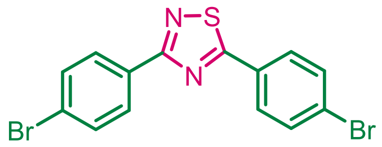

Yield 86%; white solid; m.p. 150-151 °C; **^{1}H NMR** (500 MHz, $CDCl_3$) δ 8.35-8.33 (d, *J* = 7.8 Hz, 1H), 8.01-8.00 (d, *J* = 7.6 Hz, 1H), 7.54-7.49 (m, 4H) ppm. **^{13}C NMR** (126 MHz, $CDCl_3$) δ 187.9, 172.9, 136.6, 131.9, 129.7, 129.6, 129.0, 128.9, 128.8, 128.7, ppm. HRMS (ESI) calculated for 394.8775, [M+H$^+$]: $C_{14}H_8Br_2N_2S$ found 394.8780.

[4.1.11] 3,5-bis(3-bromophenyl)-1,2,4-thiadiazole (3k)

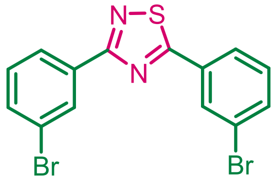

Yield 86%; white solid; m.p. 113-114 °C; **^{1}H NMR** (500 MHz, $CDCl_3$) δ 9.25 (d, *J* = 7.8 Hz, 1H), 8.99 (d, *J* = 7.6 Hz, 1H), 8.53 (d, *J* = 7.5 Hz, 1H), 8.09 (m, 1H), 7.99 (m, 2H), 7.66-7.65 (m, 2H) ppm. **^{13}C NMR** (126 MHz, $CDCl_3$) δ 184.5, 170.6, 134.1, 134.0, 133.2, 132.2, 131.7, 131.5, 1309, 127.9, 127.6, 127.4, 123.4, 122.2 ppm. HRMS (ESI) calculated for 394.8774, [M+H$^+$]:

$C_{14}H_8Br_2N_2S$ found 394.8771.

[4.1.12] 3,5-bis(2-bromophenyl)-1,2,4-thiadiazole (3l)

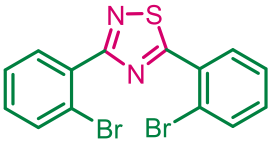

Yield 86%; white solid; m.p. 97 °C; **^{1}H NMR** (500 MHz, $CDCl_3$) δ 8.65 (d, J = 7.8 Hz, 1H), 8.00 (d, J = 7.6 Hz, 1H), 7.80 (d, J = 7.5 Hz, 2H), 7.55-7.52 (m, 2H), 7.37-7.34 (m, 2H) ppm. **^{13}C NMR** (126 MHz, $CDCl_3$) δ 184.6, 170.8, 134.1, 134.0, 134.0, 132.2, 132.2, 131.7, 1315, 130.9, 127.9, 127.4, 123.4, 122.2 ppm. HRMS (ESI) calculated for 394.8858, [M+H$^+$]: $C_{14}H_8Br_2N_2S$ found 394.8853.

[4.1.13] 3,5-bis(trifluoromethyl)phenyl-1,2,4-thiadiazole (3m)

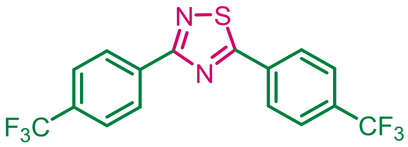

Yield 85%; white solid; m.p. 80 °C; **^{1}H NMR** (500 MHz, $CDCl_3$) δ 8.55 (d, J = 8.1 Hz, 2H), 8.21 (d, J = 8.1 Hz, 2H), 7.84-7.79 (dd, J = 17.5, 8.1 Hz, 4H) ppm. **^{13}C NMR** (126 MHz, $CDCl_3$) δ 186.9, 172.7, 135.6, 133.8 (q, J = 32.7 Hz), 128.6 (q, J = 32.4 Hz), 128.6, 127.9, 127.8, 126.4 (q, J = 3.7 Hz), 125.7 (q, J = 3.7 Hz), 124.6 (q, J = 270.7 Hz), 122.8 (q, J = 270.9 Hz) ppm. HRMS: (ESI) calculated for $C_{16}H_8F_6N_2S$ [M+H$^+$]: 375.0391, found 375.0412.

[4.1.14] 3,5-bis(4-nitrophenyl)-1,2,4-thiadiazole (3n)

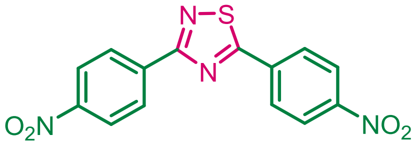

Yield 85%; pale yellow solid; m.p. 200-202 °C; **^{1}H NMR** (500 MHz, $CDCl_3$) δ 8.58 (d, J = 8.2 Hz, 2H), 8.45-8.37 (m, 4H), 8.26-8.24 (d, J = 8.2 Hz, 2H) ppm. **^{13}C NMR** (126 MHz, $CDCl_3$) δ 186.8, 172.4, 149.8, 148.9, 137.9, 135.9, 129.5, 128.6, 124.9, 124.3 ppm. HRMS (ESI) calculated for $C_{14}H_8N_4O_4S$ [M+H$^+$]: 329.0270, found 329.0274.

[4.1.15] 3,5-bis(2-pyridinyl)-1,2,4-thiadiazole (3o)

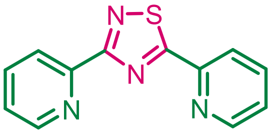

Yield 83%; yellow solid; m.p. 133 °C; **^{1}H NMR** (500 MHz, $CDCl_3$) δ 7.91 (d, J = 8.0 Hz, 2H), 7.75 (d, J = 4.1 Hz, 2H), 7.69 (d, J = 1.7 Hz, 2H), 7.57-7.55 (m, 2H) ppm. **^{13}C NMR** (126 MHz, $CDCl_3$) δ 185.6, 166.3,

151.3, 149.7, 144.1, 143.2, 140.5, 140.1 128.4, 126.7, 124.2, 123.7, 96.2 ppm. HRMS: (ESI) calculated for $C_{12}H_8N_4S$ [M+H$^+$]: 241.0548, found 241.0556.

[4.1.16] 3,5-bis(4-pyridinyl)-1,2,4-thiadiazole (3p)

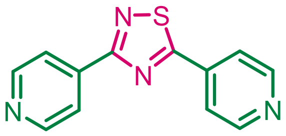

Yield 83%; white solid; m.p. 195-196 °C; **^{1}H NMR** (500 MHz, $CDCl_3$) δ 8.93-8.88 (m, 4H), 8.35-8.33 (m, 2H), 8.02-8.00 (m, 2H) ppm. **^{13}C NMR** (126 MHz, $CDCl_3$) δ 186.7, 172.1, 151.3, 150.3, 139.9, 136.8, 122.2, 120.9 ppm. HRMS: (ESI) calculated for $C_{12}H_8N_4S$ [M+H$^+$]: 241.0470, found 241.0475.

[4.1.17] 3,5-di-thiophen-2-yl-[1,2,4]thiadiazole (3q)

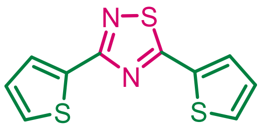

Yield 84%; white solid; m.p. 121 °C; **^{1}H NMR** (500 MHz, $CDCl_3$) δ 9.25 (d, J = 8.6 Hz, 1H), 8.99 (d, J = 8.5 Hz, 1H), 8.53 (dd, J = 7.2, 0.9 Hz, 1H), 8.11-7.98 (m, Hz, 5H), 7.71-7.59 (m, 6H) ppm. **^{13}C NMR** (126 MHz, $CDCl_3$) δ 180.6, 168.4, 136.1, 133.1, 130.5, 129.9, 129.3, 129.8, 128.5, 127.9 ppm. HRMS: (ESI) calculated for $C_{10}H_6N_2S_3$ [M+H$^+$]: 250.9699, found 250.09710.

[4.1.18] 3,5-di-furan-2-yl-[1,2,4]thiadiazole (3r)

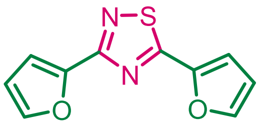

Yield 84%; white solid; m.p. 61-62 °C; **^{1}H NMR** (500 MHz, $CDCl_3$) δ 7.44 (d, J = 8.6 Hz, 1H), 7.23-7.22 (d, J = 8.5 Hz, 1H), 6.52 (dd, J = 7.2, 0.9 Hz, 1H), 6.35-6.30 (m, Hz, 1H), 6.28 (m, 1H), 6.22-6.20 (m, 1H) ppm. **^{13}C NMR** (126 MHz, $CDCl_3$) δ 177.1, 165.0, 148.0, 146.6, 145.9, 144.6, 112.9, 111.8 ppm. HRMS: (ESI) calculated for $C_{10}H_6N_2S_3$ [M+H$^+$]: 219.0150, found 219.0154.

[4.1.19] 3,5-di-(naphthalalen-1-yl)-1,2,4-thiadiazole (3s)

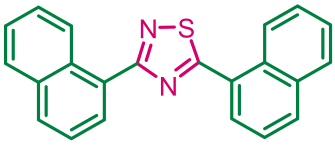

Yield 85%; white solid; m.p. 121 °C; **^{1}H NMR (500 MHz, $CDCl_3$)** δ 9.25 (d, J = 8.6 Hz, 1H), 8.99 (d, J = 8.5 Hz, 1H), 8.53 (dd, J = 7.2, 0.9 Hz, 1H), 8.11-7.98 (m, Hz, 5H), 7.71-7.59 (m, 6H), ppm. **^{13}C NMR** (126

MHz, $CDCl_3$) δ 186.9, 173.9, 134.2, 134.1, 132.2, 131.2, 131.0, 130.2, 129.9, 129.3, 128.7, 128.6, 128.0, 127.8, 127.3, 126.8, 126.4, 126.1, 125.4, 125.2, 125.2 ppm. HRMS:(ESI) calculated for $C_{22}H_{14}N_2S$ [$M+H^+$]: 339.0956, found 339.0965.

[4.1.20] 3,5-dimethyl-1,2,4-thiadiazole (3t)

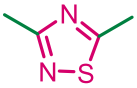

Yield 86%; yellow oil; **^{1}H NMR** (500 MHz, $CDCl_3$) δ 2.47 (s, 3H), 2.60 (s,3H), ppm. **^{13}C NMR** (126 MHz, $CDCl_3$) δ 189.2, 174.2, 18.5, 16.6 ppm. HRMS: (ESI) calculated for $C_4H_6N_2S$ [$M+H^+$]: 115.0258, found 115.0260.

[4.1.21] 3,5-di-tert-butyl-1,2,4-thiadiazole (3u)

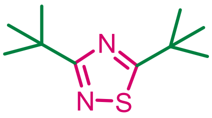

Yield 86%; yellow oil; **^{1}H NMR** (500 MHz, $CDCl_3$) δ 1.57(m, 9H), 1.48 (m, 9H), ppm. **^{13}C NMR** (126 MHz, $CDCl_3$) δ 188.0, 173.9, 37.8, 35.2, 31.4, 28.2 ppm. HRMS: (ESI) calculated for $C_4H_6N_2S$ [$M+H^+$]: 199.1191, found 199.1195.

[4.1.22]3,5-di-piperidine-1-yl-[1,2,4]thiadiazole (3v)

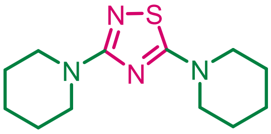

Yield 87%; yellow solid; m.p. 121 °C; **^{1}H NMR** (500 MHz, $CDCl_3$) δ 3.87 (d, *J* = 5.2 Hz, 4H), 3.65 (d, *J* = 5.1 Hz, 4H), 1.51 (m, 6H), 1.28 (m, 6H) ppm. **^{13}C NMR** (126 MHz, $CDCl_3$) δ 184.0, 168.4, 48.2, 46.8, 25.9, 25.3, 24.5 24.0 ppm. HRMS: (ESI) calculated for $C_{12}H_8N_4S$ [$M+H^+$]: 253.1409, found 253.1404.

[4.1.23]3,5-di-morpholine-4-yl-1,2,4]thiadiazole (3w)

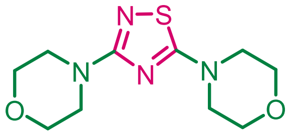

Yield 87%; yellow solid; m.p. 133 °C; **^{1}H NMR** (500 MHz, $CDCl_3$) δ 3.79-3.76 (m, 8H), 3.63-3.60 (m, 2H), 3.54-3.50 (m, 4H), 3.49-3.32 (m, 2H) ppm. **^{13}C NMR** (126 MHz, $CDCl_3$) δ 184.0, 168.4, 66.2, 66.0, 48.2, 46.8, ppm. HRMS: (ESI) calculated for $C_{12}H_8N_4S$ [$M+H^+$]: 281.0994, found 281.0998.

[4.1.24] 3,5-di-benzyl-1,2,4-thiadiazole (3x)

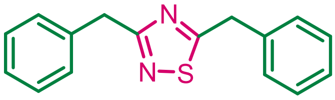

Yield 88%; yellow oil; ^{1}**H NMR** (500 MHz, $CDCl_3$) δ 8.50 (m, 2H), 7.68-7.67 (m, 1H), 7.56-7.54 (m, 2H), 7.41 (m, 2H), 7.36 (m, 2H), 7.28 (m, 1H1), 4.47 (s, 4H), ppm. ^{13}**C NMR** (126 MHz, $CDCl_3$) δ 187.3, 176.9, 136.9, 134.8, 134.3, 131.3, 129.2, 128.9, 128.8, 127.1, 39.6, 37.2 ppm. HRMS: (ESI) calculated for $C_{16}H_{14}N_2S$ [M+H$^+$]: 267.0956, found 267.0957.

[4.1.25] 3,5-bis(4-chloro-benzyl)-[1,2,4]thiadiazole (3y)

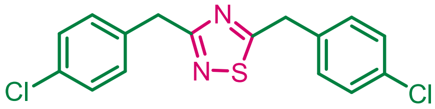

Yield 88%; yellow oil; ^{1}**H NMR** (500 MHz, $CDCl_3$) δ 7.70-7.67 (m, 2H), 7.55-7.53 (m, 2H), 7.41-7.40 (m, 2H), 7.36-7.35 (m, 2H), 4.47 (s, 4H), ppm. ^{13}**C NMR** (126 MHz, $CDCl_3$) δ 187.2, 181.8, 176.5, 141.8, 135.2, 133.2, 132.7, 132.5, 130.6, 129.4, 129.0, 38.9, 29.8 ppm. HRMS: (ESI) calculated for $C_{16}H_{14}N_2S$ [M+H$^+$]: 335.0098, found 335.0092.

[4.1.26] 3,5-bis(3-pyridinyl)-1,2,4-thiadiazole (3z)

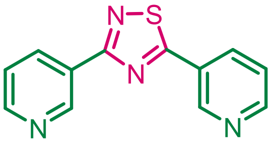

Yield 85%; white Solid; m.p. 132-135 ^{o}C; ^{1}**H NMR** (500 MHz, $CDCl_3$): δ 9.63 (d, 1H), 9.29 (d, 1H), 9.05 (d, 1H), 8.83 (m, 1H), 8.76-8.73 (m, 1H), 8.68-8.67 (m, 1H), 7.55-7.52 (m, 1H), 7.50-7.47 (m, 1H) ppm. ^{13}**C NMR** (126 MHz, $CDCl_3$) δ 185.6, 171.6, 152.8, 151.3, 149.7, 148.5, 135.6, 134.6, 128.4, 126.7, 124.2, 123.7 ppm. HRMS: (ESI) calculated for $C_{12}H_8N_4S$ [M+H$^+$]: 241.0548, found 241.0556.

[4.2] ^{1}H, ^{13}C, ^{19}F & HRMS Spectra

[4.2.1] 3,5- diaryl-1,2,4-thiadiazole in $CDCl_3$ (3a)

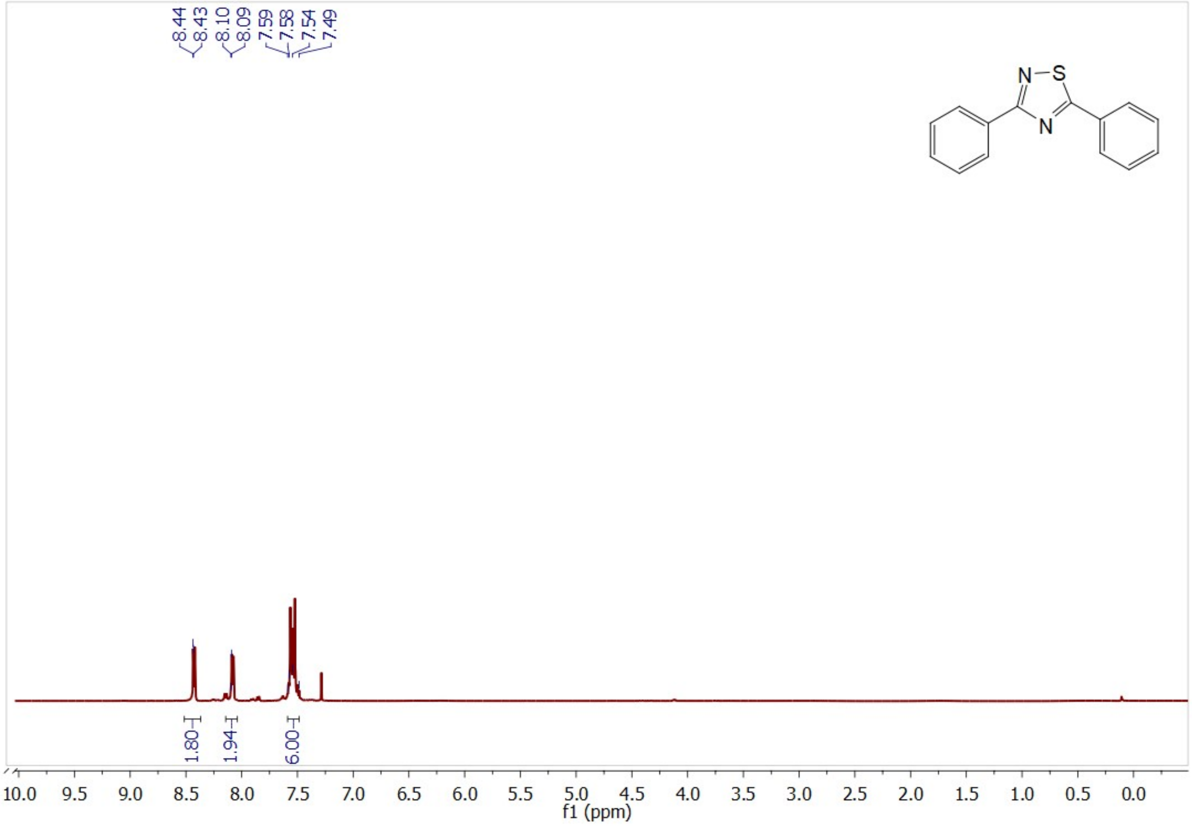

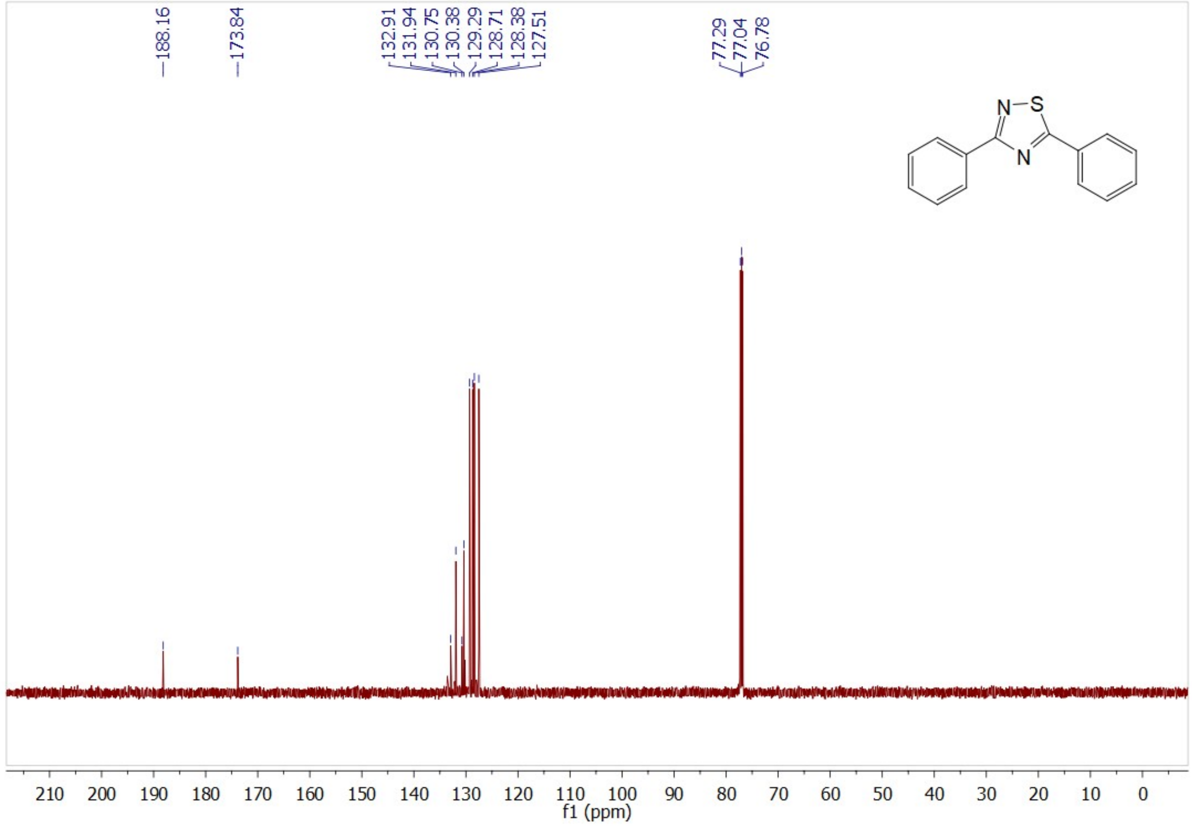

Mass spectrum of 3a

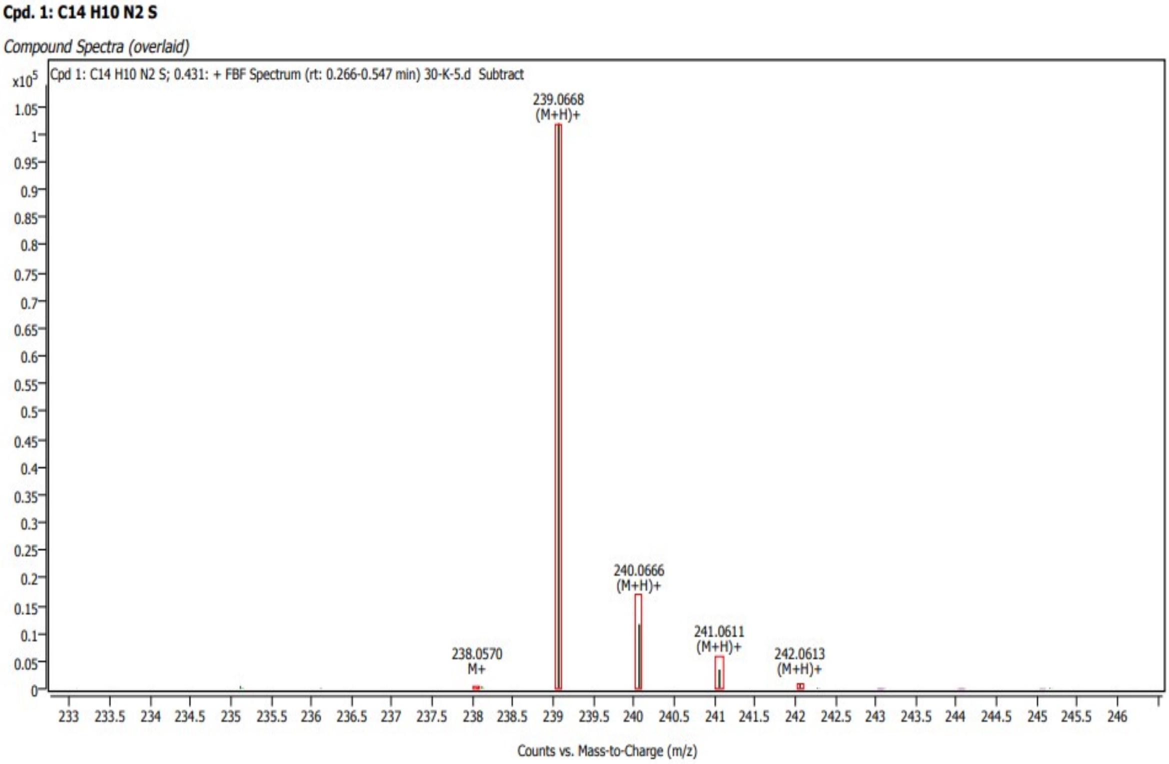

[4.2.5] 3,5-dip-tolyl-1,2,4-thiadiazole in $CDCl_3$ (3b)

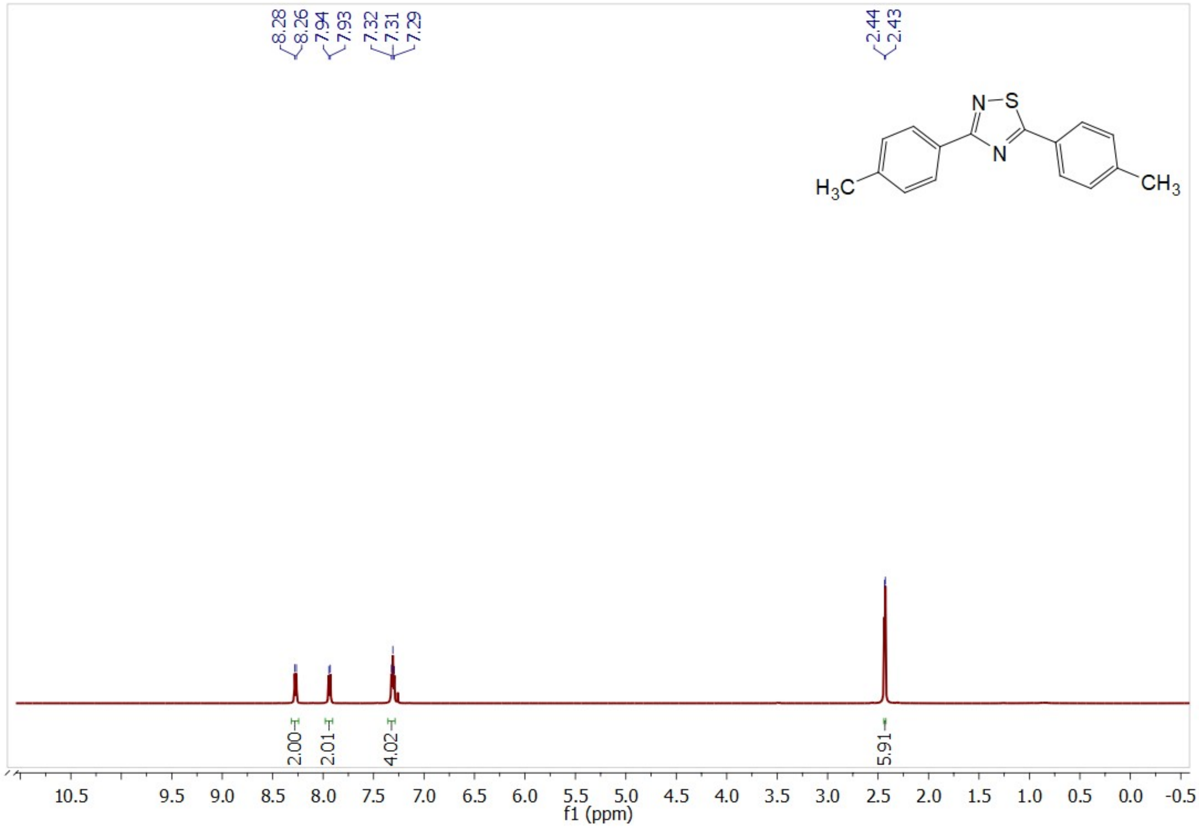

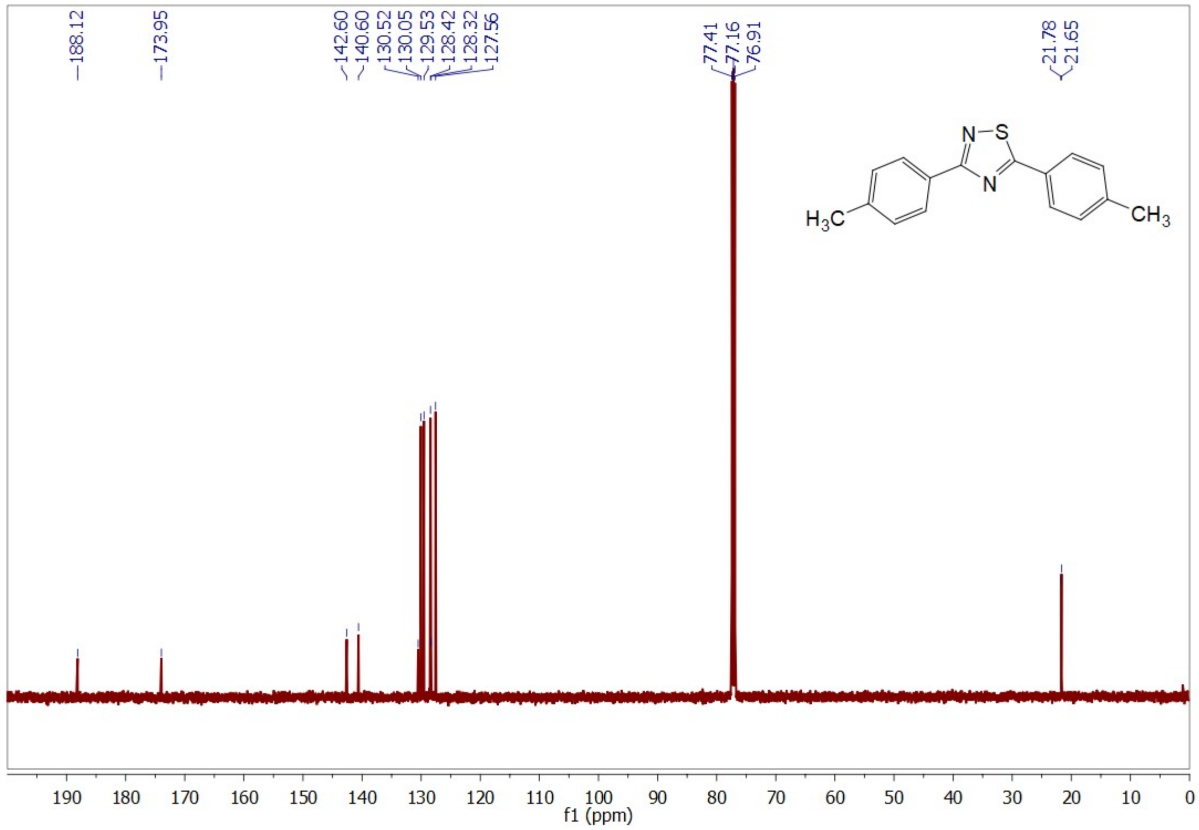

[4.2.3] 3,5-bis(4-tert-butylphenyl)-1,2,4-thiadiazole in $CDCl_3$ (3c)

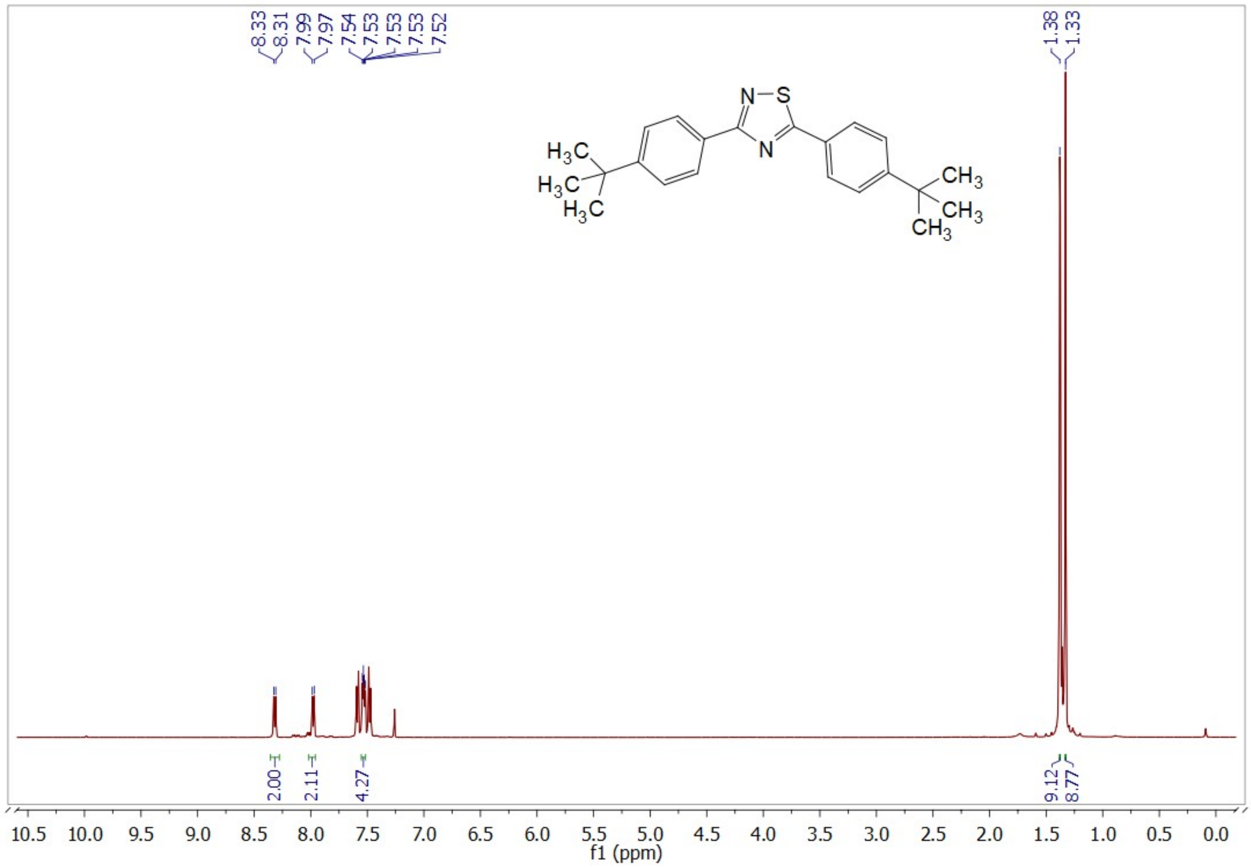

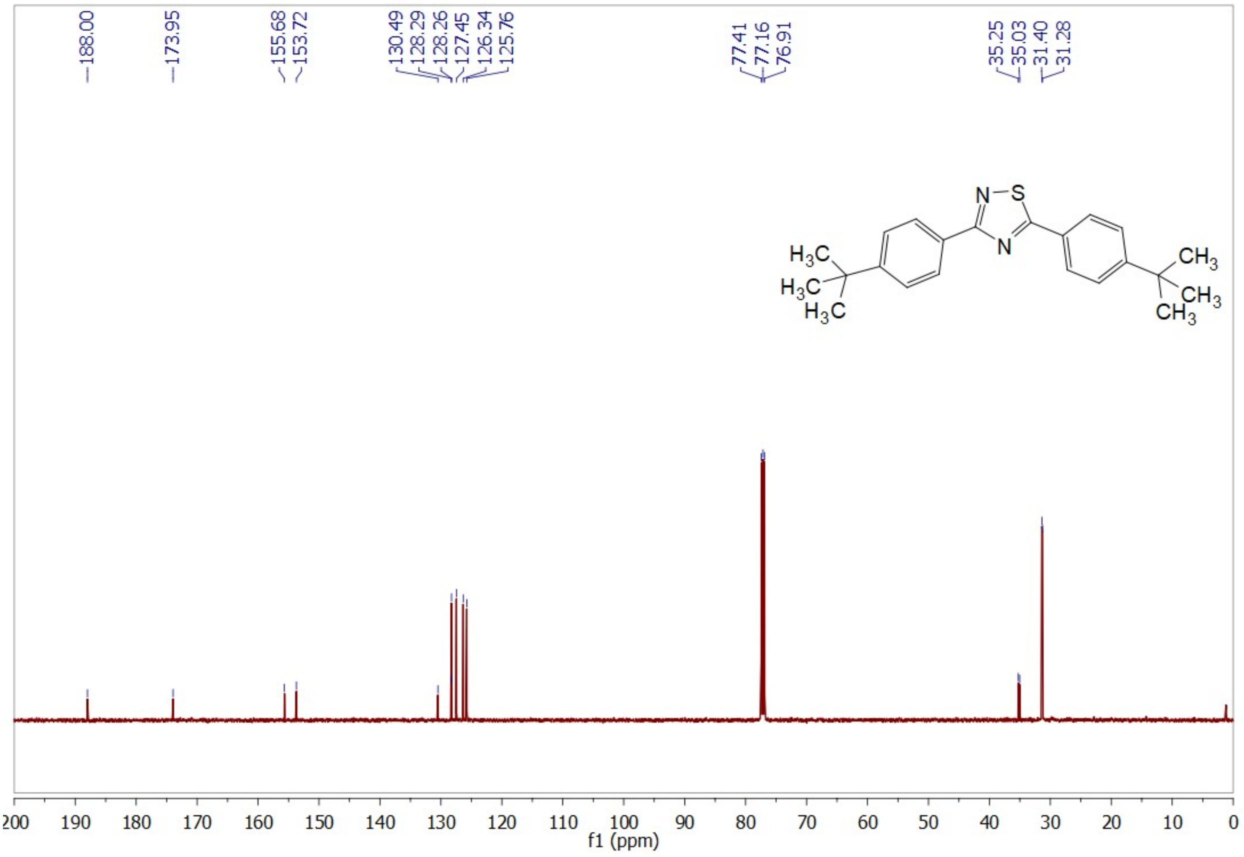

[4.2.4] 3,5-bis(4-aminophenyl)-1,2,4-thiadiazole in $CDCl_3$ (3d)

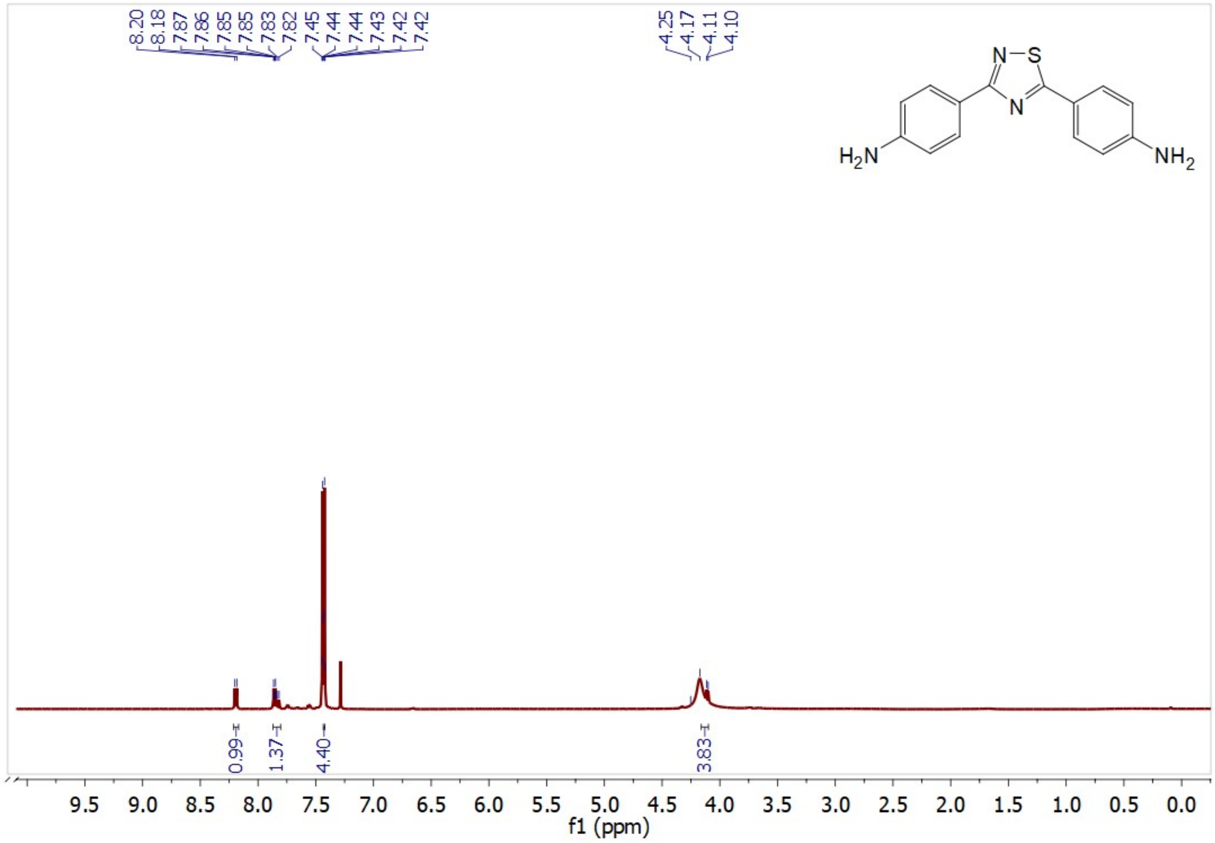

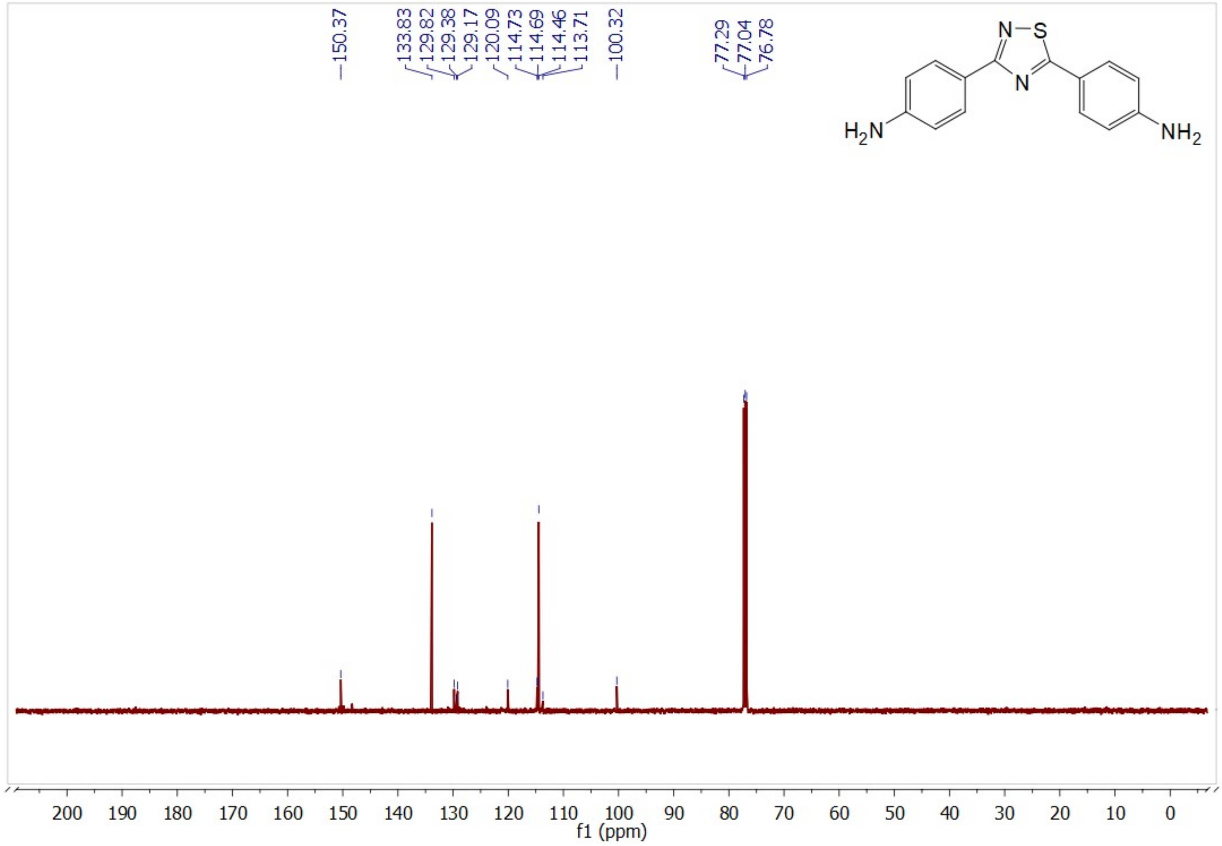

[4.2.2] 3,5 bis(4-methoxyphenyl)-1,2,4-thiadiazole in $CDCl_3$ (3e)

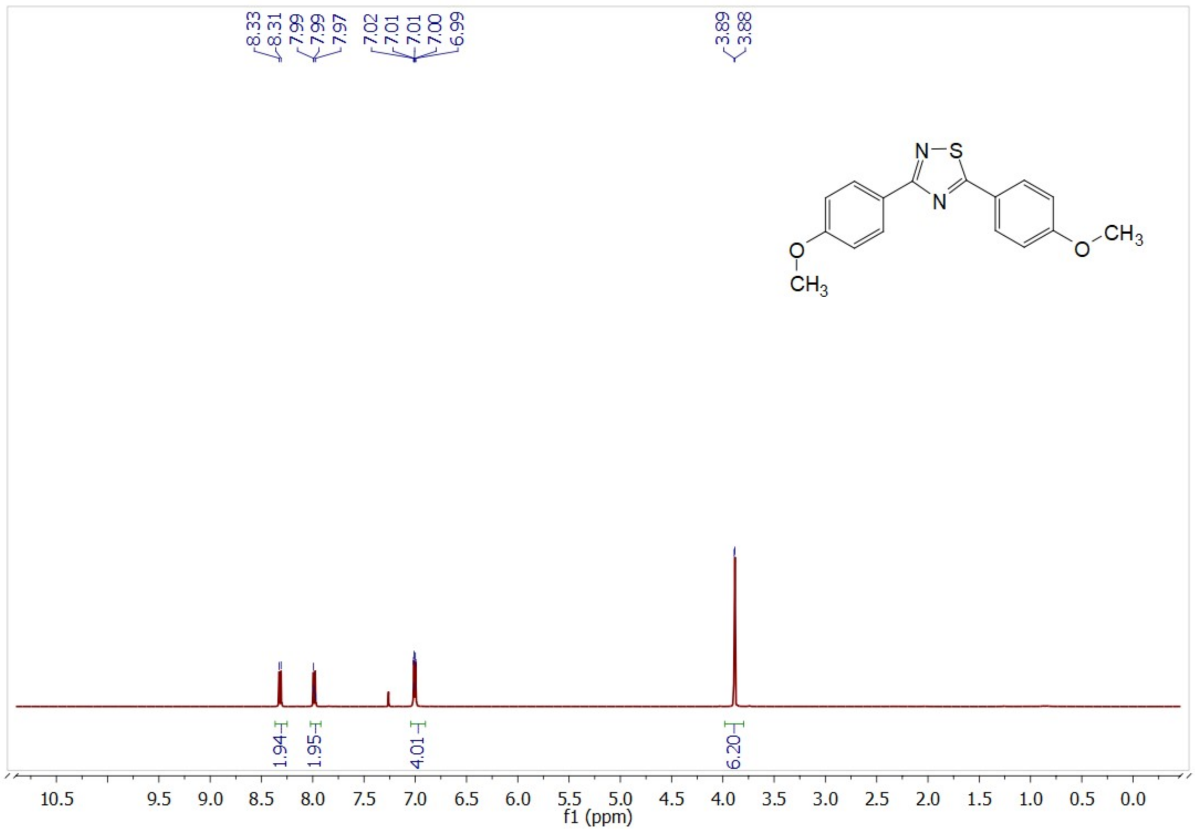

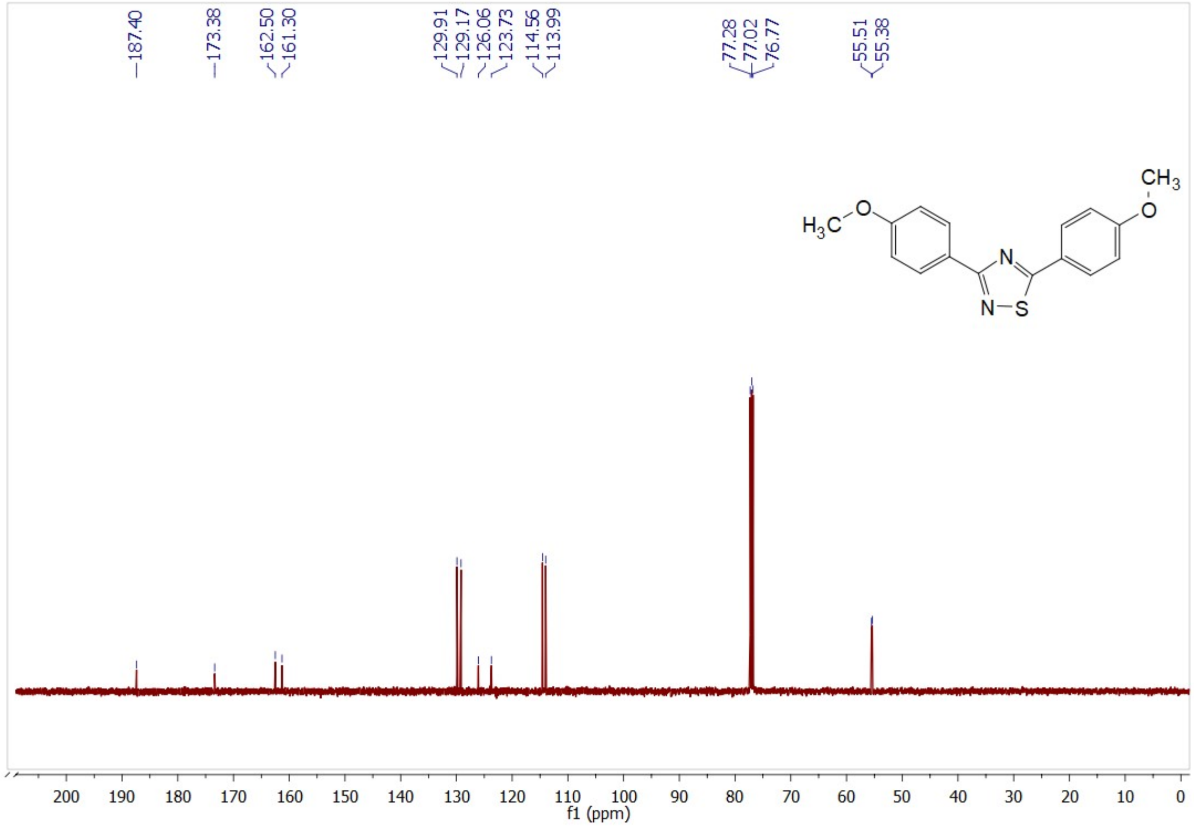

Mass spectrum of 3e

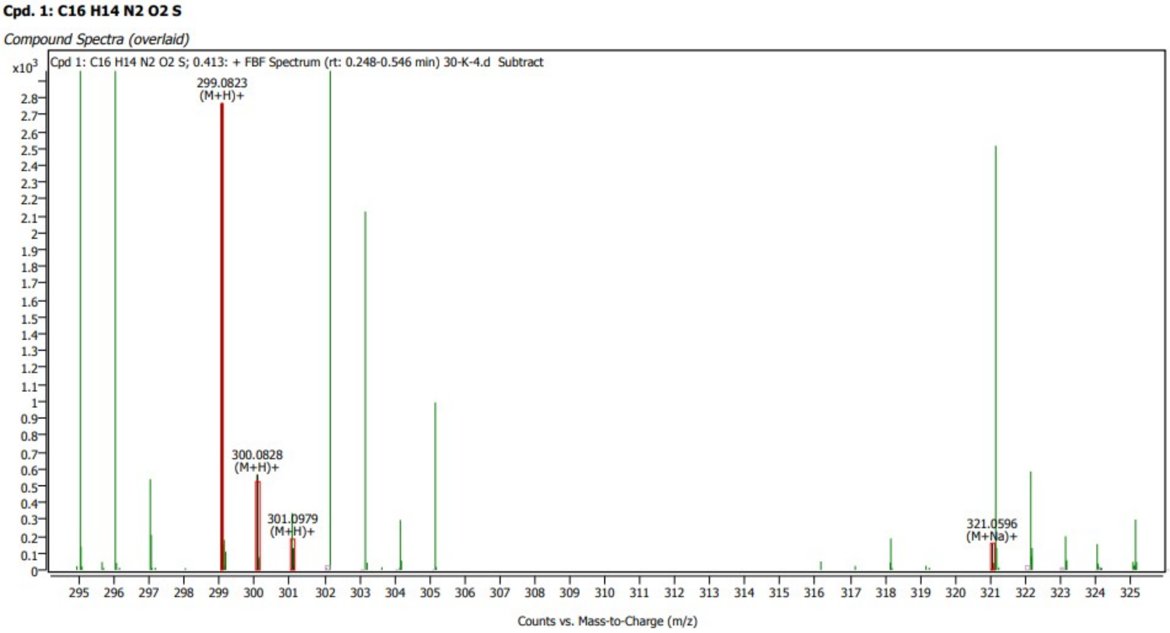

[4.2.6] 3,5-bis(4-fluorophenyl)-1,2,4-thiadiazole in $CDCl_3$ (3f)

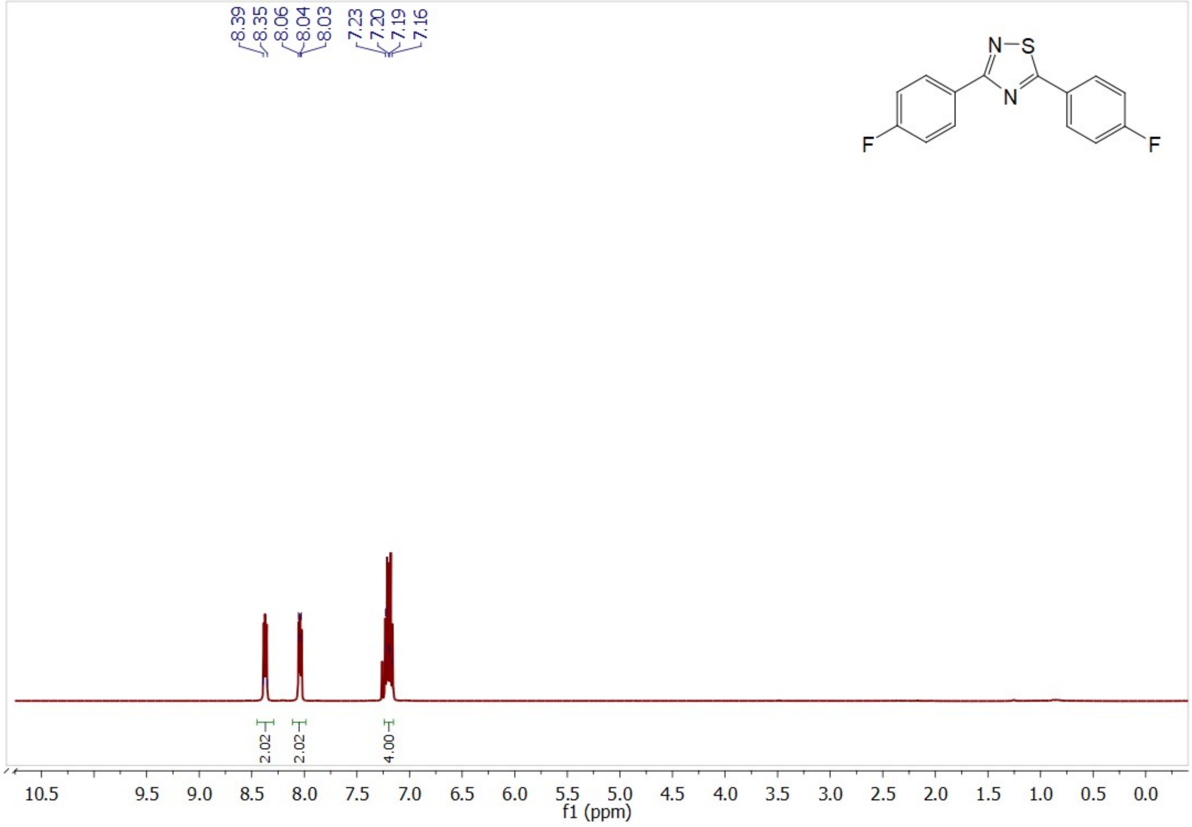

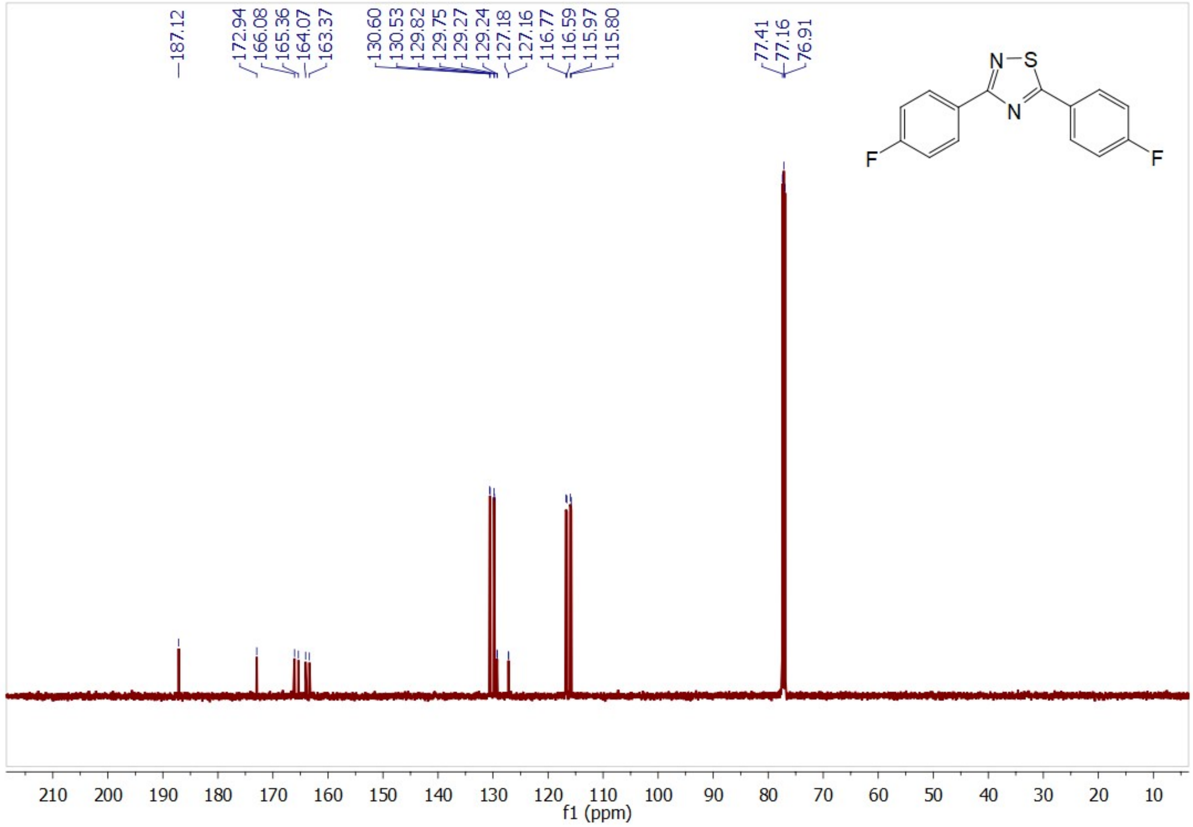

^{19}F NMR Spectra for 3,5-bis(4-fluorophenyl)-1,2,4-thiadiazole (3f)

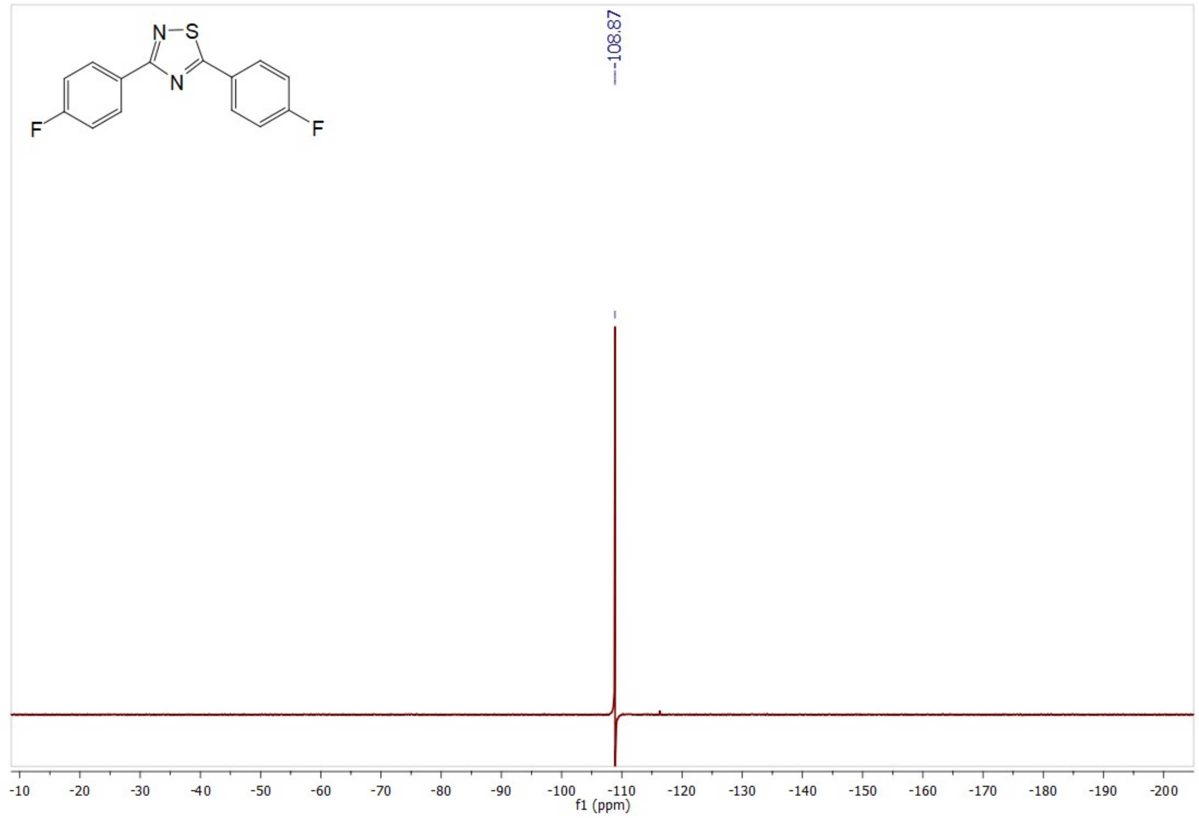

[4.2.7] 3,5-bis(4-chlorophenyl)-1,2,4-thiadiazole in $CDCl_3$ (3g)

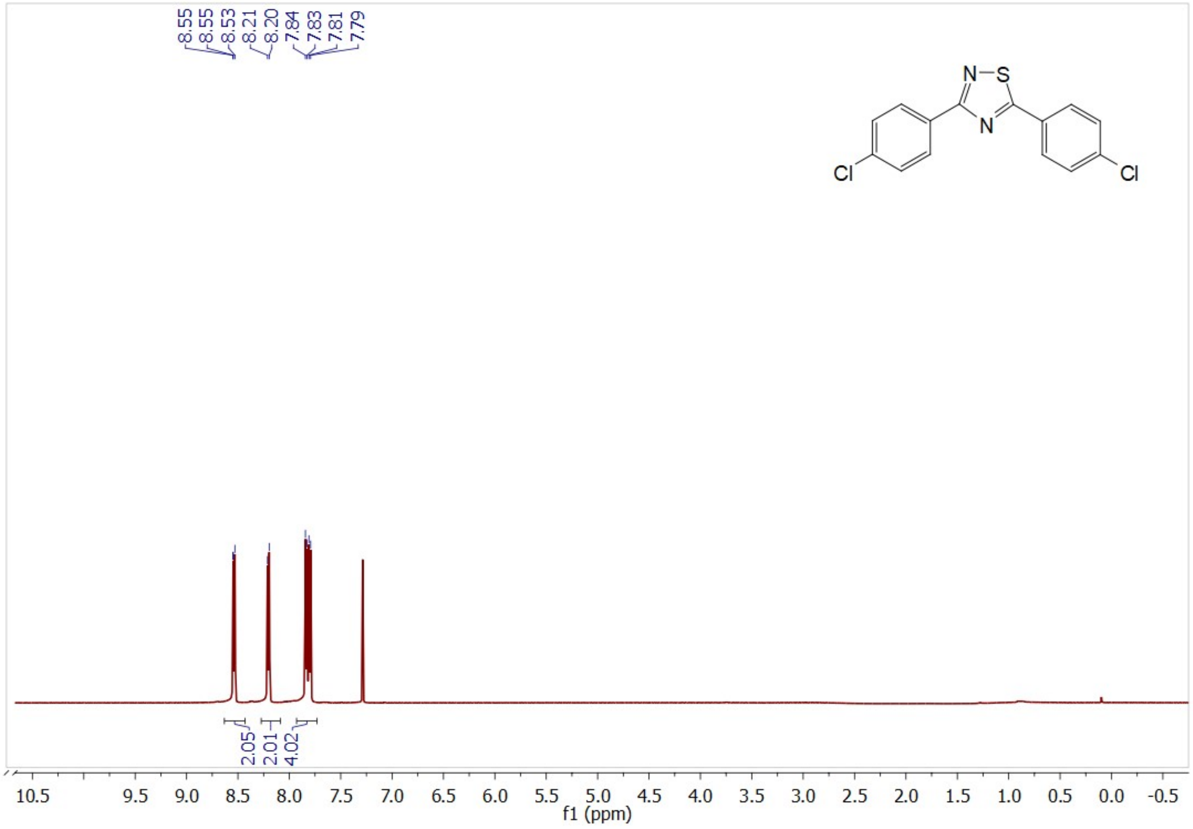

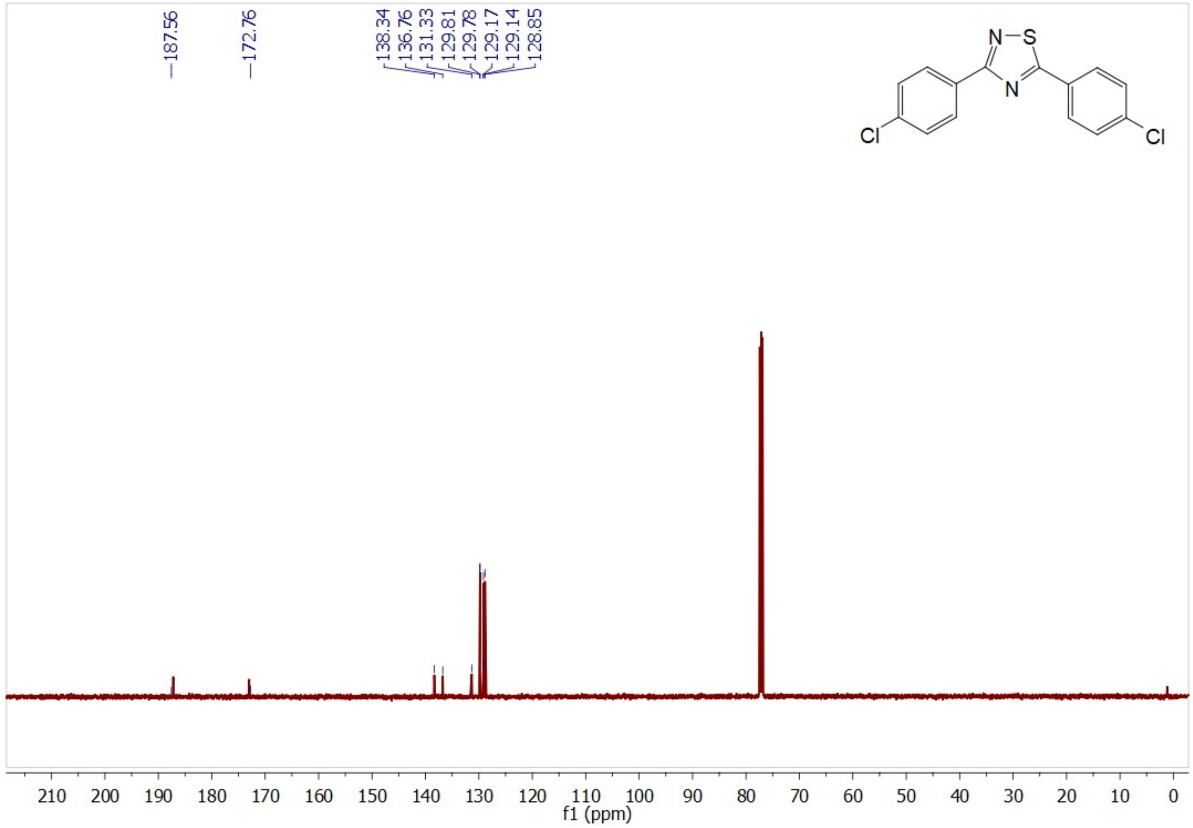

[4.2.8] 3,5-bis(3-chlorophenyl)-1,2,4-thiadiazole in $CDCl_3$ (3h)

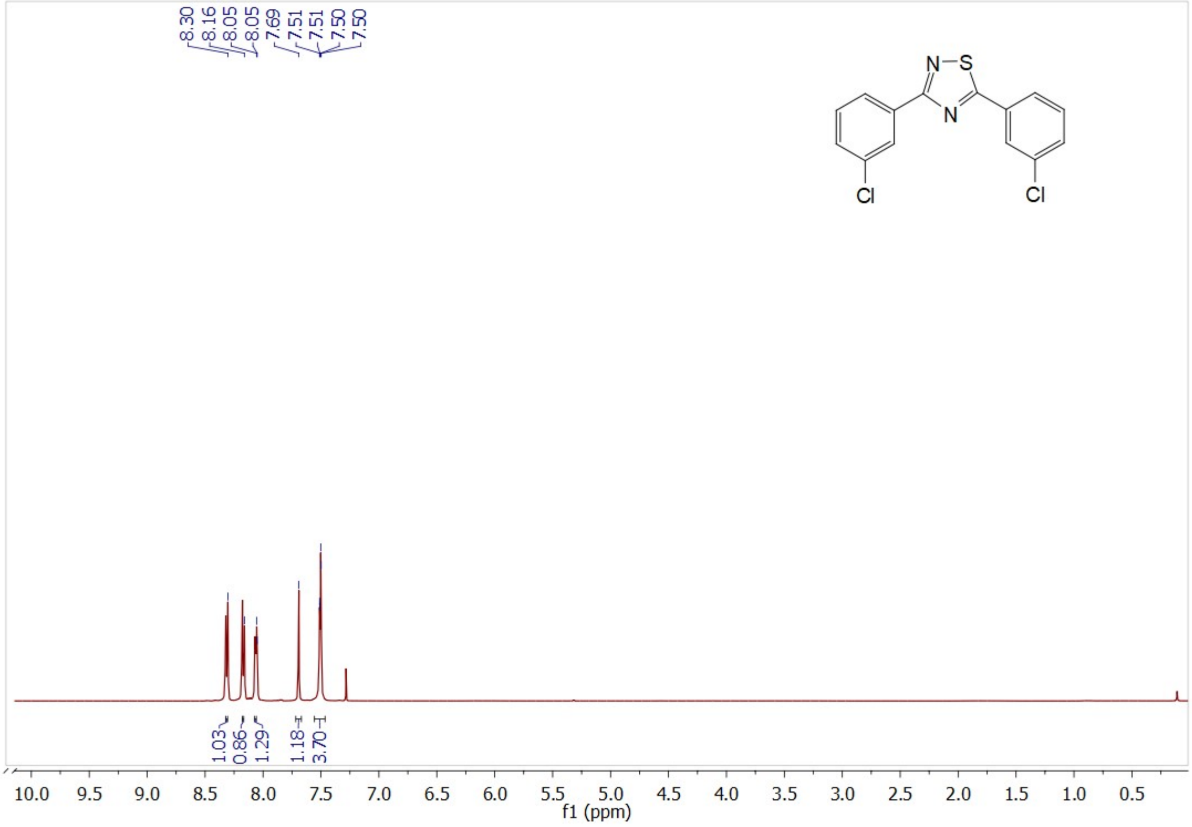

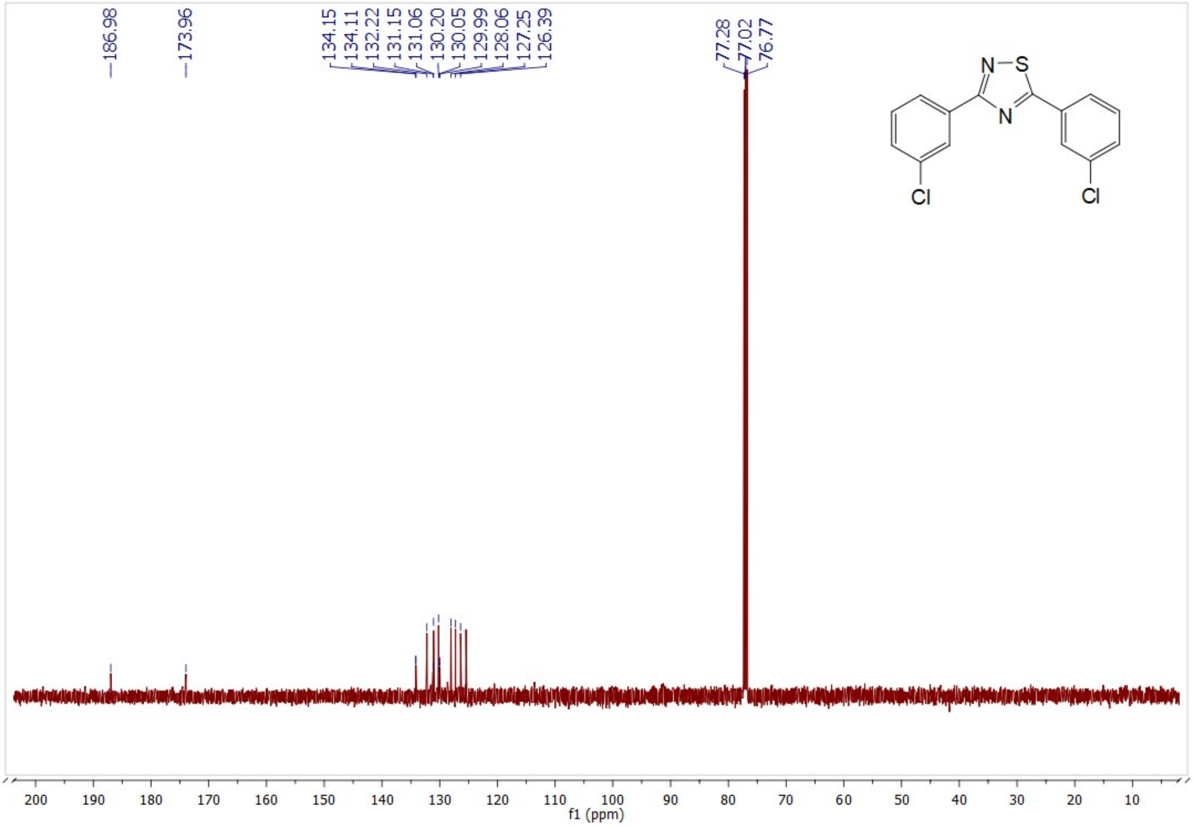

[4.2.9] 3,5-bis(2-chlorophenyl)-1,2,4-thiadiazole in $CDCl_3$ (3i)

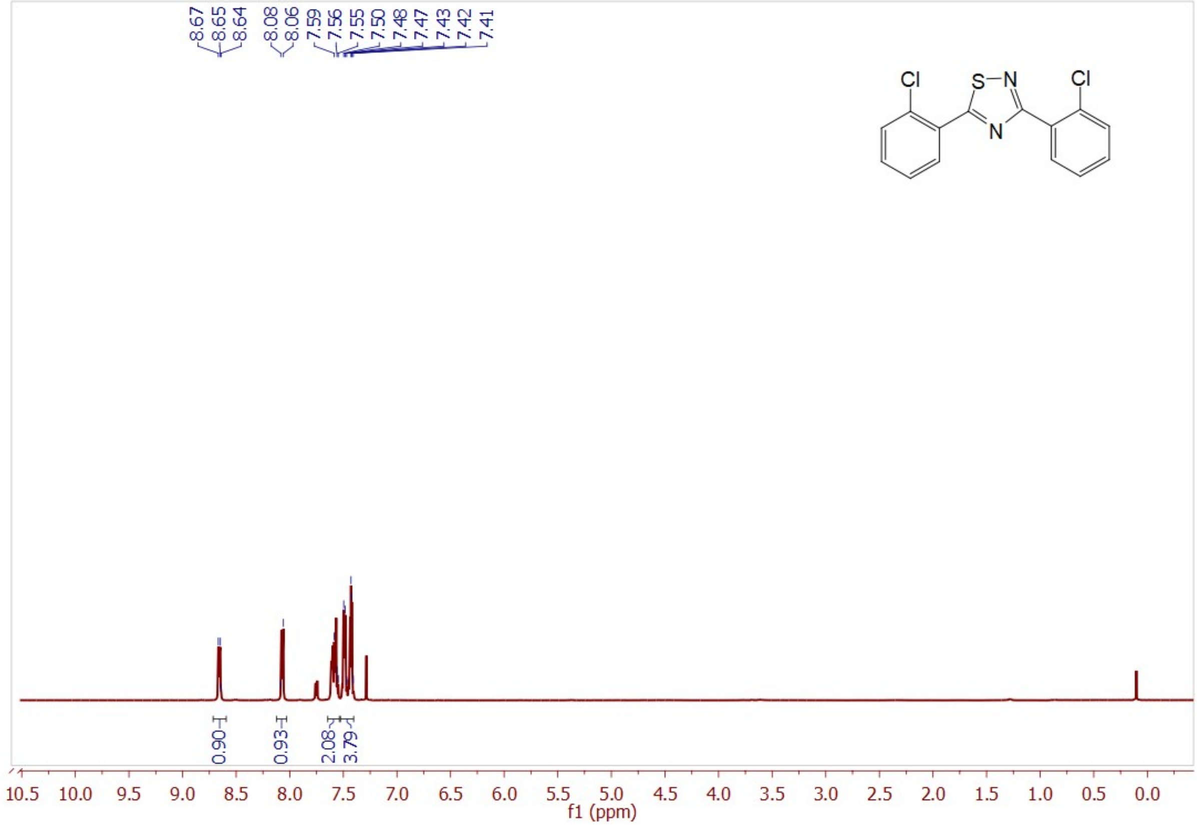

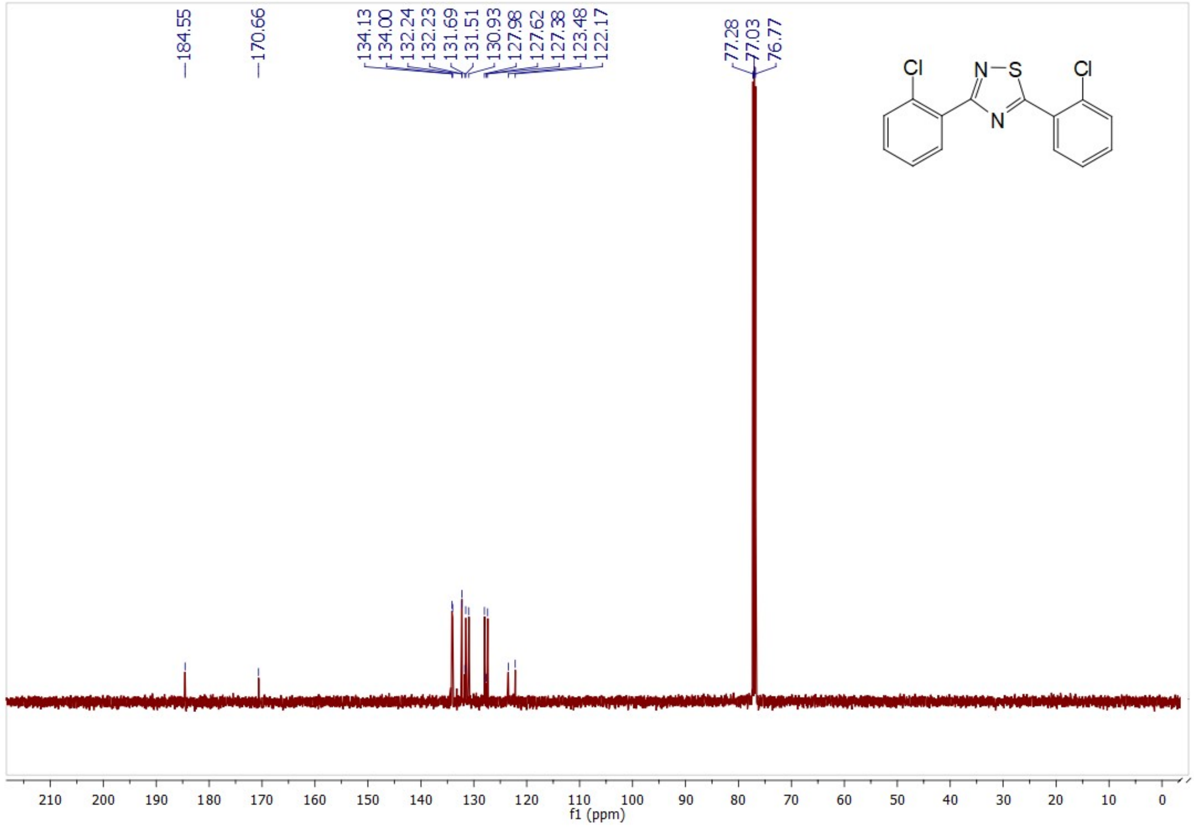

[4.2.10] 3,5-bis(4-bromophenyl)-1,2,4-thiadiazole in $CDCl_3$ (3j)

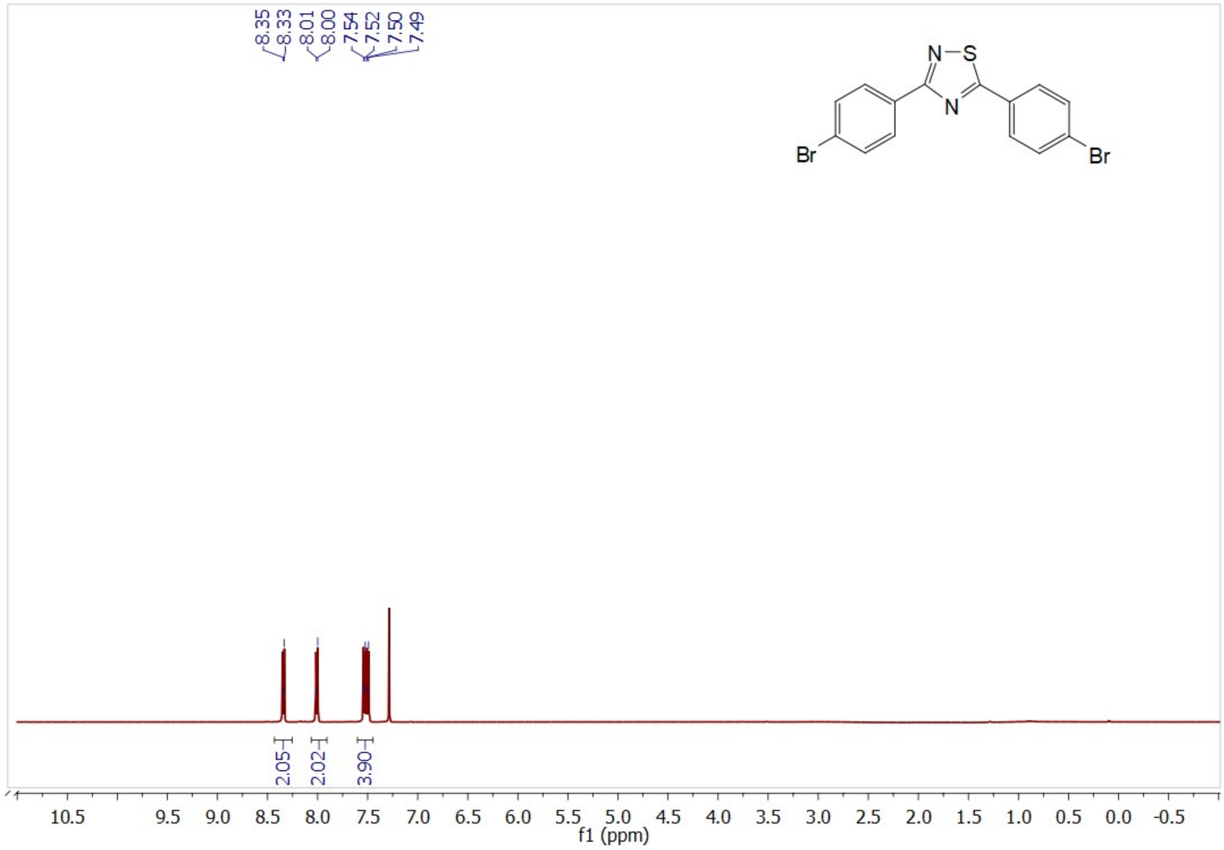

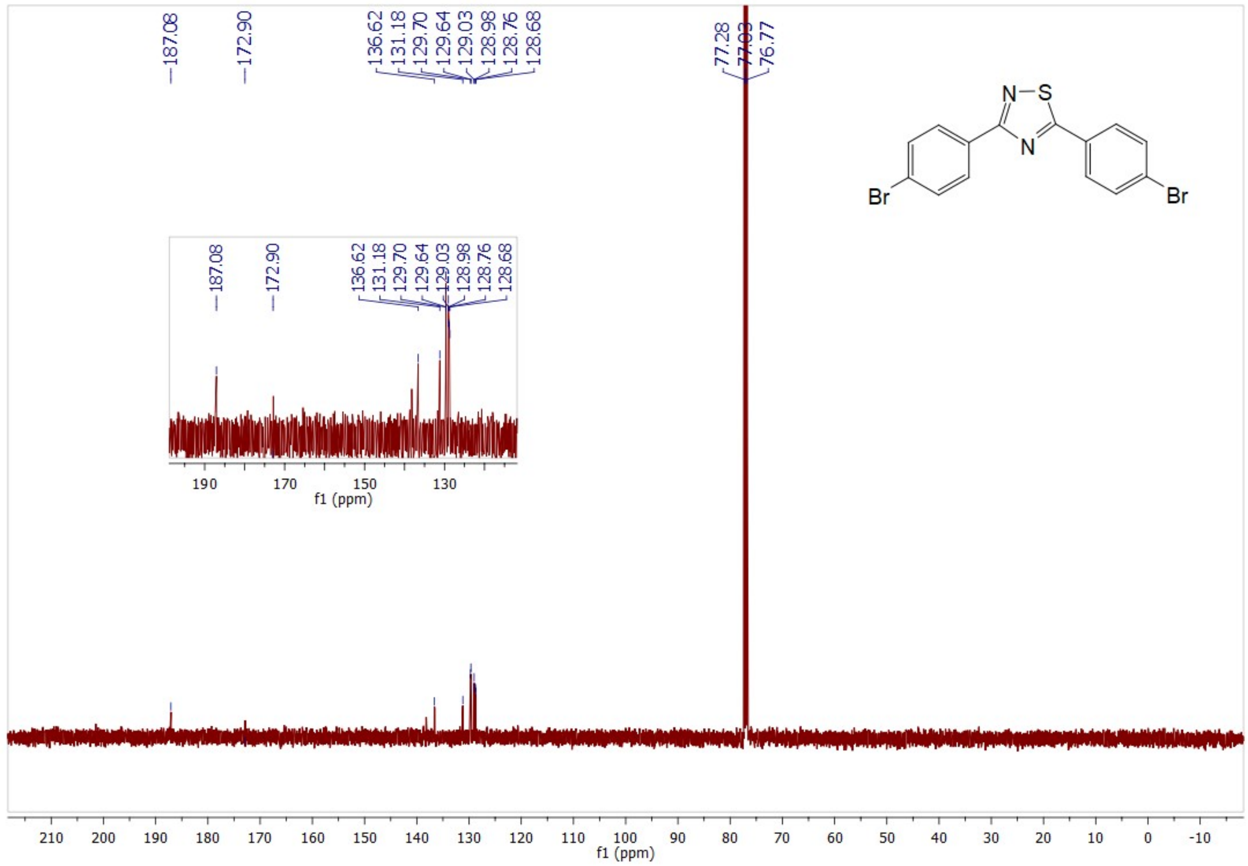

[4.2.11] 3,5-bis(3-bromophenyl)-1,2,4-thiadiazole in $CDCl_3$ (3k)

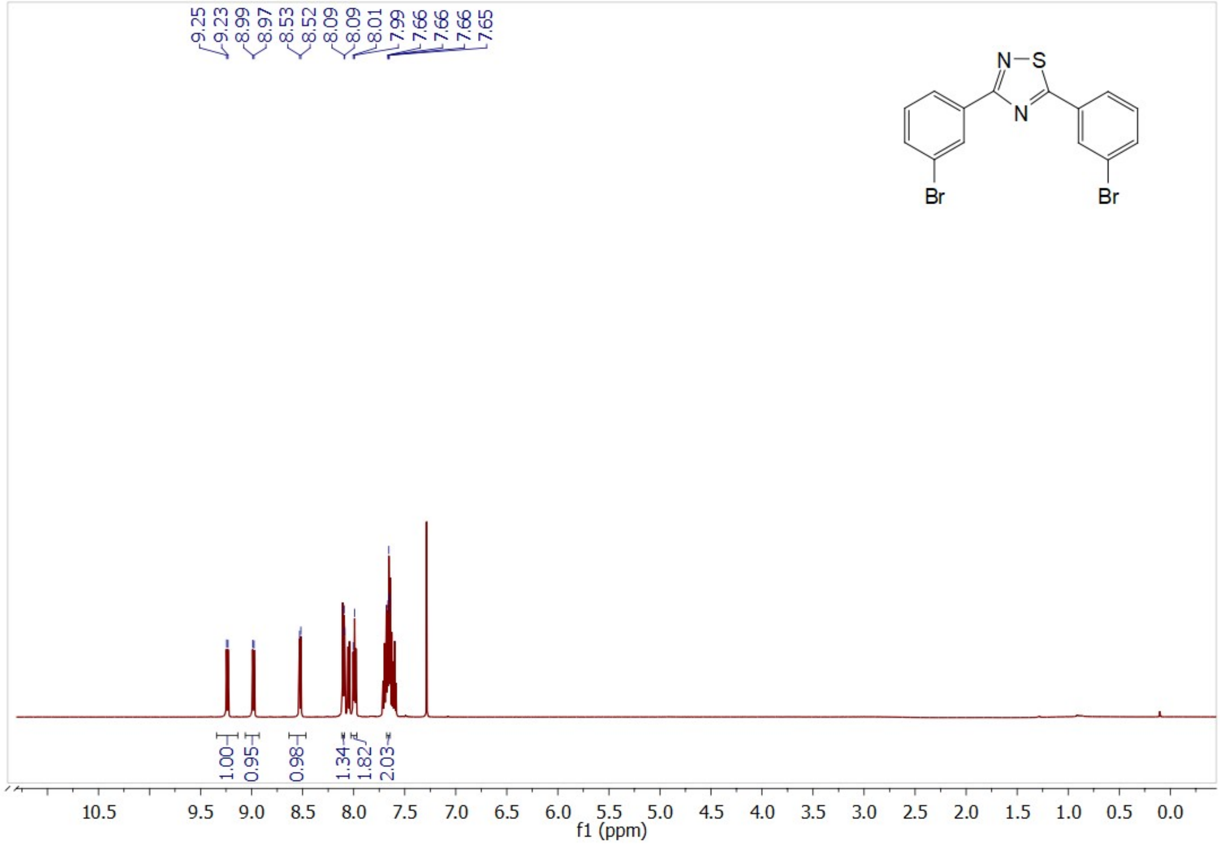

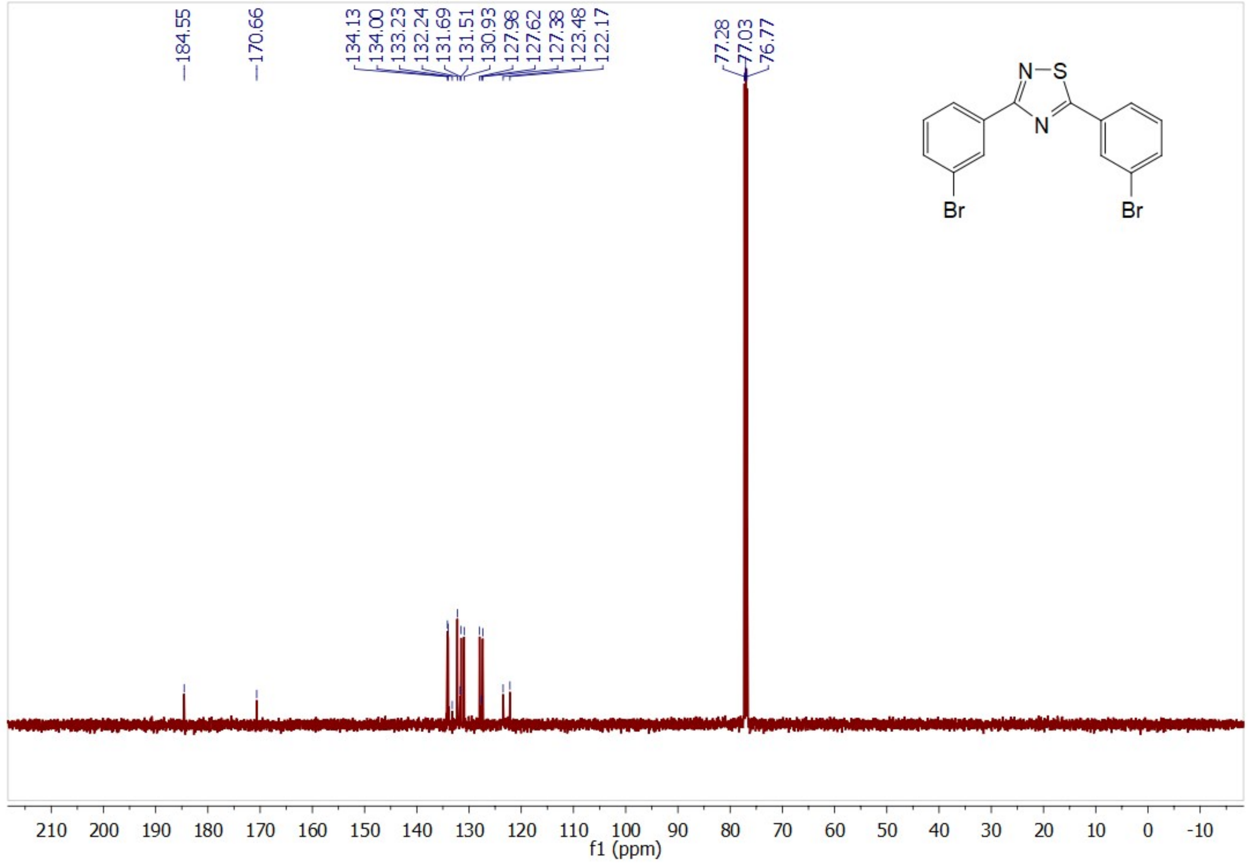

[4.2.12] 3,5-bis(2-bromophenyl)-1,2,4-thiadiazole in $CDCl_3$ (3l)

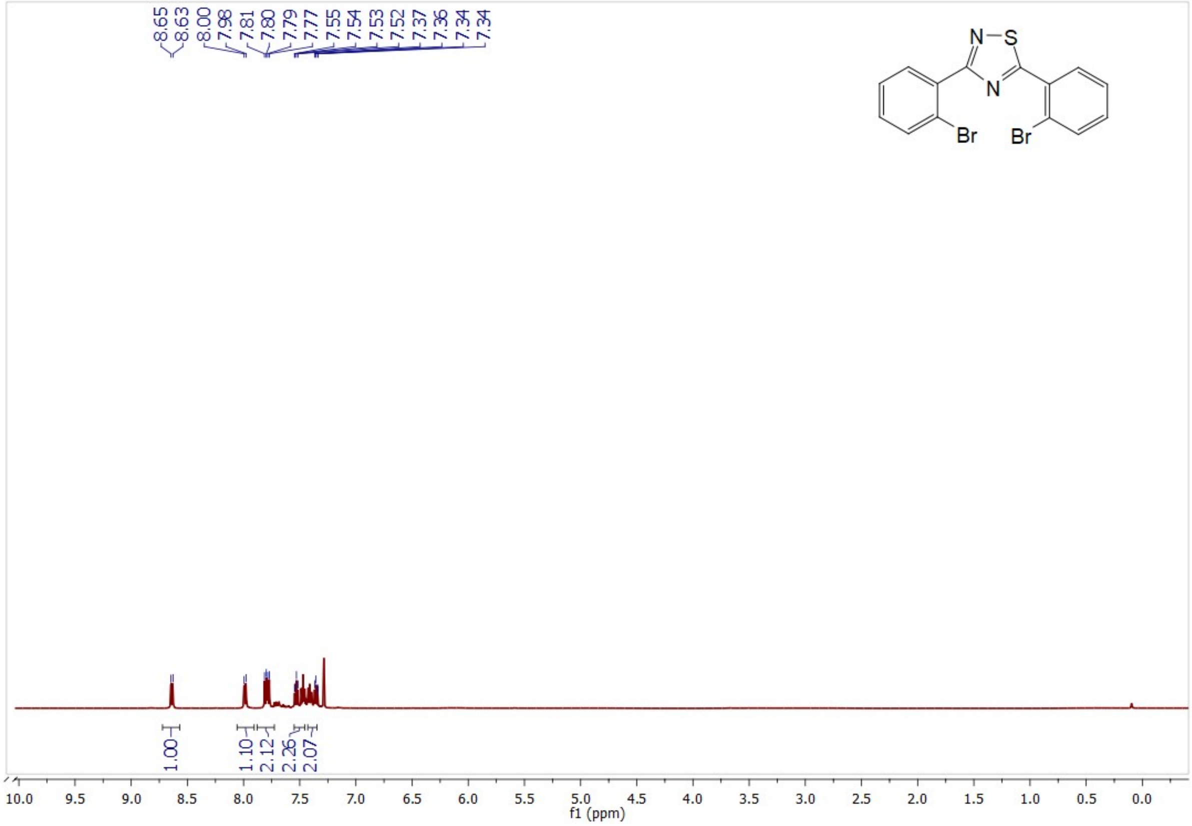

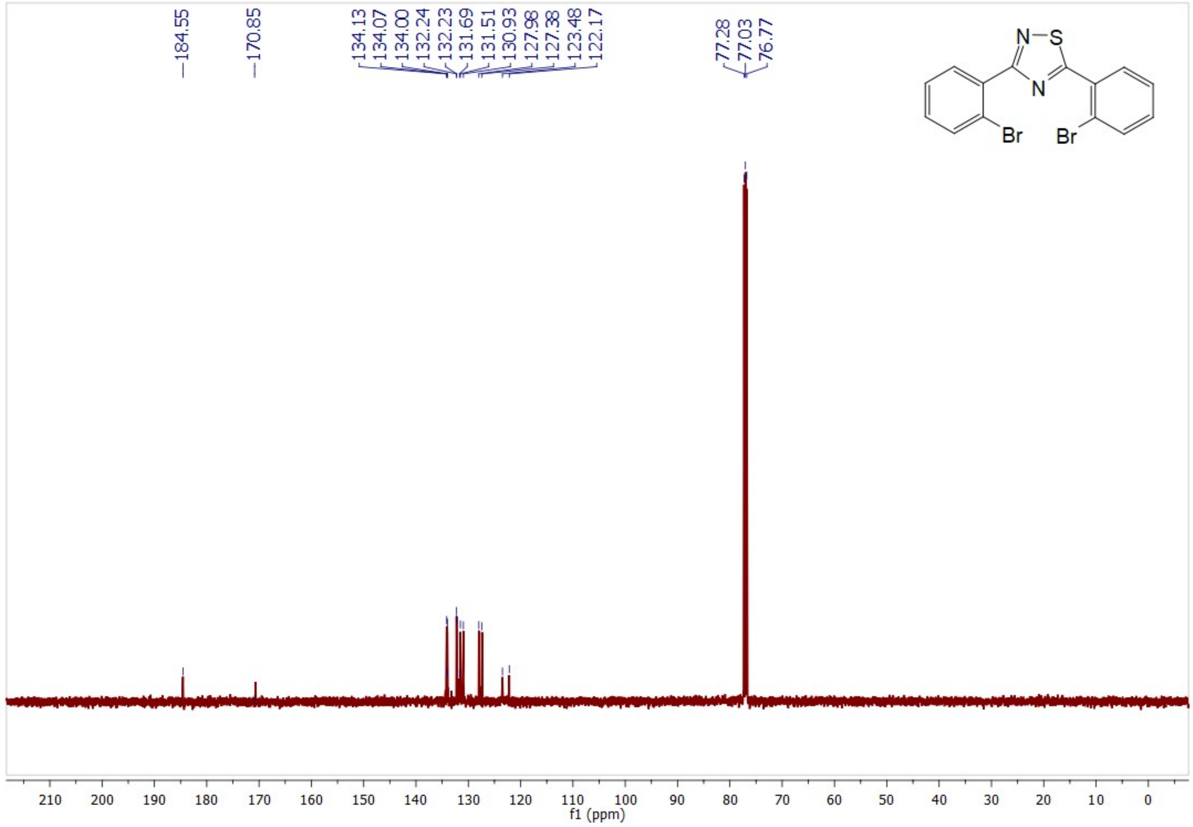

[4.2.13] 3,5-bis(trifluoromethyl)phenyl-1,2,4-thiadiazole in $CDCl_3$ (3m)

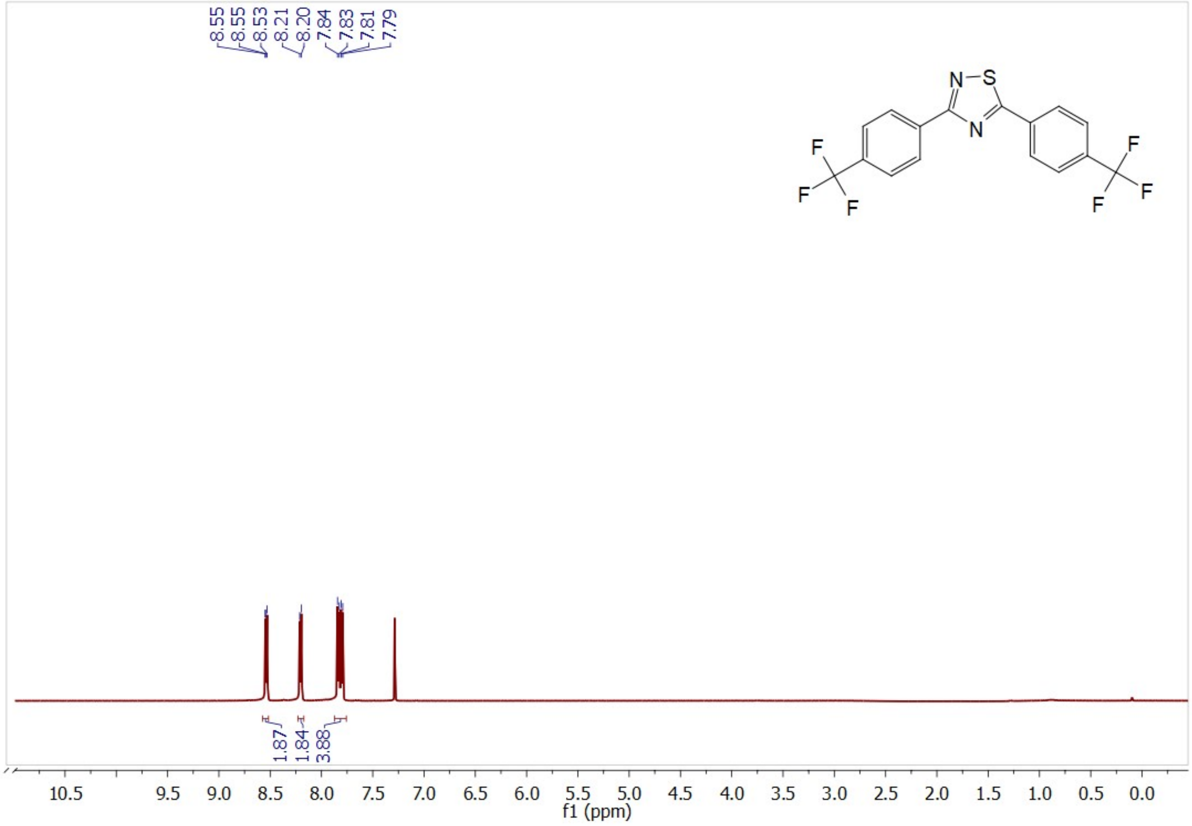

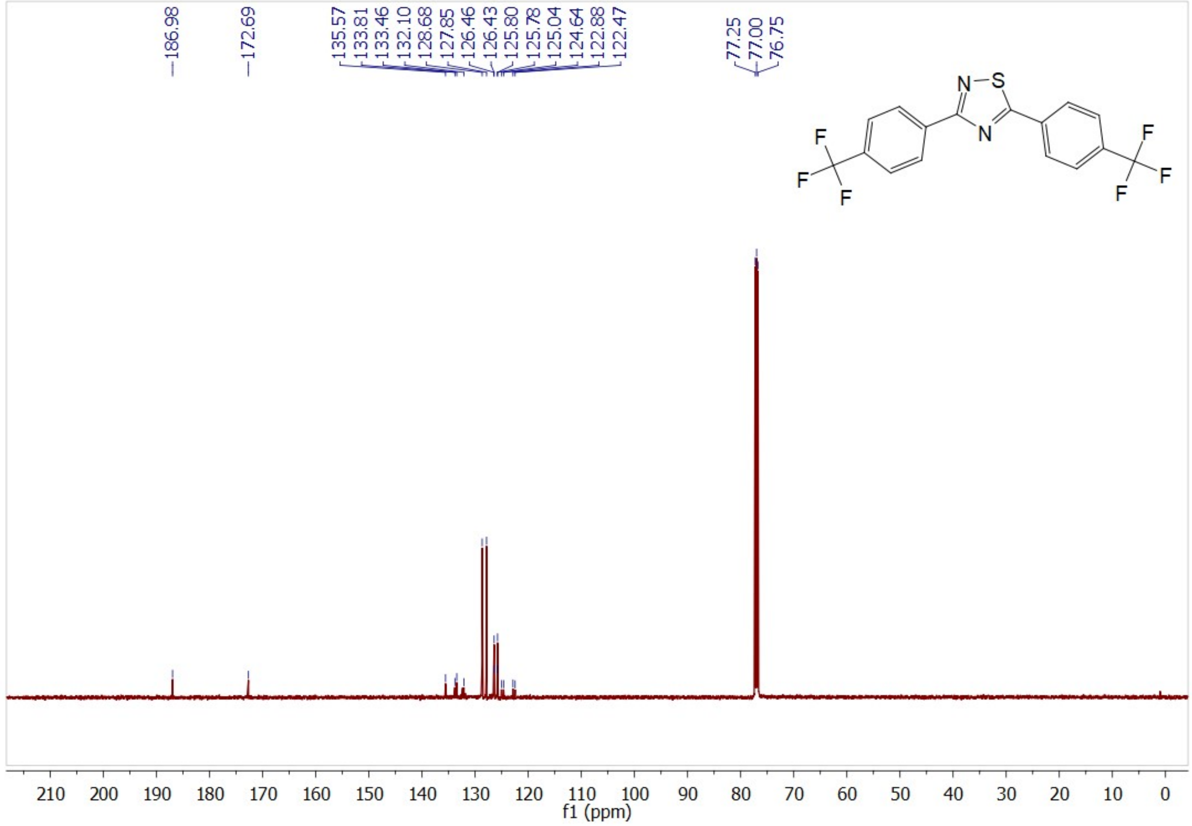

^{19}F NMR Spectra for3,5-bis(trifluoromethyl)phenyl-1,2,4-thiadiazole in $CDCl_3$ (3m)

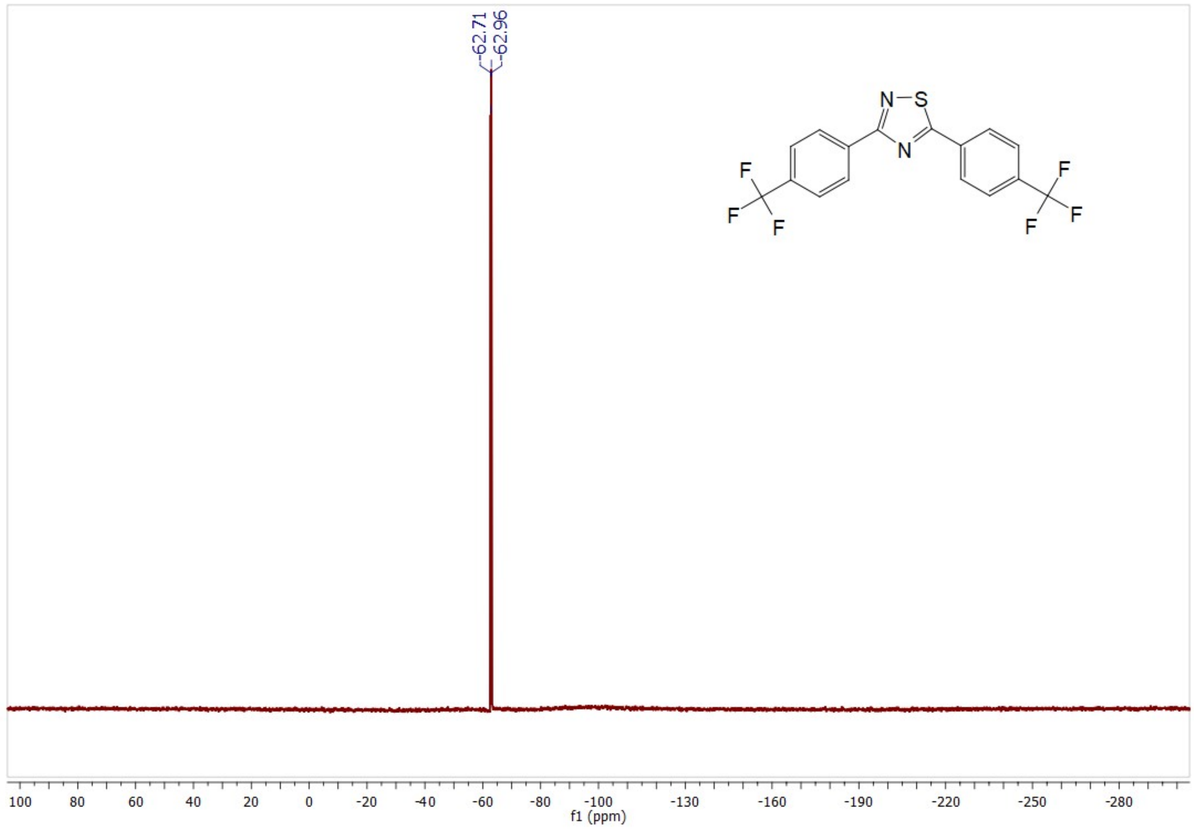

[4.2.14] 3,5-bis(4-nitrophenyl)-1,2,4-thiadiazole in $CDCl_3$ (3n)

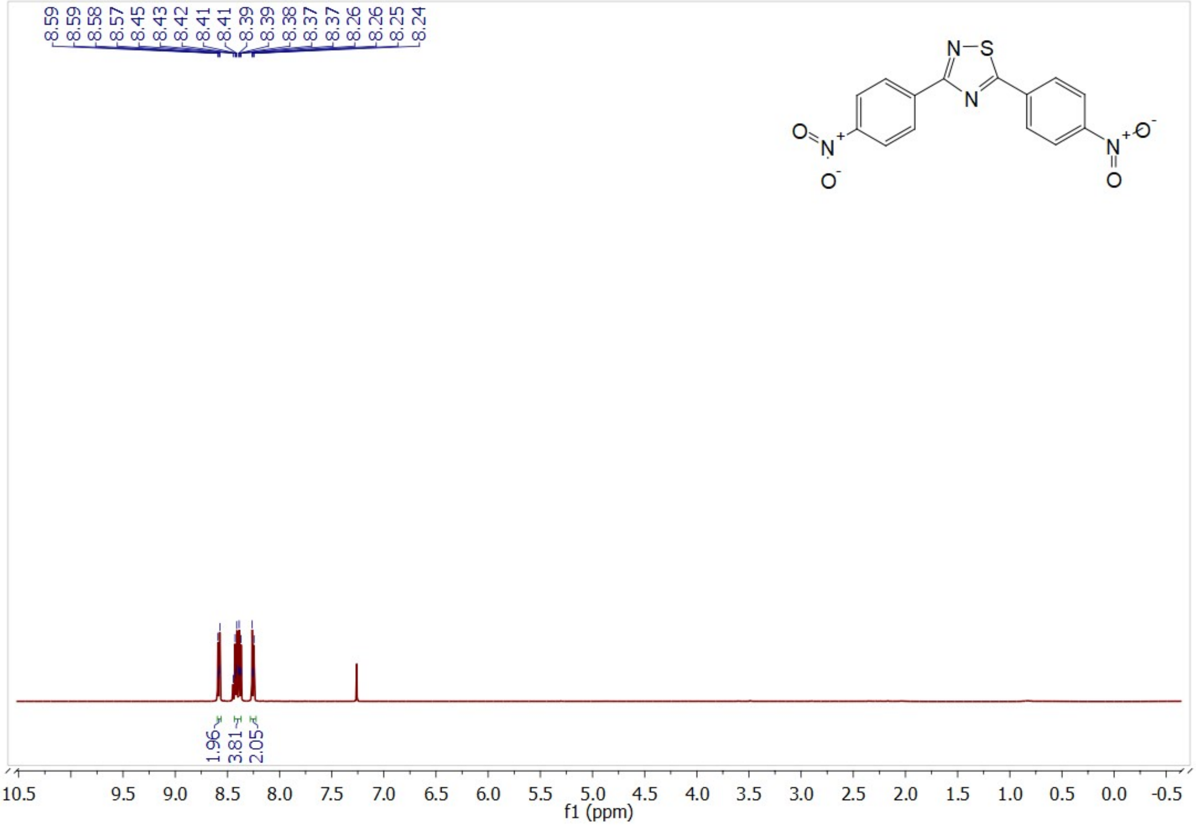

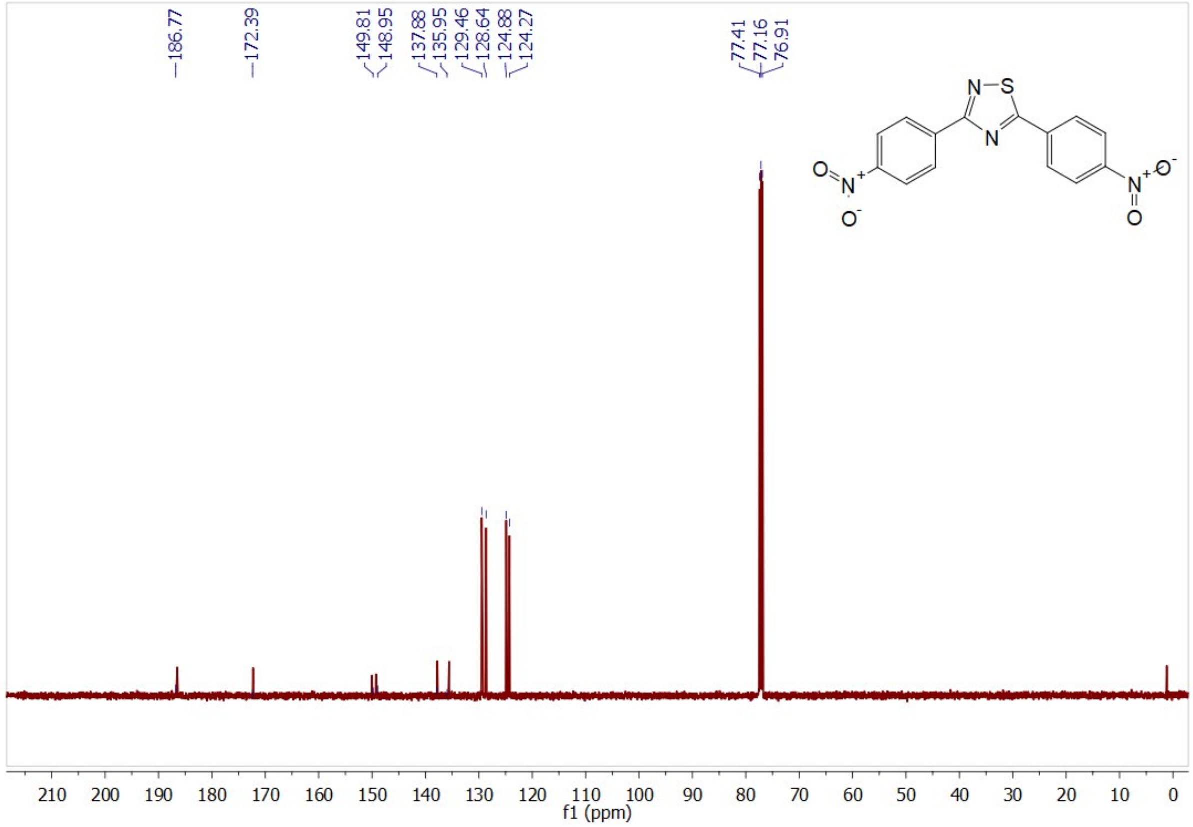

[4.2.15] 3,5-di(pyridine-2-yl)-1,2,4-thiadiazole in $CDCl_3$ (3o)

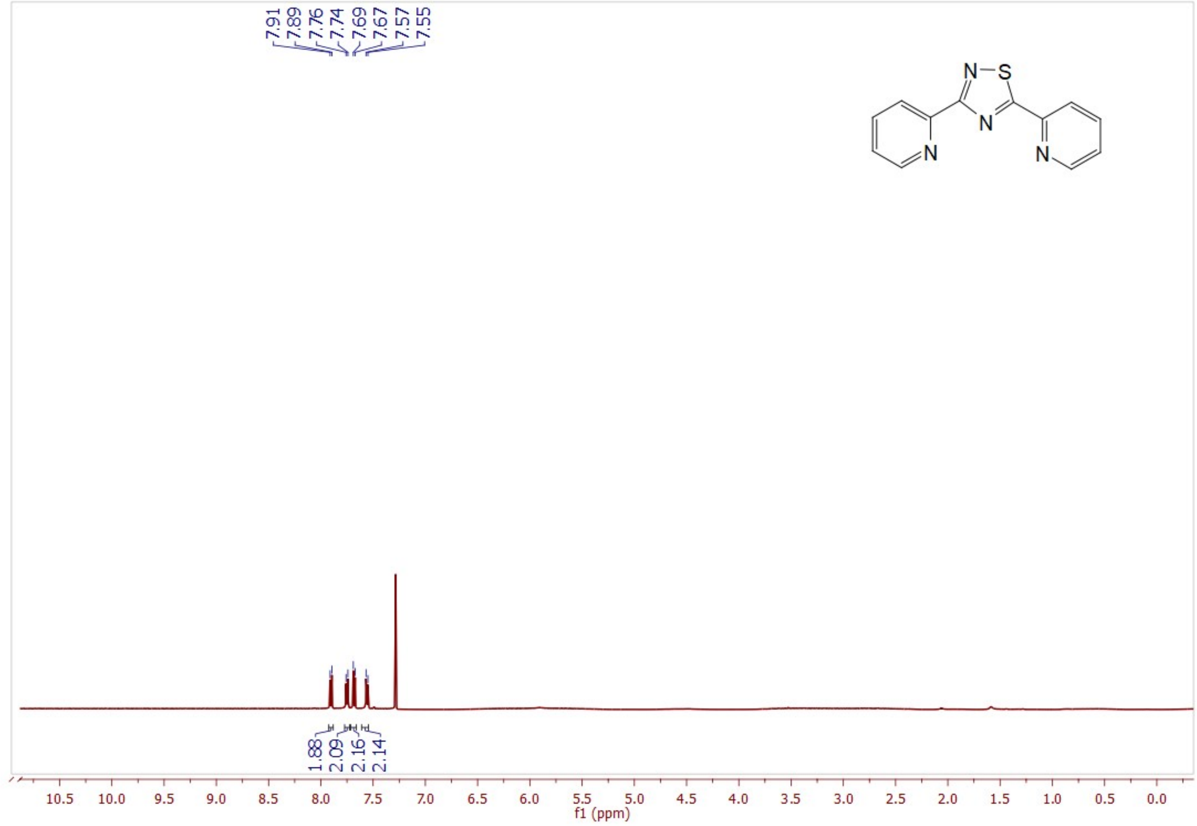

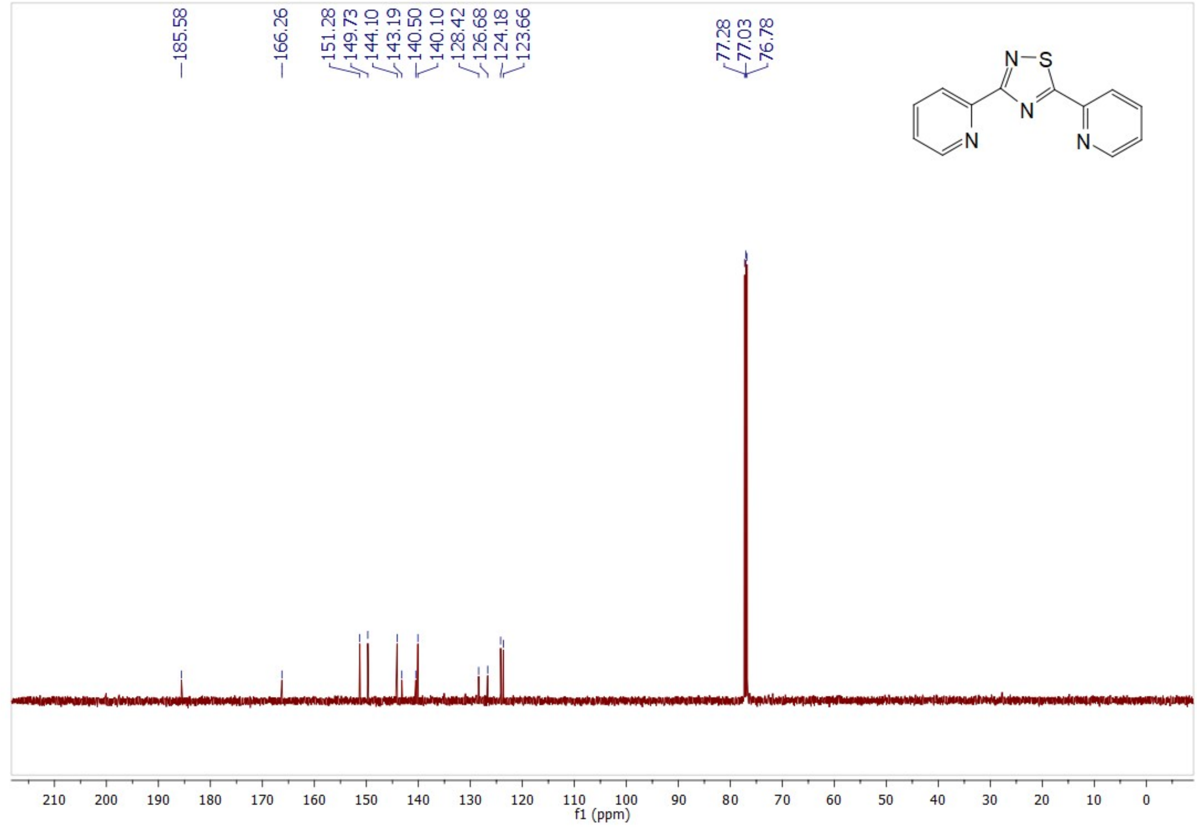

[4.2.16] 3,5-di(pyridine-4-yl)-1,2,4-thiadiazole in $CDCl_3$ (3p)

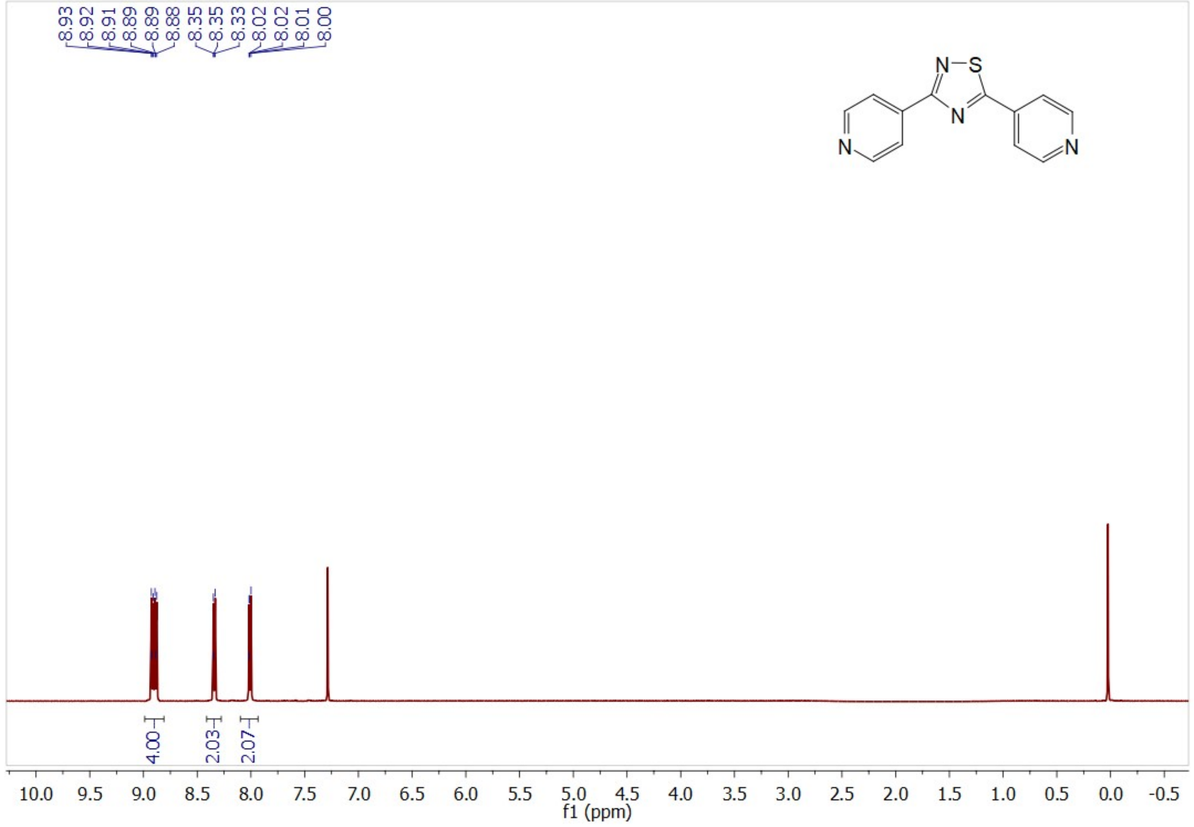

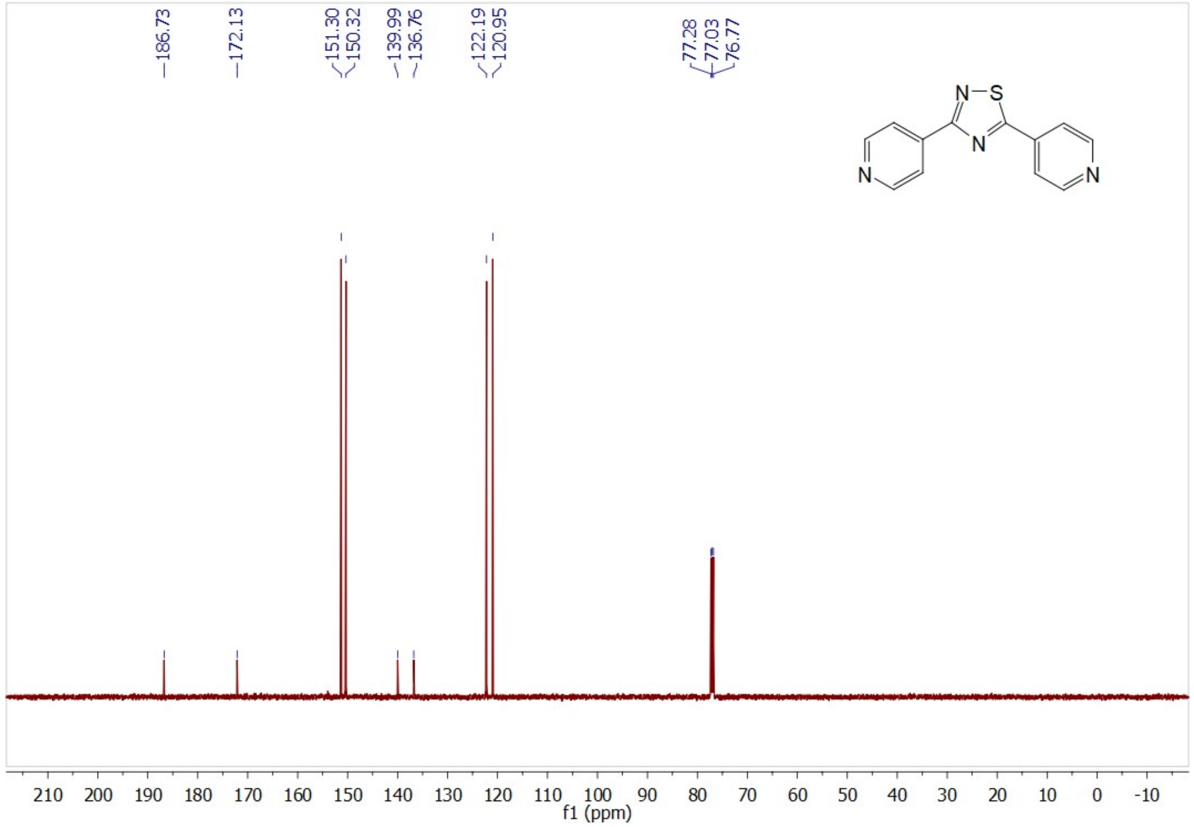

[4.2.17] 3,5-di(thiophen-2-yl)-1,2,4-thiadiazolein $CDCl_3$ (3q)

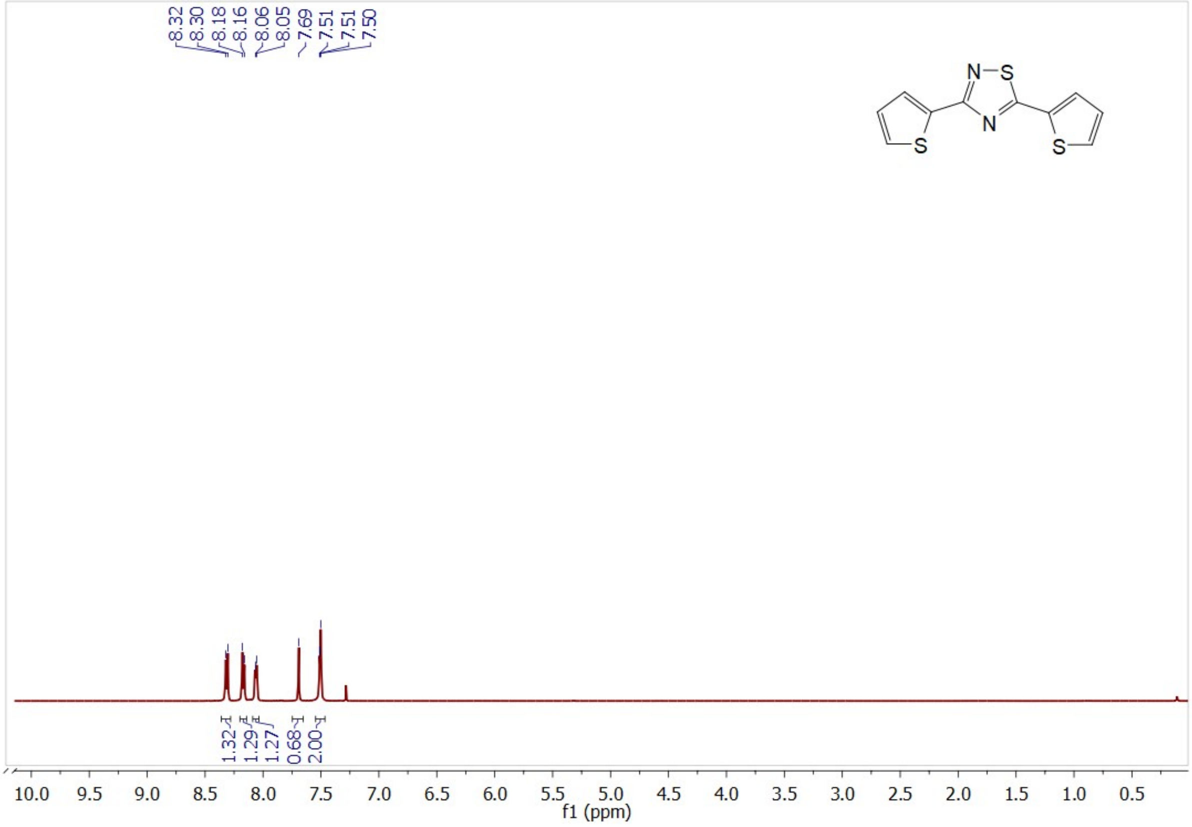

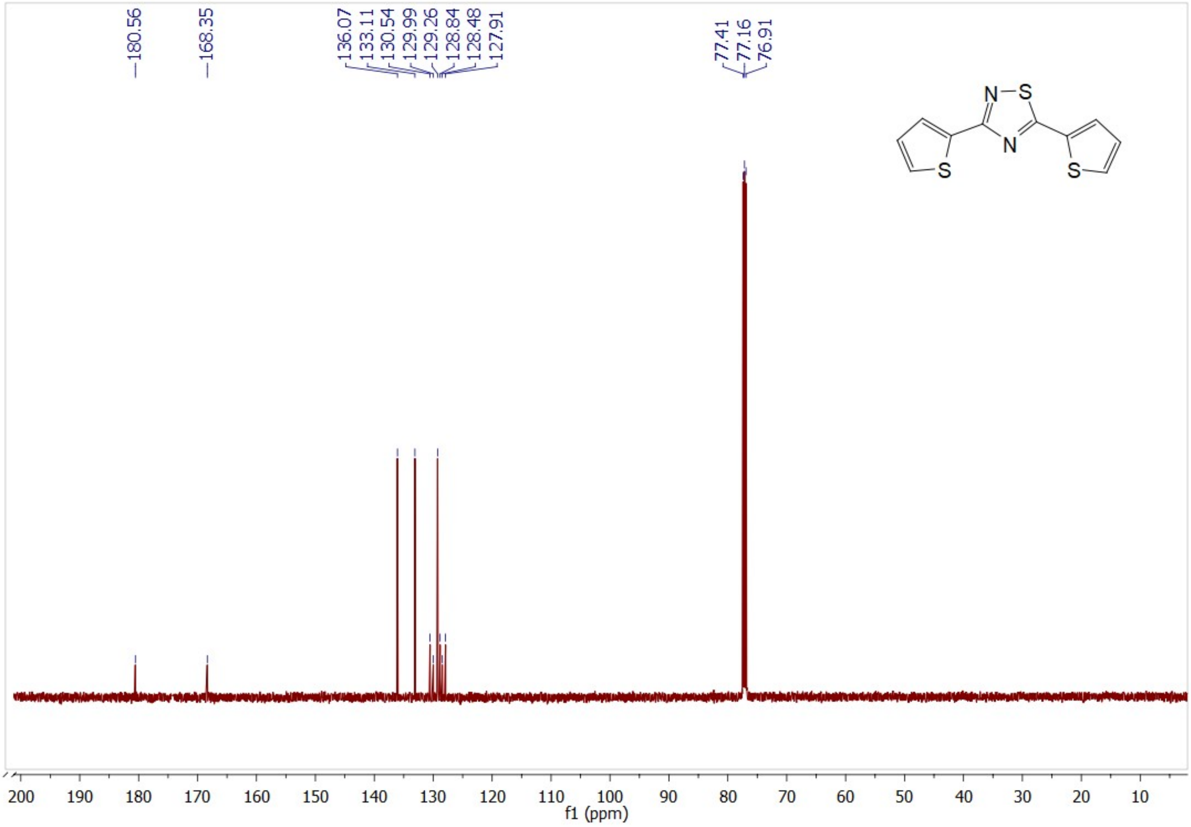

[4.1.18] 3,5-di-furan-2-yl-[1,2,4]thiadiazole (3r)

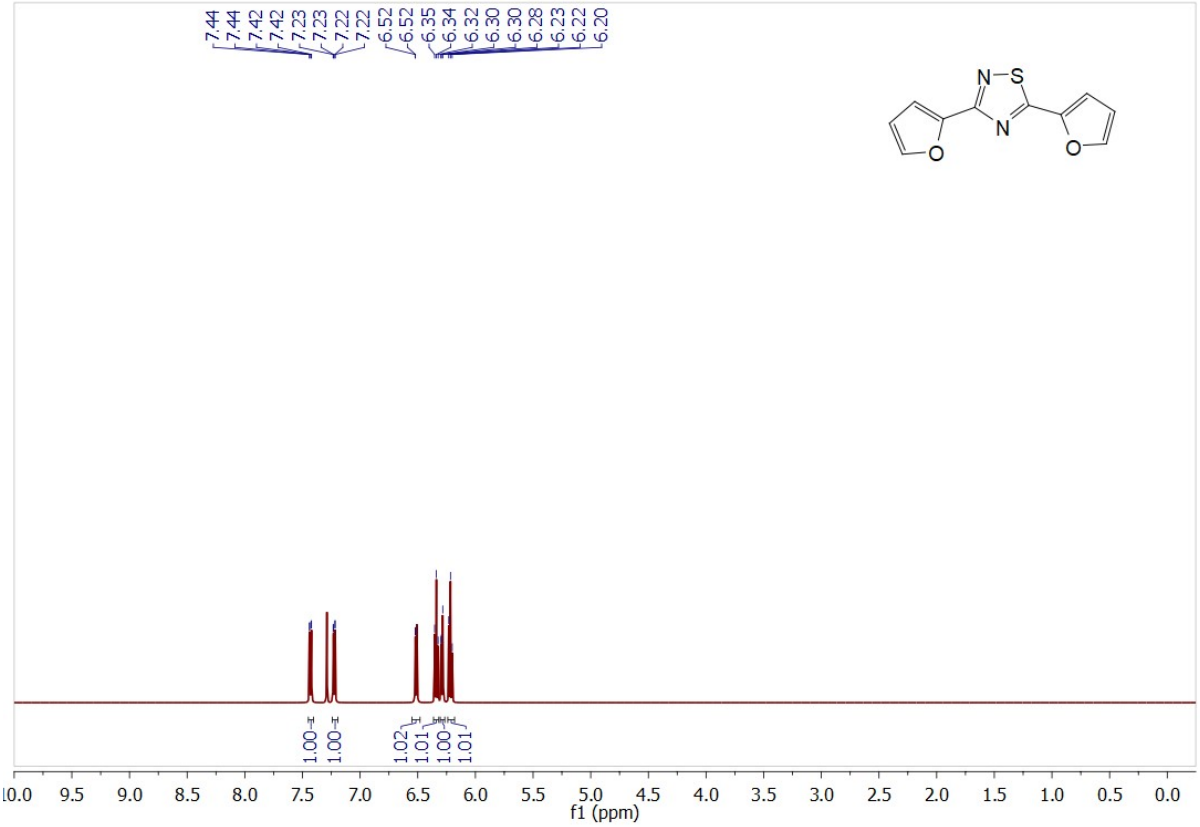

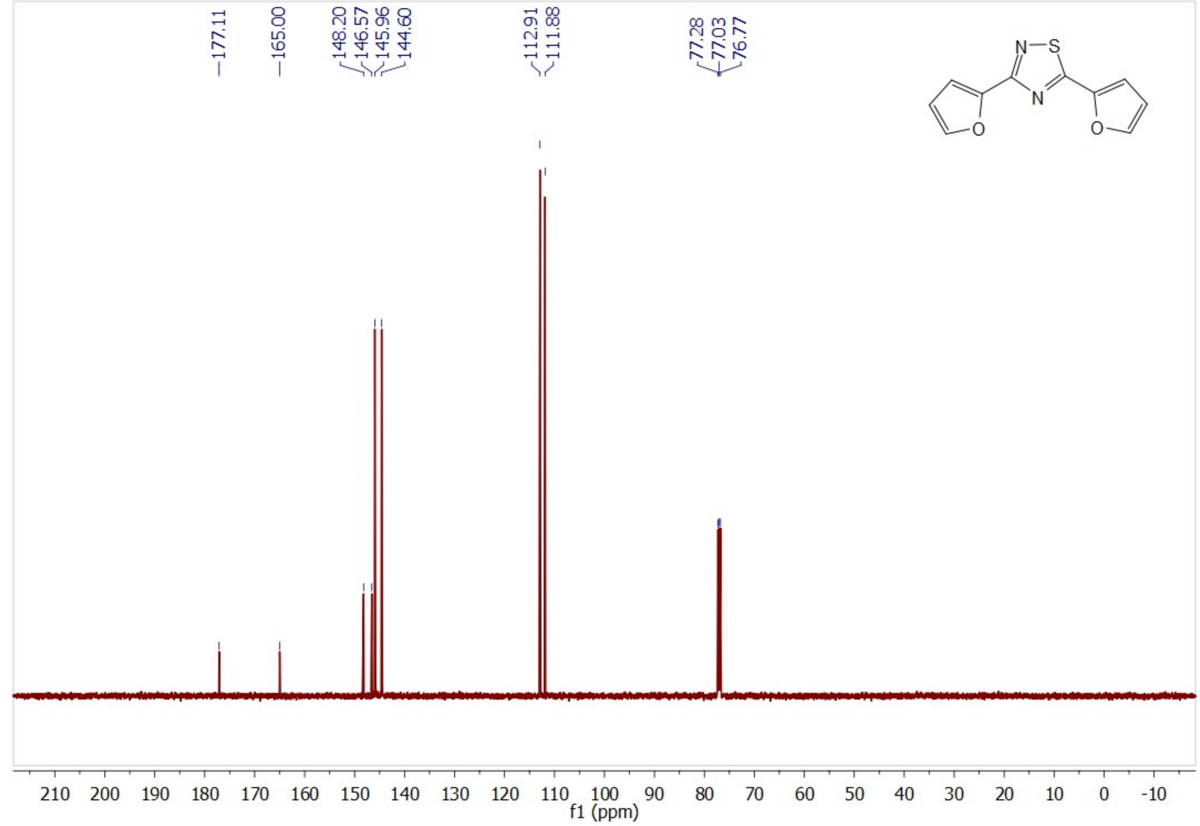

[4.2.19] 3,5 di(naphthalalen-1-yl)-1,2,4-thiadiazole in $CDCl_3$ (3s)

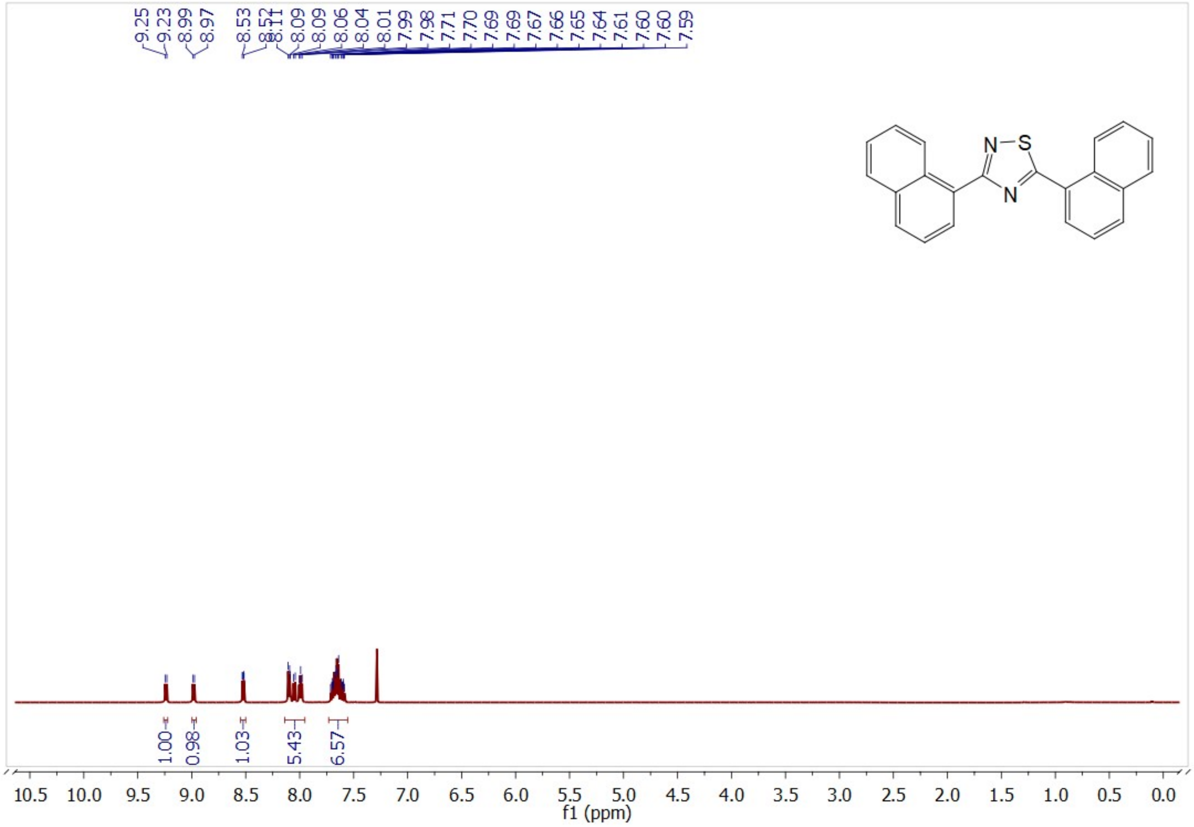

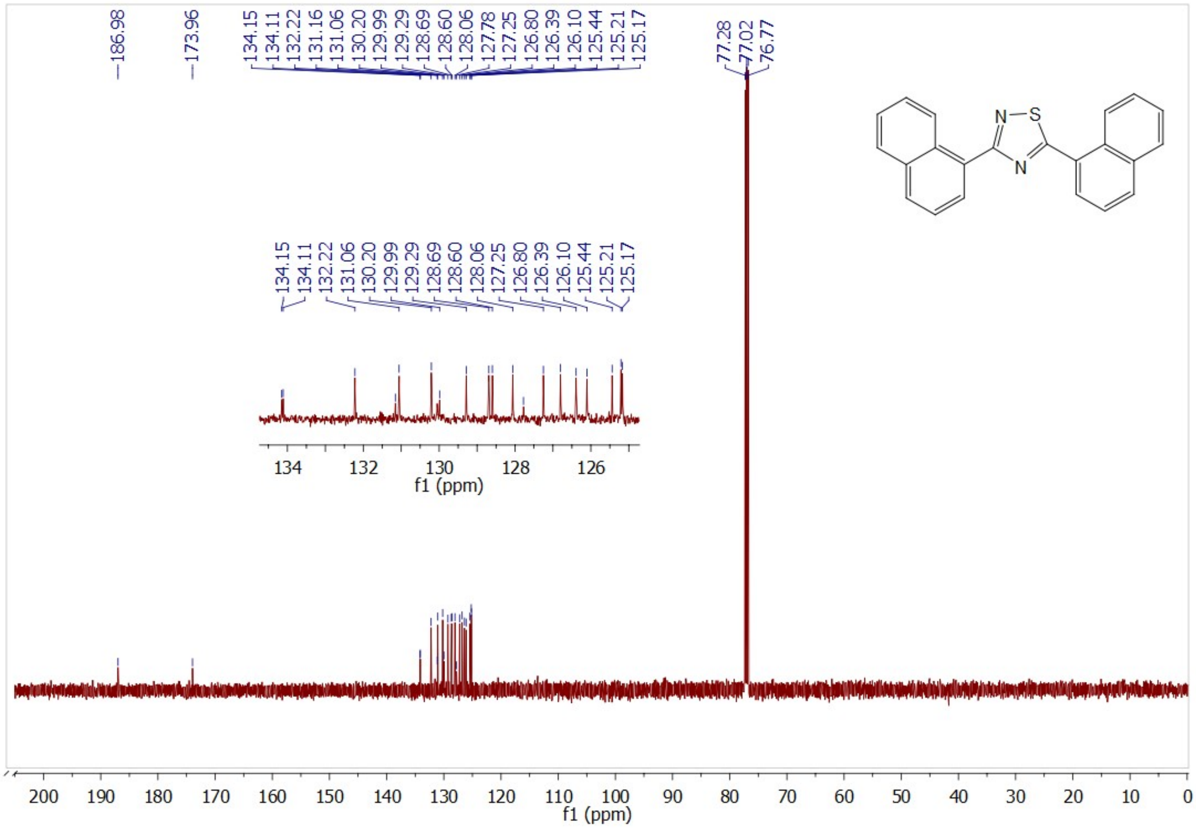

[4.2.20] 3,5-dimethyl-1,2,4-thiadiazole in $CDCl_3$ (3t)

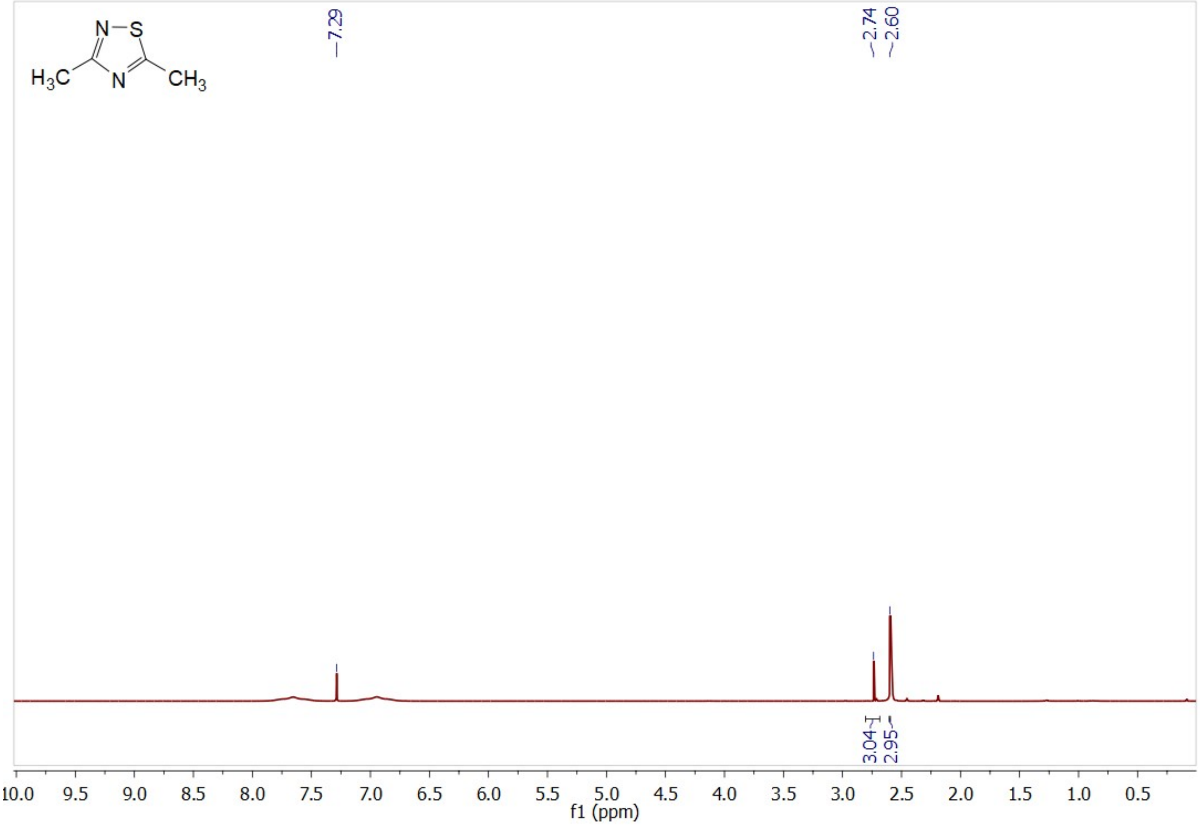

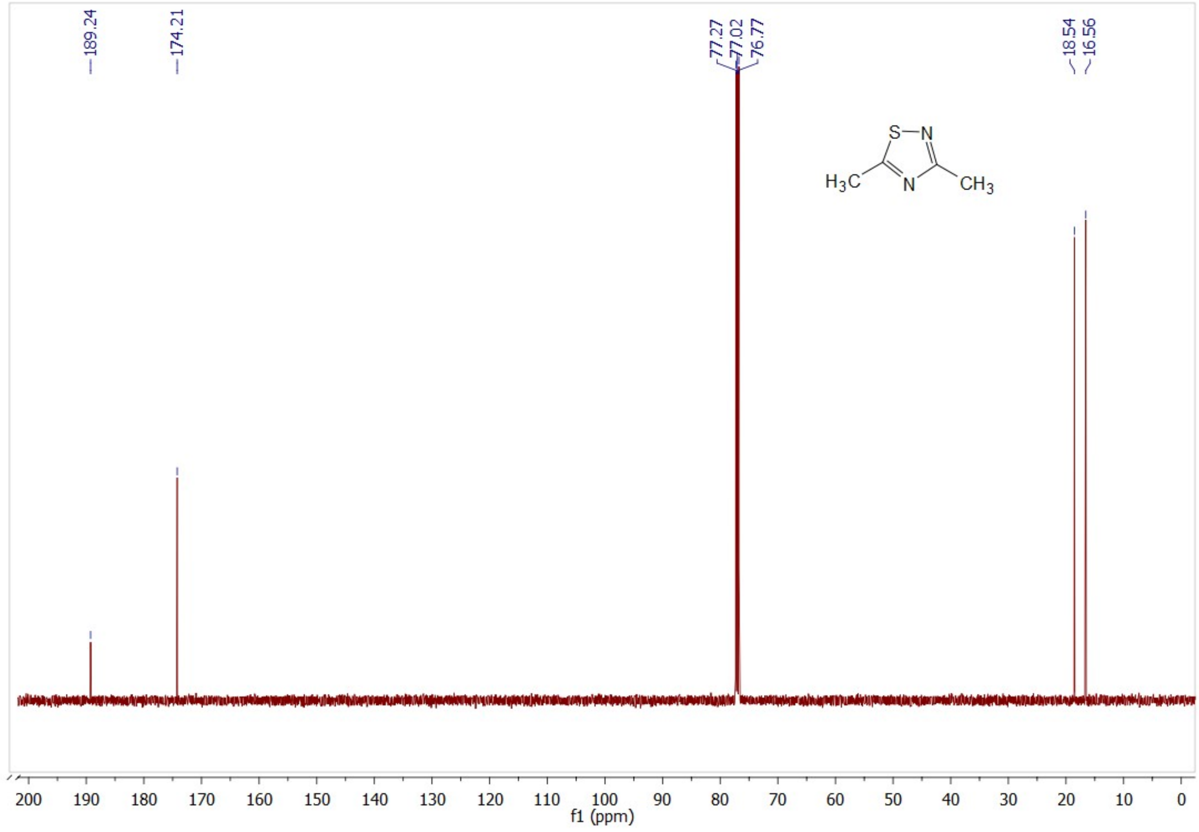

[4.2.21] 3,5-di-tert-butyl-1,2,4-thiadiazole in $CDCl_3$ (3u)

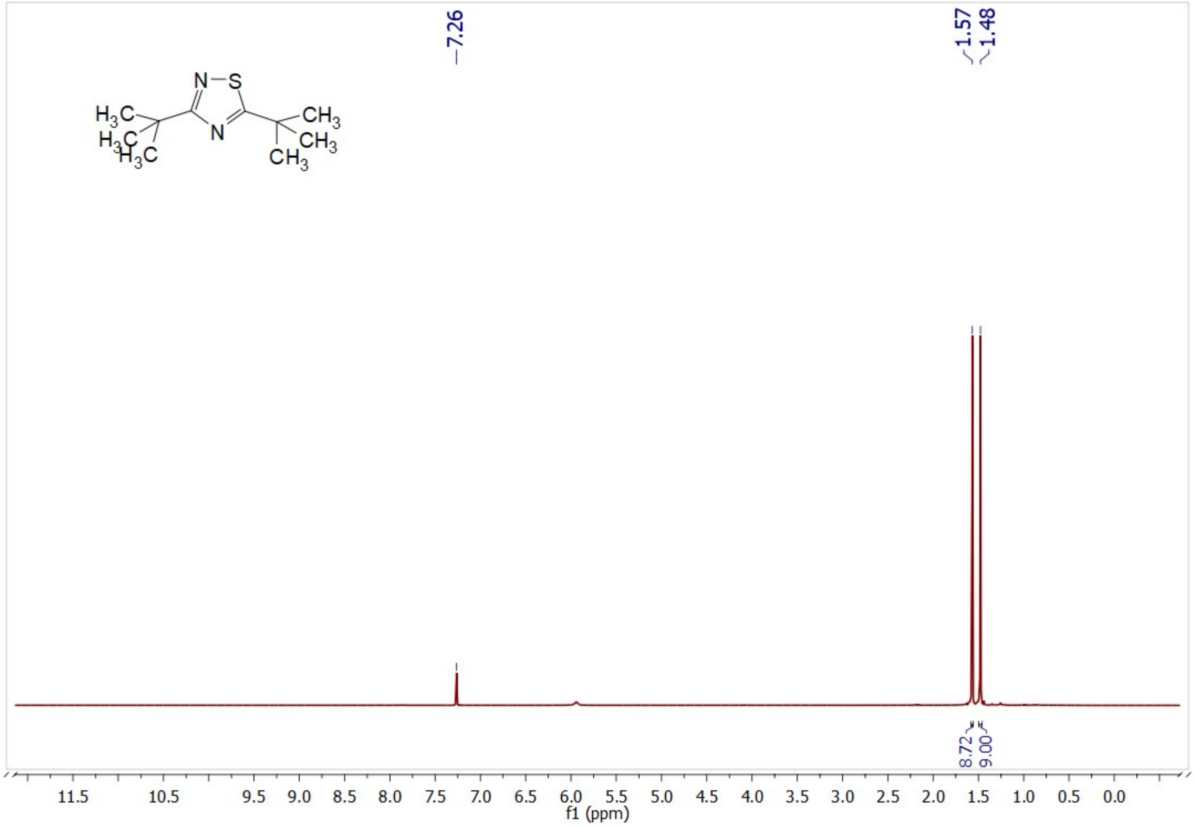

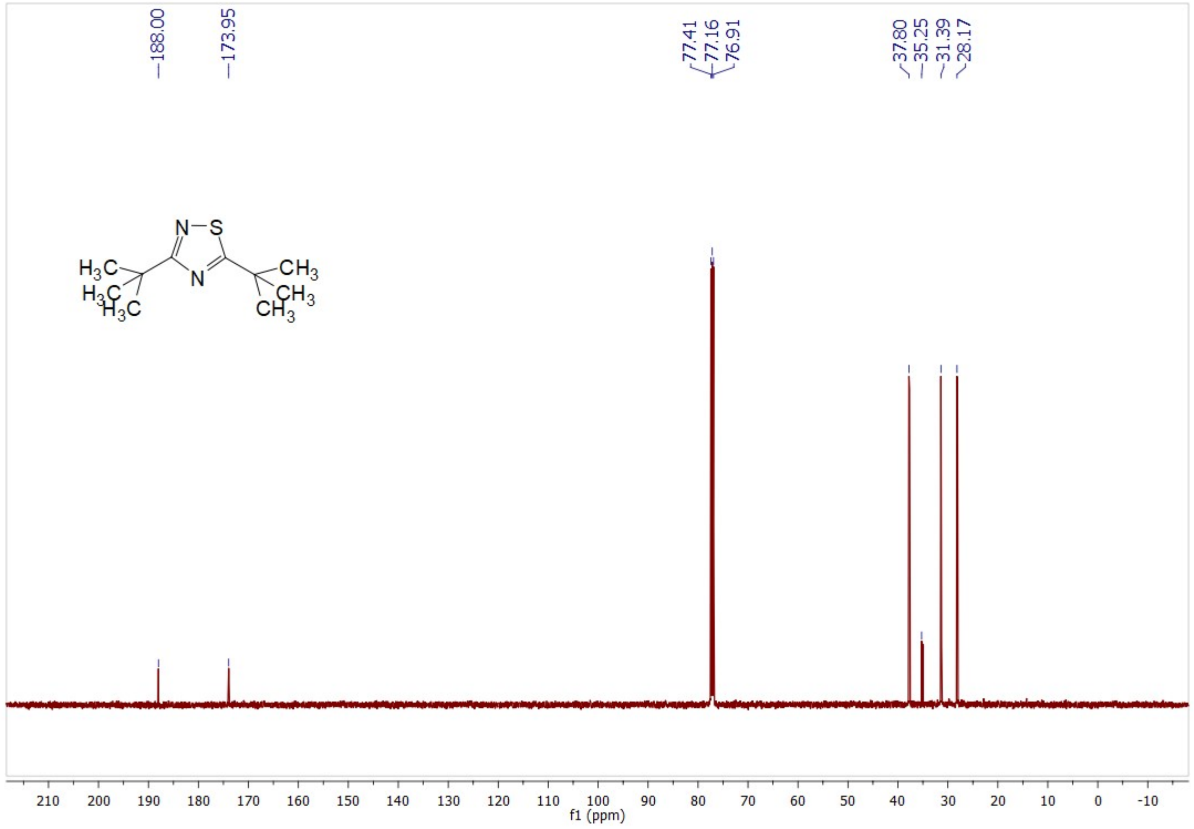

[4.2.22] 3,5-dipiperidine-1-yl-1,2,4-thiadiazole in $CDCl_3$ (3v)

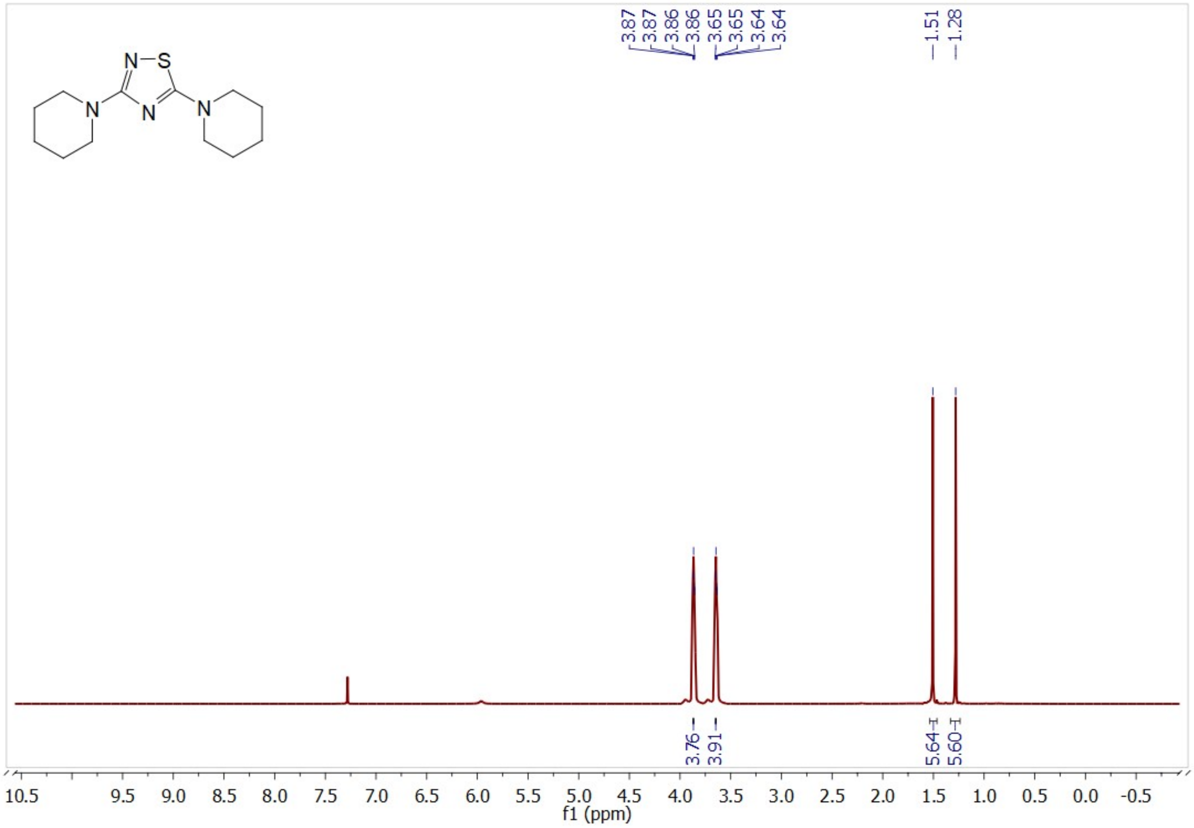

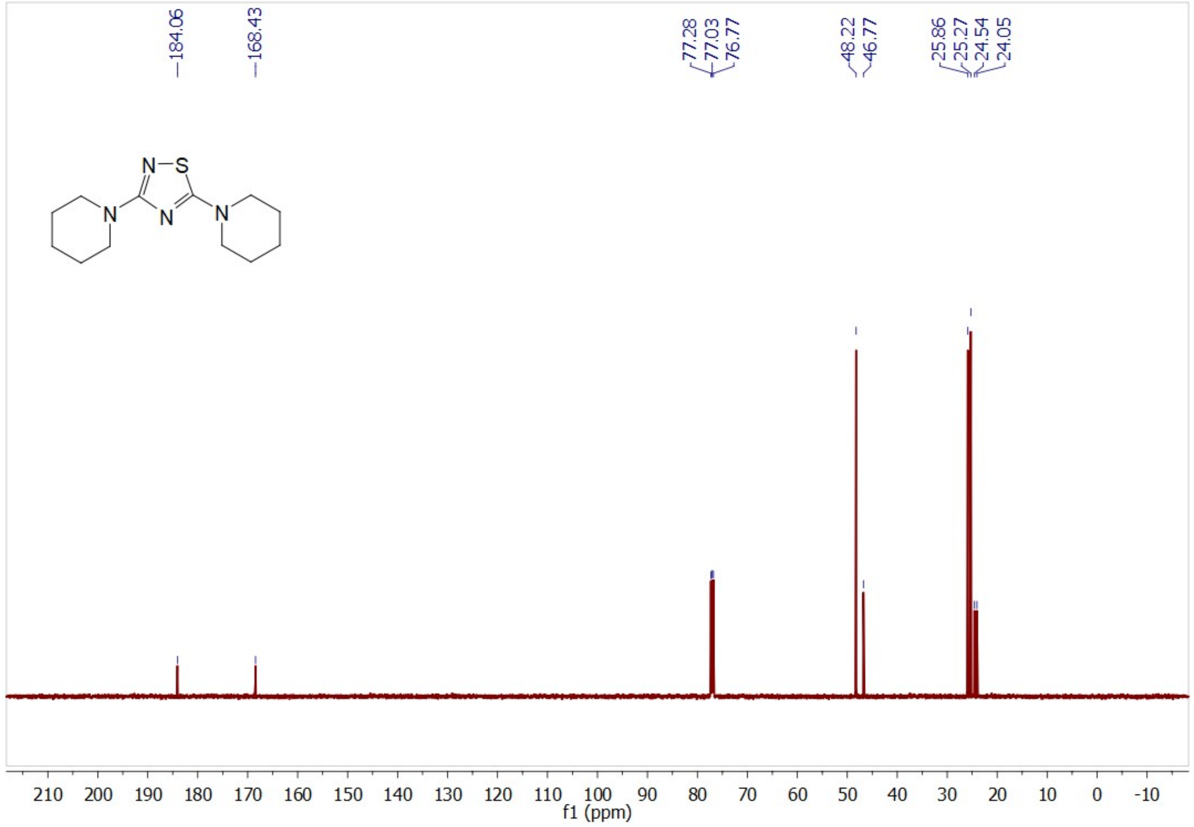

[4.2.23] 3,5 –dimorpholine-4-yl-1,2,4-thiadiazole in $CDCl_3$ (3w)

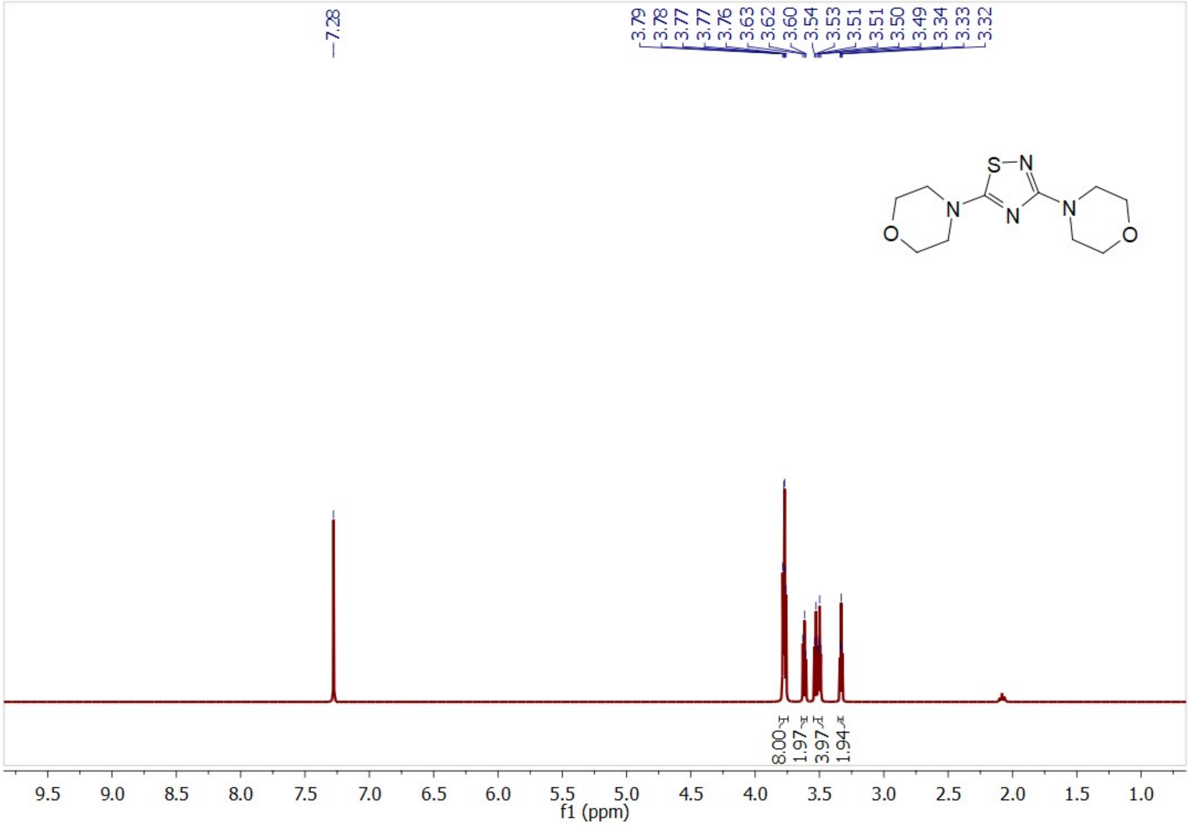

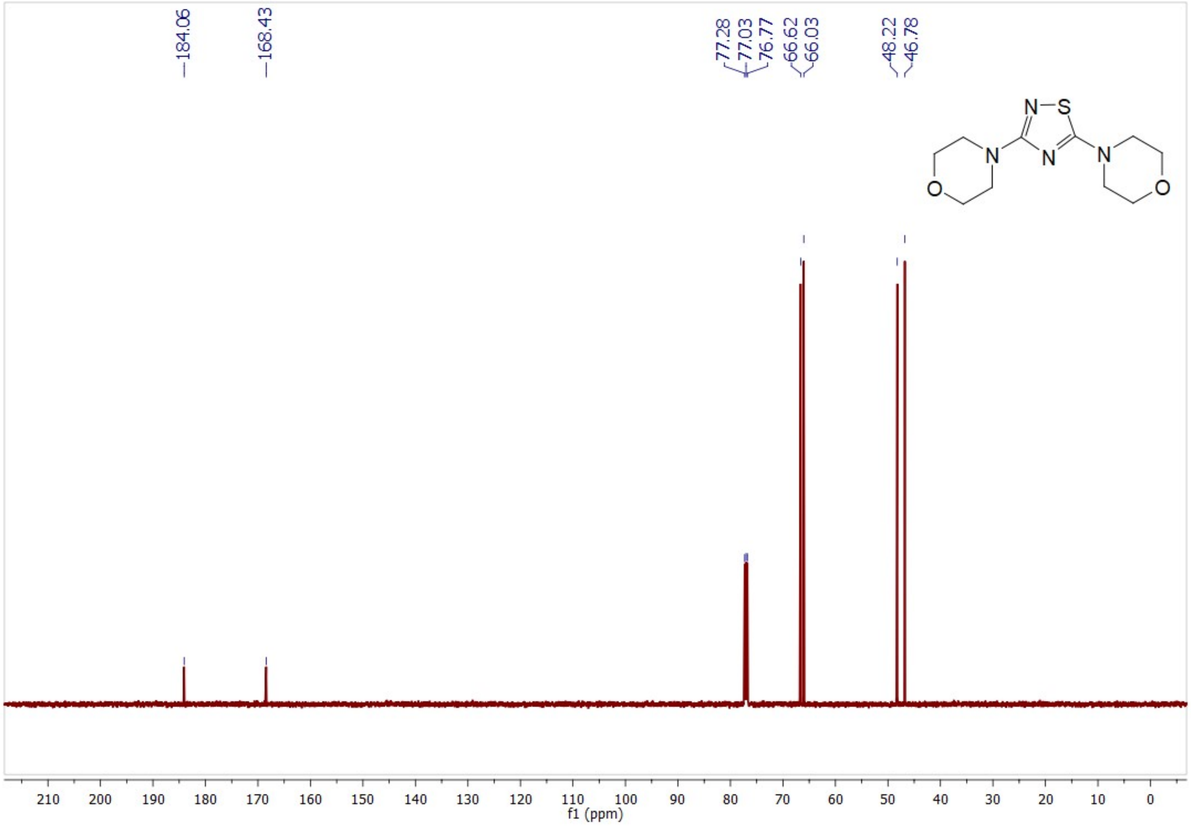

[4.2.24] 3,5 –dibenzyl-1,2,4-thiadiazole in $CDCl_3$ (3x)

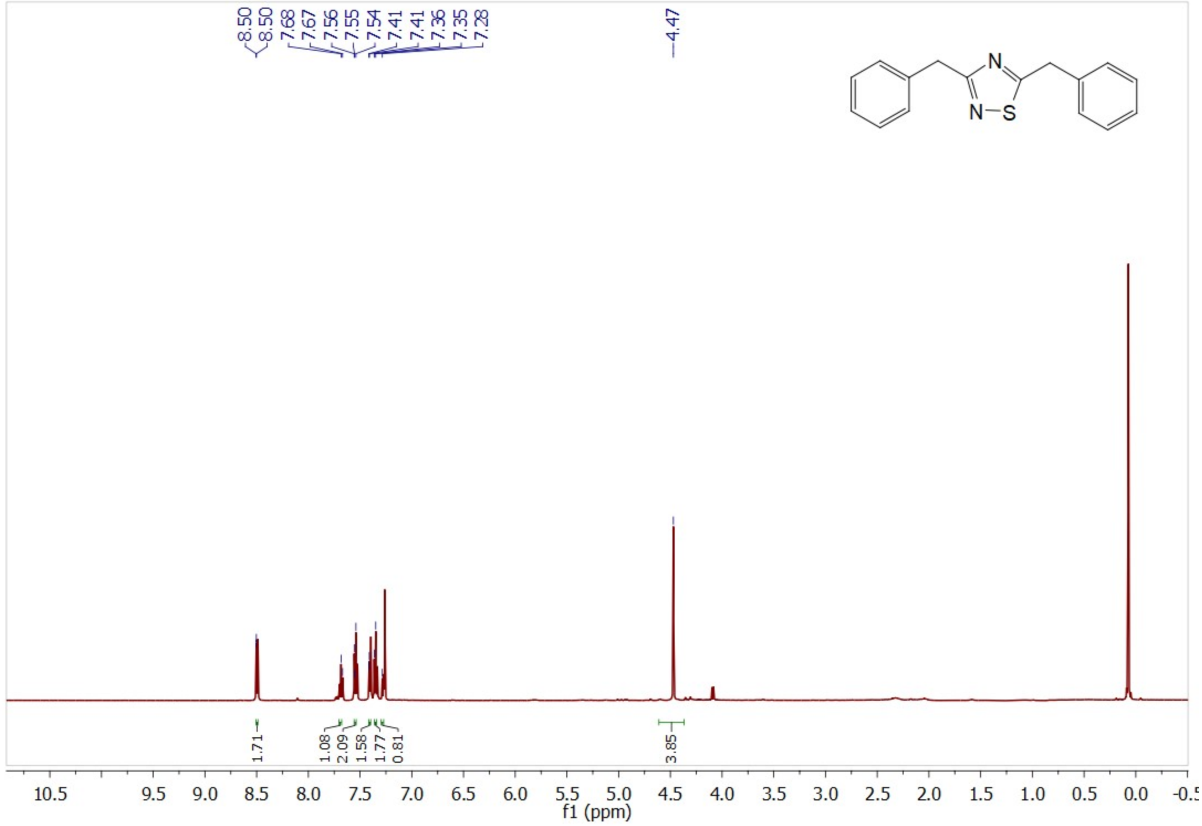

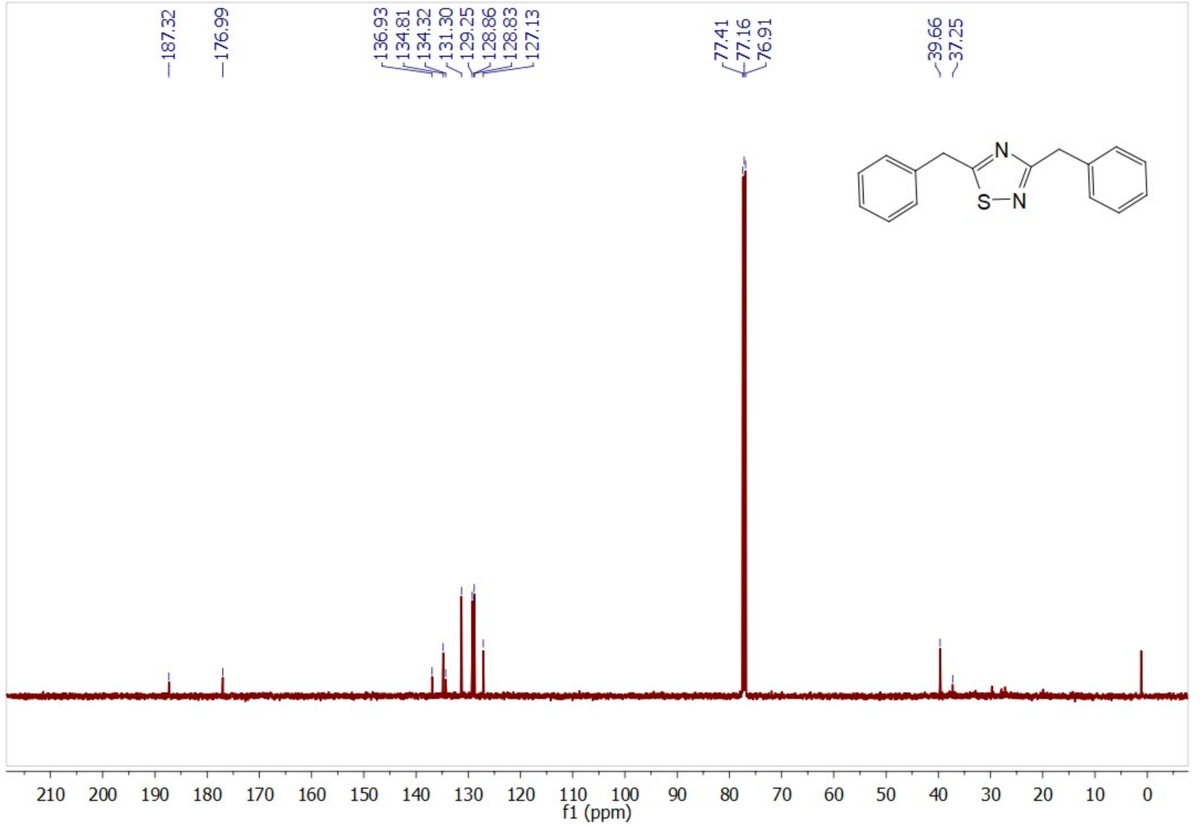

[4.1.25] 3,5-bis(4-chloro-benzyl)-[1,2,4]thiadiazole (3y)

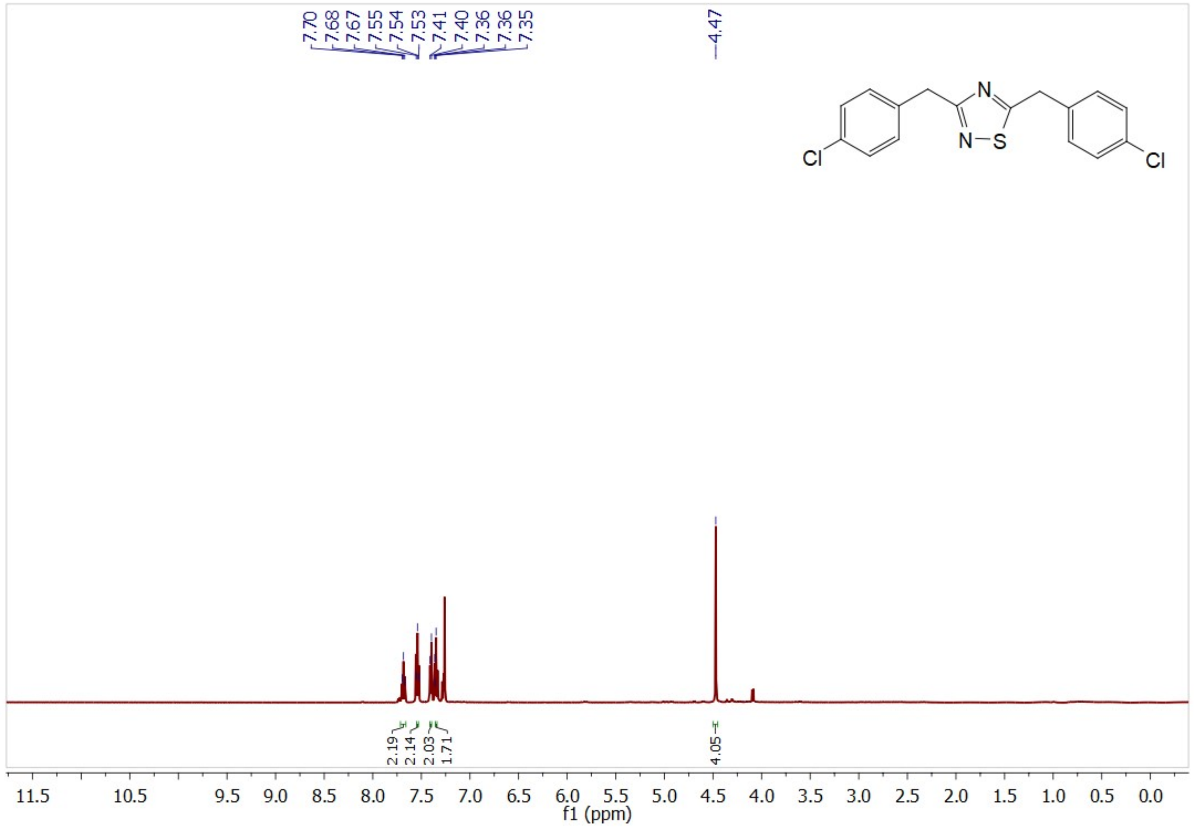

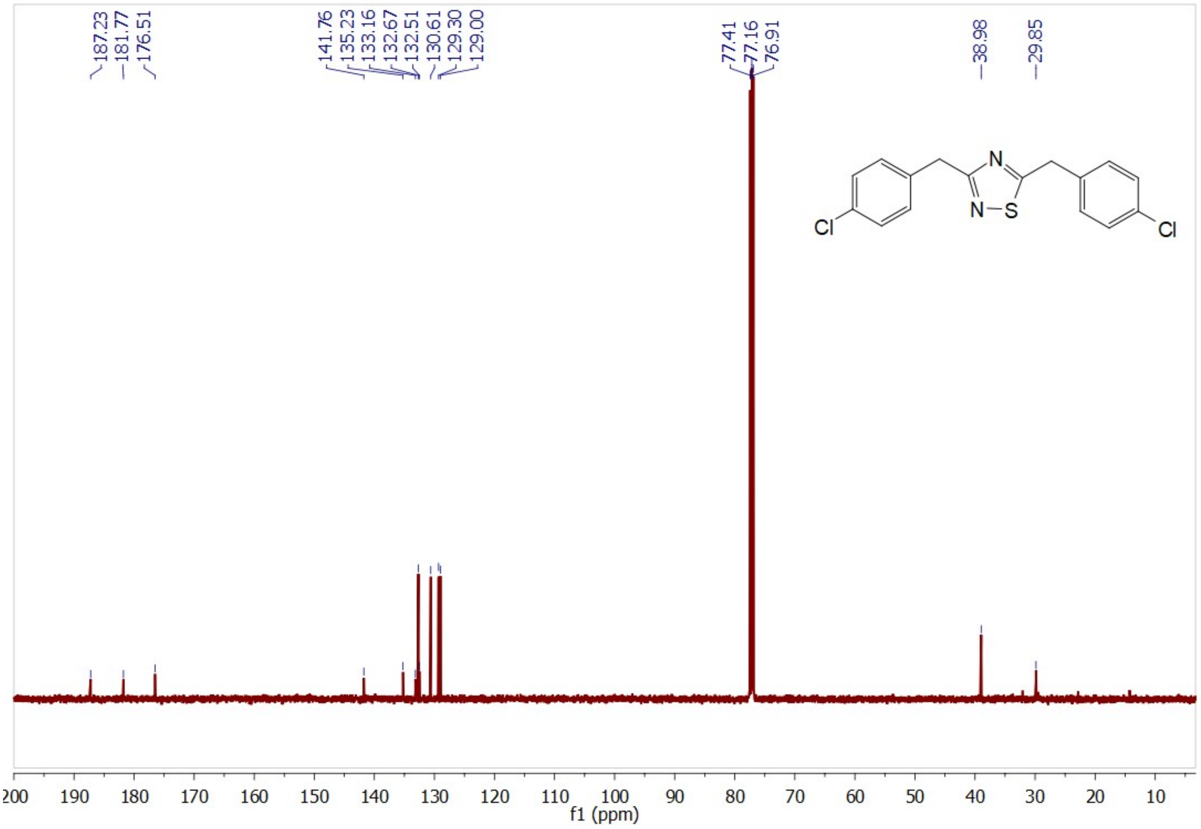

[4.2.26] 3,5-Bis(3-pyridinyl)-1,2,4-thiadiazole in $CDCl_3$ (3z)

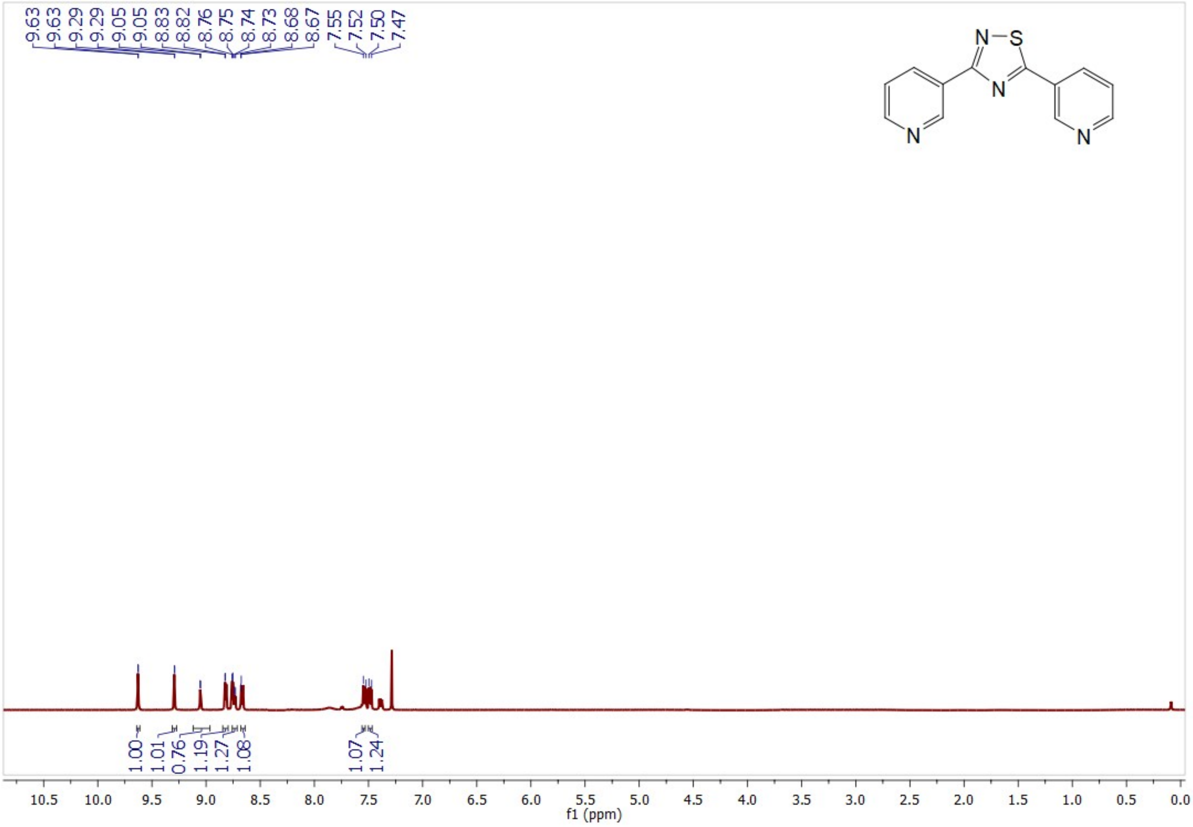

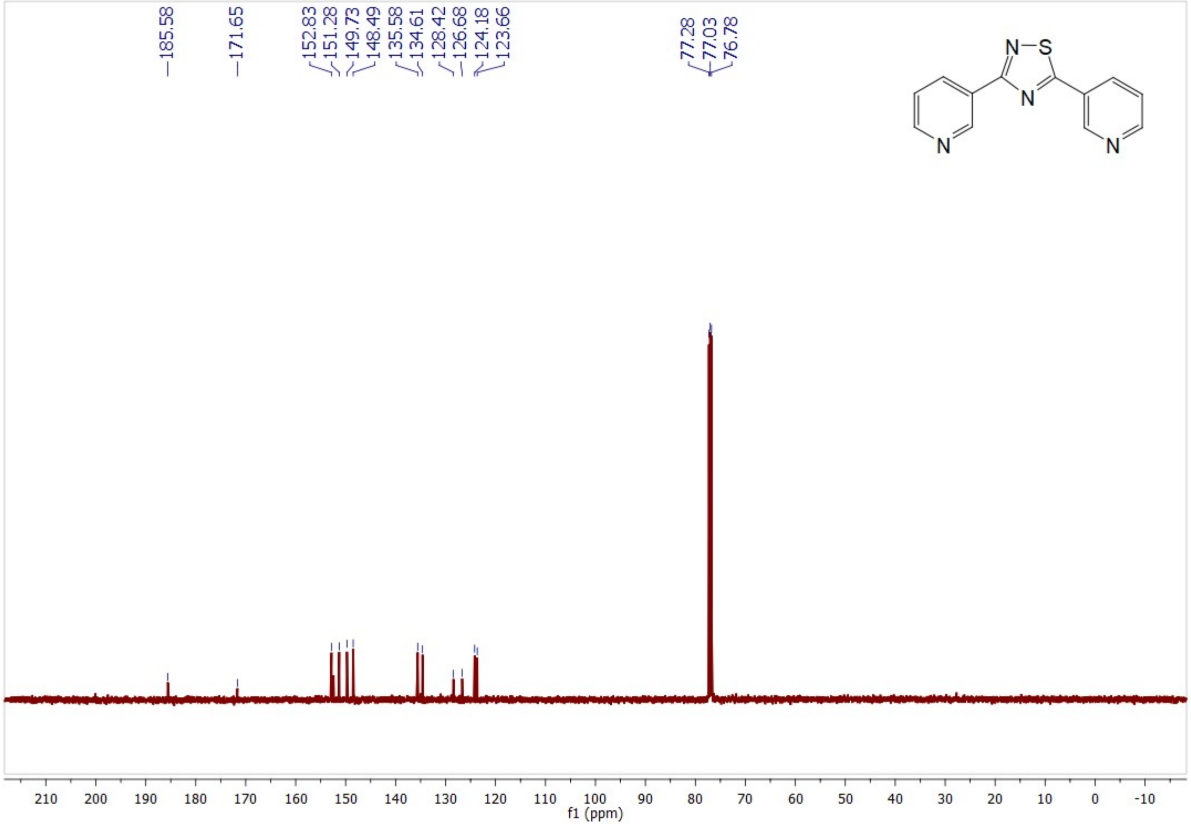

^{1}H &^{13}C NMR of 5a & 5b in $CDCl_3$

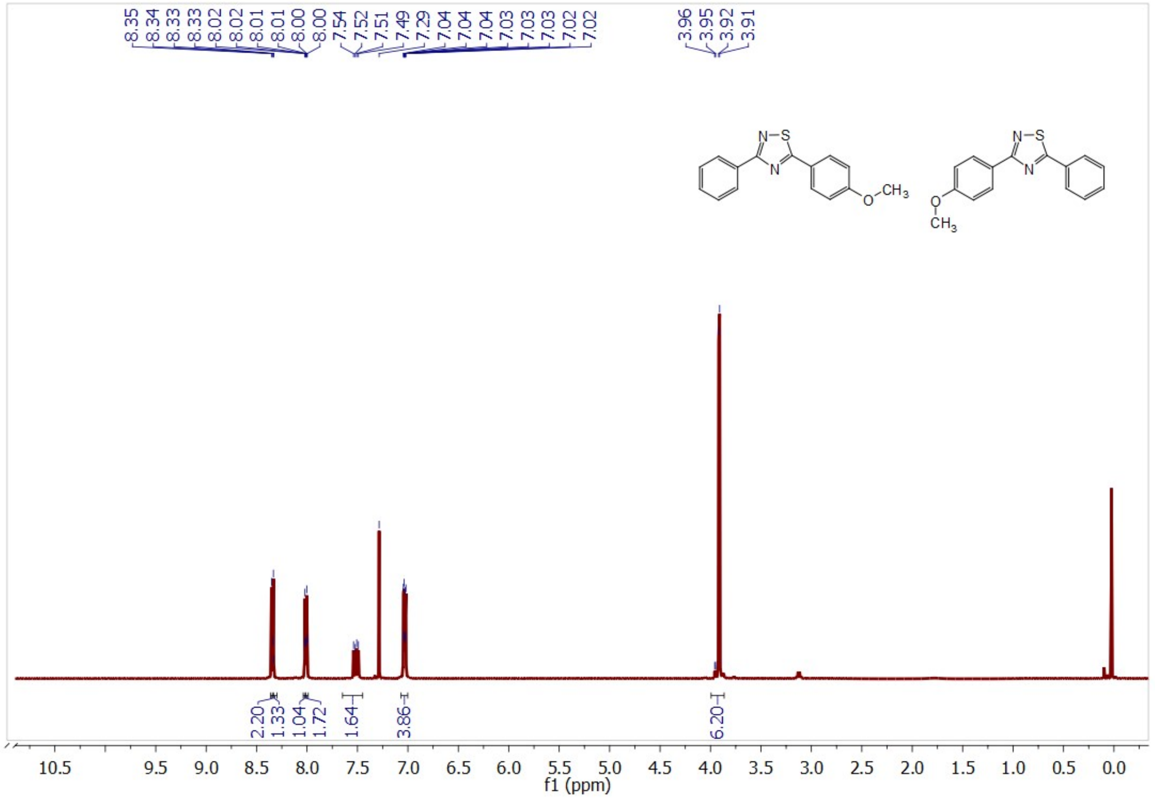

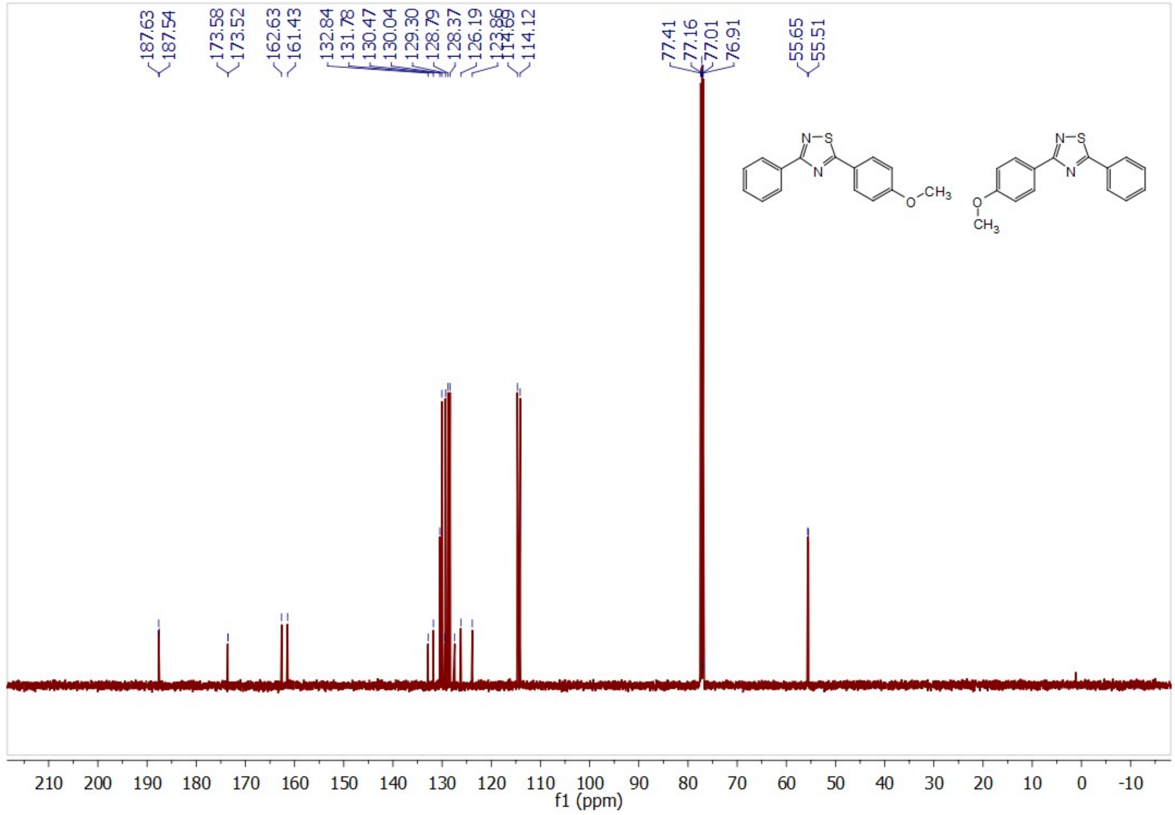

Supplement: RA-014-D4RA03993A-s001 [file RA-014-D4RA03993A-s001.pdf]
